# Field evidence for two paths to cross-cultural competence: implications for cultural dynamics

**DOI:** 10.1017/ehs.2020.1

**Published:** 2020-02-07

**Authors:** John A. Bunce

**Affiliations:** Max Planck Institute for Evolutionary Anthropology, Deutscher Platz 6, 04103 Leipzig, Germany and Department of Anthropology, University of California, Davis, One Shields Ave, Davis, CA 95616, USA

**Keywords:** Cultural evolution, cross-cultural competence, norms, ethnicity, Amazonia, item response theory

## Abstract

Interaction between members of culturally distinct (ethnic) groups is an important driver of the evolutionary dynamics of human culture, yet relevant mechanisms remain underexplored. For example, cultural loss resulting from integration with culturally distinct immigrants or colonial majority populations remains a topic whose political salience exceeds our understanding of mechanisms that may drive or impede it. For such dynamics, one mediating factor is the ability to interact successfully across cultural boundaries (cross-cultural competence). However, measurement difficulties often hinder its investigation. Here, simple field methods in a uniquely suited Amazonian population and Bayesian item–response theory models are used to derive the first experience-level measure of cross-cultural competence, as well as evidence for two developmental paths: cross-cultural competence may emerge as a side effect of adopting out-group cultural norms, or it may be acquired while maintaining in-group norms. Ethnographic evidence suggests that the path taken is a likely consequence of power differences in inter- vs intra-group interaction. The former path, paralleling language extinction, may lead to cultural loss; the latter to cultural sustainability. Recognition of such path-dependent effects is vital to theory of cultural dynamics in humans and perhaps other species, and to effective policy promoting cultural diversity and constructive inter-ethnic interaction.

## Graphical abstract

**Figure:**
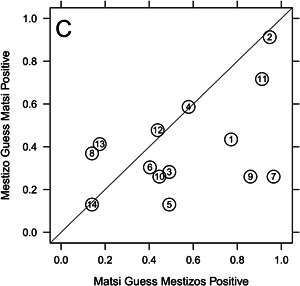

**Media summary:** Fieldwork suggests two paths to develop cross-cultural competence; one may promote cultural sustainability, the other loss.

The last 40,000 years of human evolutionary history are characterized by a diversity of behavior without precedent in nature (Foley and Lahr 2011). This behavioral diversity, often structured in symbolically marked groups (e.g. ethnic groups; Barth 1998), is a consequence of our evolved dependence on both individual and social learning for the acquisition of many skills and beliefs that have been, on balance, adaptive in the varying socioecological conditions encountered and created as our ancestors spread across the planet (Henrich and McElreath 2003; Richerson and Boyd 2005). Much of the richness of human history derives from interaction between members of such culturally distinct ethnic groups, be it mutually beneficial exchange, inter-marriage or exploitation and violence (Wolf 1982; Sahlins 1994). However, at present, we have only a rudimentary understanding of mechanisms underlying the cultural dynamics that often accompany such inter-ethnic interaction, entailing adoption of both adaptive and non-adaptive group-typical (i.e. cultural) behaviors and beliefs across group boundaries (Bunce and McElreath 2017). We thus require a more comprehensive theory of cultural dynamics at ethnic boundaries in order to better understand patterns in the linked genetic and cultural evolutionary history of our species (e.g. the spread of dairying culture and lactase persistence; Ségurel and Bon 2017). Importantly, such theory may also shed light on the foundations of some contemporary societal problems.

For instance, fears of cultural loss or dilution as a consequence of inter-ethnic interaction form an important component of both domestic and international politics, and appear to be especially salient in recent discourse. They have motivated international protections for minority indigenous cultures undergoing social integration (United Nations General Assembly 2007), as well as nationalistic rhetoric decrying the influence of immigrants on host nation culture (Akkerman and Hagelund 2007; Betz and Meret 2009; Rydgren 2007; Golder 2016). The effect of such inter-ethnic interaction on cultural maintenance or change is likely to depend crucially on individuals who can interact successfully on both sides of an ethnic boundary. For instance, studies of language contact show that bilingual individuals can play a critical role in both the rate and direction of language change (Kandler *et al.* 2010; Fernando *et al.* 2010; Lambert 1981). However, current concern over cultural sustainability usually also involves perceived threats to valued non-linguistic culture-specific norms of coordination, such as beliefs about what constitutes appropriate behavior in a given context (Bunce and McElreath 2017). Politically salient examples of such perceived threats include conflicting norms of gender relations (Akkerman and Hagelund 2007), democratic participation (Betz and Meret 2009) and pedagogy (Trapnell 2003; United Nations General Assembly 2007). Interaction between members of groups with incompatible norms can result in costly failures (Sahlins 1994), as well as the eventual loss of one group's distinctive norms (Bunce and McElreath 2017, 2018). This perceived loss, when associated with group identity, can lead to both political backlash and harm to individual psychological health (Berry 1997).

As a generalization of the concept of bilingualism to include such non-linguistic cultural norms, I here use the term cross-cultural competence – the ability to interact successfully using both in-group and out-group norms. Knowledge of norms and values important to the out-group is a characteristic of peaceful multiethnic societies (Wise and Velayutham 2014), and is a potentially vital component of inter-group coordination to avoid violent conflict (Ginges *et al.* 2007). Cross-culturally competent individuals are expected to have the ability to consciously recognize and compare such out-group norms with the norms of their own group, i.e. they manifest a cultural metacognition that may entail cultural perspective-taking (Mor *et al.* 2013). Like bilingualism, general cross-cultural competence probably plays an important role in cultural maintenance and change. However, despite considerable attention in psychology (Hong *et al.* 2000; Berry 1997), medicine (Anand and Lahiri 2009), business (Johnson *et al.* 2006) and inter-cultural education (Trapnell 2003), and its importance in the historical record (Lamana 2008) and contemporary lived experience (Wise and Velayutham 2014; Kopenawa and Albert 2013), cross-cultural competence is conspicuously absent in all but a few (Kuran and Sandholm 2008; Carvalho 2017) studies of cultural dynamics (Bunce and McElreath 2018; Boyd and Richerson 2009; Creanza *et al.* 2017; Erten *et al.* 2018; Mesoudi 2018), primarily because it both complicates theoretical models and is notoriously difficult to operationalize and measure (Spitzberg and Changnon 2009; Fantini 2009).

## Measuring cross-cultural competence

**Figure 1.**
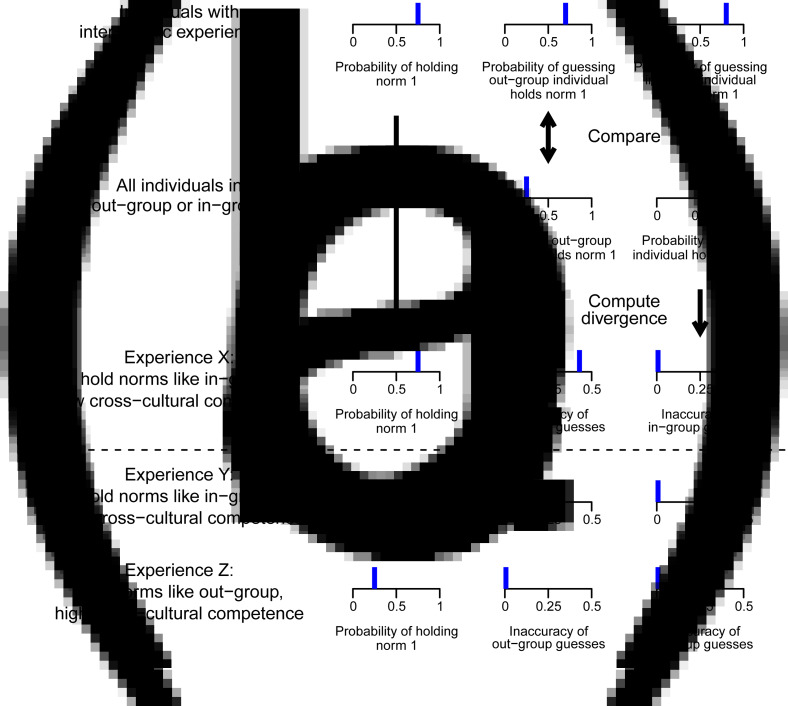
Derivation of experience-level cross-cultural competence. (a) Determine the probability that individuals with a given type of inter-ethnic experience hold a given norm, as well as their guesses about the norm held by an anonymous, randomly chosen out-group and in-group member. (b) Compare guess probabilities with the actual probabilities that out-group and in-group individuals hold the norm, and compute Kullback–Leibler divergence (Kullback and Leibler 1951) between these probability distributions as a measure of guess inaccuracy (in natural units of information entropy – see Materials and Methods). (c) Relative cross-cultural competence for an experience type is defined as less inaccurate out-group guesses and no more inaccurate in-group guesses than individuals with other types of experience. Two paths to cross-cultural competence (Y and Z) are distinguished by the norms that individuals hold.

One of the difficulties of measuring cross-cultural competence is that, unlike bilingualism, it must account for uncertainty in individual norms. For instance, although all members of an ethnic group may be able to communicate using the same language (despite within-group speech variants: Labov *et al.* 2016), rarely do they all hold exactly the same norms in other domains (Bunce and McElreath 2017). Thus, when deciding how to behave with unfamiliar in-group and out-group members, a cross-culturally competent individual must employ heuristics about the probability distributions (commonness) of particular norms in the respective groups. Figure 1 shows how such heuristics, manifesting as guesses about the norms held by in-group and out-group members, can be used to derive an experience-level measure of cross-cultural competence. Different experiences with out-group norms may be associated with different degrees of relative cross-cultural competence, which is indicated by lower inaccuracy of out-group guesses and no greater inaccuracy of in-group guesses. Here, guess accuracy is used as a proxy (i.e. a necessary but not sufficient condition) for the ability to interact successfully using those norms.

Figure 1 also shows that this measure distinguishes between two forms of cross-cultural competence, which differ in whether an individual personally holds norms typical of the in-group (Experience Y) or typical of the out-group (Experience Z). As discussed below, these two forms appear to have profoundly different implications for the sustainability of valued cultural traits within a multiethnic society. A norm that is personally held is here defined as that which an individual would prefer to employ when interacting with a(nother) cross-culturally competent individual capable of employing either norm. This preference could be associated with an internalized injunctive norm or with a pragmatic descriptive norm (Morris *et al.* 2015; Bicchieri 2006) contingent on the contextual cues present during an individual's most frequent or most important coordination interactions in that domain.

Measuring cross-cultural competence in this way requires several additional assumptions. First, we must assume that an individual's personally held norms can be investigated through her/his responses to ethnographically informed interview questions. Care must taken in interpretation of such data, as the norms people express in response to interview questions may, as a result of perceived social pressure, not necessarily correspond to their actual behavior (Cronk 1991). Furthermore, people's perceptions of which norms are widely distributed within their own society, and, therefore, the norms it behooves them to follow in public, may bear little resemblance to the actual distributions of the norms that people in the society personally hold. In the extreme case, known as pluralistic ignorance (Katz and Allport 1931; Miller and McFarland 1987; Prentice and Miller 1993), the norm believed to be widely held, and followed, by most members of a society conflicts with the norm that those same individuals hold. Cultural competence in such a context would require knowledge of the norms that people in a society believe to be widespread (intersubjective cultural knowledge: Chiu *et al.* 2010), rather than knowledge of the commonness of people's personally held norms. The measure of relative cross-cultural competence described here is applicable only under the assumption that knowing a person's personally held norm provides information about how they are likely to behave. When most people's guesses of in-group norms are highly inaccurate, pluralistic ignorance is possible and this assumption may not hold. In such cases, the measure described here should be used with caution.

The goal of the present exploratory study is to demonstrate the applicability of the above measure by using it to characterize patterns of cross-cultural competence in an indigenous Matsigenka community and neighboring communities of Mestizo colonists in Amazonian Peru. Results of the quantitative analysis are checked against ethnographic observations in these communities, and discussed in light of long-term consequences for the maintenance or loss of group-typical cultural norms. This empirical examination of cross-cultural competence sheds light on the understudied, and important, role it may play in human cultural dynamics, and perhaps those of other cultural species.

## Methods

### Overview

An interview comprising fourteen ethnographically informed vignette questions (Table 1) measured personally held cultural norms across a range of interaction domains among adult residents of a Matsigenka community and neighboring Mestizo communities. A subset of these interviewees was then asked to guess the most common personally held norm (i.e. the response of a randomly chosen member) in their own ethnic group (in-group) and in the other ethnic group (out-group) for each vignette question, similar to previous methods for measuring inter-group perceptions (Medin *et al.* 2007; Gurven *et al.* 2008). Based on ethnographic observations collected over a year (Matsigenka) and five months (Mestizos) of participation in community life, domains of salient inter-ethnic interaction (e.g. education, labor) were identified and interviewees’ self-reported experience in each domain was recorded. To distinguish among the associations between these inter-ethnic experiences and cross-cultural competence, data were analyzed using Bayesian estimation of item–response theory (IRT) models (Bunce and McElreath 2017), which resulted in posterior distributions (i.e. model estimates with associated uncertainties) of the probabilities of holding particular norms and guessing the most common norms held by in-group and out-group members, for each experience type. Using the procedure illustrated in Figure 1, the degree and form of cross-cultural competence associated with each inter-ethnic experience type were then compared.

**Table 1.** Vignette questions administered in this study, and their respective social contexts. Column four contains the response arbitrarily coded as 1 (positive). An alternative response was coded as 0. The number of Matsigenka and Mestizo interviewees answering each question is indicated in column 5. The upper row corresponds to the number of Matsigenka responses with regard to personally held norms (ego, E), in-group guesses (I) and out-group guesses (O). The lower row contains analogous sample sizes for Mestizos. Further explanation, ethnographic validation, and translations of these questions are provided in Supplementary Appendix A.2

Number	Social context	Question	Positive	*N* (Mat, Mes)
1	Spousal relations	There is a married couple with no children. The woman hunts and fishes (Mestizo: has a job and makes more money). The man stays home and cooks, weaves (Mestizo: cleans), and washes clothes. Is this okay or not okay?	Okay	E, I, O 70, 57, 57, 80, 46, 46
2	Parent–offspring relations	After school, a 10-year-old daughter cannot go to a friend's house to play because she has to care for her two-year-old brother until their parents come home at night. Is this okay or not okay?	Okay	77, 57, 57, 78, 46, 46
3	Inheritance	A man always wears his favorite hat. After he dies, his son takes the hat and wears it. When he wears it he remembers his father. Is this okay or not okay?	Not okay	74, 57, 57, 79, 46, 46
4	Spousal relations	A woman wants to drink alcohol, but her husband doesn't want to drink. So the wife goes to drink without her husband. Is this okay or not okay?	Okay	73, 57, 57, 80, 46, 46
5	Education	A teacher hits students when they don't learn. Is this okay or not okay?	Okay	75, 57, 57, 74, 46, 46
6	Education	A student pays attention to the teacher and never asks any questions. Is this okay or not okay?	Okay	79, 57, 57, 81, 46, 46
7	Healthcare	If you get a respiratory illness (influenza), do you first go to the health post, first use home remedies, or first go to a shaman or curandero?	Health post	79, 57, 57, 81, 46, 46
8	Healthcare	You have chest pain (Mestizos: You wake up one day with chest pain). Do you first go to the health post, first use home remedies, or first go to a shaman or curandero?	Not health post	78, 57, 57, 79, 46, 46
9	Fairness	An old woman has two new pots and two adult daughters. One daughter has her own two pots, but wants her mother's pots. The other daughter has no pots, and also wants her mother's pots. When the mother dies, who should inherit the pots? Illustrated with a diagram. Options: one pot to each daughter, both pots to the daughter who has none.	Both pots to the daughter who has none	76, 57, 57, 82, 46, 46
10	Religion	A good person does not want to be baptized. Where does his or her soul go when they die? Options: up (heaven), down (hell), somewhere else.	Hell or somewhere else	64, 56, 56, 60, 46, 46
11	Wage labor	A man is hired to prepare an agricultural field. He stops work at noon in order to go visit a friend. He returns the next day to finish the job. Is this okay or not okay?	Okay	75, 57, 57, 77, 46, 46
12	Commerce	There are two stores. One is cheap with a mean owner. The other is expensive with a nice owner. Where would you buy?	Cheap store with mean owner	63, 57, 57, 77, 46, 46
13	Parent–offspring relations	Parents want their daughter to marry a certain man that she does not like. She wants to marry someone else. Should she obey her parents and marry him anyway or not?	Obey parents	63, 57, 57, 81, 46, 46
14	Wage labor	A man is hired to work two days: Monday and Tuesday. Monday night there is a party (Matsigenka: hosted by a Matsigenka). Should he go and get drunk or not? (Matsigenka: He goes and gets so drunk that he can't work on Tuesday. Is this okay or not okay?)	Okay to go and become drunk	67, 57, 57, 80, 46, 46

### Study population

The study was conducted among residents of the Matsigenka Native Community of Tayakome (adult population 79), located inside Manu National Park, in the department of Madre de Dios, in the lowland Amazonian region of southeastern Peru, and in the Mestizo towns of Boca Manu (adult population ~80) and Atalaya (adult population ~65), located just outside the boundary of the park, in the departments of Madre de Dios and Cusco, respectively (see Figure S1). Detailed descriptions of these communities can be found in Bunce and McElreath (2017), Shepard et. al (2010), and Llosa Isenrich and Nieto Degregori (2003). As detailed in Supplementary Appendix A.2, there are salient norm differences between Matsigenka and Mestizos across many domains of life. This population is ideally suited to investigation of cross-cultural competence because, at the time of fieldwork, there was an almost complete absence of mass media exposure to the other ethnic group's cultural norms. This constrained the development of cross-cultural competence to: (a) contexts of personal inter-ethnic experience; and (b) intra-ethnic social learning from those with out-group experience. Such conditions contribute to substantial measurable variation in out-group norm exposure among individuals, which would probably be much less pronounced if norms were learned through mass media.

### Personal norm interviews

I lived in Boca Manu for approximately three months (September and November 2012, January 2014), Atalaya for two months (December 2012 and February 2014) and Tayakome for 13 months (January–December 2013, March 2014). Over several rounds of semi-structured interviews, I recorded interviewees’ life histories and recollections of personal inter-ethnic interaction experience. For Matsigenka, domains of such inter-ethnic experience included education, working as wage laborers and commerce, all with Mestizos. For Mestizos, domains included indigenous family members (e.g. adopted children), hiring Matsigenka wage laborers, and living (previously) in a majority-indigenous community. I then designed a set of vignette questions (Table 1) for the purpose of learning about specific norms in nine contexts of social coordination (commerce, wage labor, education, spousal relations, parent–offspring relations, inheritance, fairness, healthcare and religion) and administered these questions privately to 74 (94%) residents of Tayakome (including the Mestizo health technician), 45 (~56%) residents of Boca Manu (including four Matsigenka), and 42 (~65%) residents of Atalaya (including two Matsigenka), all of whom had been previously interviewed regarding life history and inter-ethnic interaction experience. I refer to this round of interviews as Personal Norm interviews, and people's responses during this interview are referred to as their personally held, or ‘ego’, norms. No interviewee self-identified as bi-cultural, i.e. belonging to both Matsigenka and Mestizo ethnic groups (see also Supplementary Appendix A.3.3). Here and in subsequent rounds of interviews, interviewees were selected out of convenience: I interviewed whoever was available and willing during the days I was present in the communities.

### Guesses about ingroup and outgroup norms

I wrote, on index cards, the Personal Norm responses to each of the 14 vignette questions provided by a sample of 25 interviewees whose responses were representative of the Matsigenka of Tayakome and 24 interviewees whose responses were representative of the Mestizos of Boca Manu and Atalaya, one card for each interviewee. Figure S3 illustrates the representativeness of the cards for the respective communities.

I then re-interviewed 53 residents of Tayakome (all Matsigenka), 26 residents of Boca Manu (including two Matsigenka) and 24 residents of Atalaya (including two Matsigenka), all of whom had participated in Personal Norm Interviews approximately five months prior (Tayakome), 16 months prior (Boca Manu) or one week prior (Atalaya). Owing to people's work schedules and temporary absences from the communities, I was able to re-interview only a subset of all participants who had previously completed Personal Norm Interviews. I briefly reminded interviewees about the previous Personal Norm Interviews in which I had asked them the 14 vignette questions. I then repeated each question one by one. After each, I asked the interviewee to guess how the majority of people in their own community had answered each question (in-group guess), and how the majority of people in the other ethnically distinct community had answered each question (out-group guess). The out-group for both Mestizo communities of Boca Manu and Atalaya was the Masigenka community of Tayakome. The out-group for Tayakome residents was a generic Mestizo from either Boca Manu or Atalaya. For the four Matsigenka interviewees who resided in Boca Manu and Atalaya, the in-group was Tayakome and the out-group was the Mestizo community in which they lived. Thus, in-group and out-group designations used here reflect the emphasis of this study on self-identified ethnicity and do not necessarily reflect feelings of community-belonging on the part of interviewees.

To motivate serious guesses, after an interviewee had made both an in-group and an out-group guess for a given question, I presented her with the two stacks of index cards upon which Personal Norm responses representative of the Matsigenka and Mestizo communities had been written, and explained how the cards had been generated. The cards were shuffled and presented face-down, and the interviewee was asked to select one card from the Matsigenka stack, and one from the Mestizo stack. After each card was chosen, I compared the answer written on the card for the appropriate question with the interviewee's in-group guess (or out-group guess, as appropriate) for that question. I verbally narrated this comparison and, if the guess corresponded to the answer on the card, I congratulated the interviewee on winning 0.5 Peruvian Nuevos Soles (~US$0.15) for that question. If the guess did not correspond to the answer on the card, I explained that she did not win money for that question. Further description of data collection methodology, as well as translations of vignette questions, are provided in Supplementary Appendix A.2. General characteristics of the participants are provided in Table 2.

**Table 2.** Characteristics of the participants in this study. Columns 3 and 4 contain numbers of participants in Personal Norm interviews and Guess (both in-group and out-group) interviews, respectively. For the first four categories, proportions of interviewees per interview type (Personal Norm or Guess) are given in parentheses. For the last two categories, proportions of Matsigenka or Mestizo interviewees (respectively) per interview type are given in parentheses. Note that each individual can have multiple types of experience, so proportions in these last two categories do not sum to 1. Definitions of characteristics, as well as additional details, are provided in Supplementary Appendix A.3.3

Category	Characteristic	Personal Norm interview	Guess interview
Ethnicity	Matsigenka	79 (0.49)	57 (0.55)
	Mestizo	82 (0.51)	46 (0.45)
Residence	Tayakome	74 (0.46)	53 (0.52)
	Boca Manu	45 (0.28)	26 (0.25)
	Atalaya	42 (0.26)	24 (0.23)
Sex	Female	81 (0.5)	55 (0.53)
	Male	80 (0.5)	48 (0.47)
Age	Adolescent	9 (0.06)	5 (0.05)
	Adult	119 (0.74)	76 (0.74)
	Elder	33 (0.2)	22 (0.21)
Experience of Matsigenka	Education	17 (0.22)	10 (0.18)
	Labor	37 (0.47)	26 (0.46)
	Commerce	65 (0.82)	47 (0.82)
Experience of Mestizos	Community	31 (0.39)	19 (0.41)
	Employer	56 (0.68)	34 (0.74)
	Family	36 (0.44)	21 (0.46)

### Statistical analysis

I fit a series of IRT models (Bafumi *et al.* 2005; Jackman 2001; Schacht and Grote 2015; Bunce and McElreath 2017; van der Linden 2016), in a Bayesian framework (McElreath 2016), to interviewees’ Personal Norm (ego) responses, in-group guesses and out-group guesses using the software R (R Core Team 2017) and RStan (Stan Development Team 2018). IRT models are a standard extension of logistic regression, using covariances to construct a representation of individuals’ responses to multiple questions simultaneously (i.e. as positions on a latent axis or axes), as well as characteristics of particular questions, such as their usefulness for discriminating among (i.e. separating) respondents on the latent axis. Responses to the 14 vignette questions for each of the targets (ego, in-group and out-group) co-varied in a single dimension, represented by a latent axis for each target. Models estimated individual-level variance in interviewees’ positions on the three latent target axes (random intercepts), covariance in interviewees’ positions across target axes, covariance in question positions and discriminations within and across target axes, as well as the effects of binary individual-level predictors for inter-ethnic experience in the contexts of education, wage labor worker, wage labor employer, commerce, family and community (see Supplementary Appendix A.3.3). I draw statistical inference from distributions of posterior predictions for the probability of positive responses to the vignette questions (see Table 1) for each target. To represent the inaccuracy of guesses, I calculate Kullback–Leibler (K–L) divergences (Kullback and Leibler 1951) between probabilities of ego responses and in-group and out-group guesses for average (randomly chosen) Matsigenka and Mestizo individuals with different types of inter-ethnic experience (Figure 1). K–L divergence is a standard measure of the additional uncertainty generated when using one probability distribution (e.g. probabilities of a positive and negative guess) to approximate another probability distribution (e.g. probabilities of a positive and negative ego response), and is calculated in units of information entropy (McElreath 2016). Note that this measure of guess inaccuracy applies only in the aggregate, and is not a measure of the inaccuracy of guesses made by individual participants. For instance, the out-group guesses of individual Matsigenka participants are used together to calculate the probability that a randomly chosen Matsigenka would guess that a randomly chosen Mestizo would give the positive response to a given question. The inaccuracy of Matsigenka guesses, in the aggregate, is calculated by comparing this probability with the actual probability that a randomly chosen Mestizo participant would give the positive response. Because each participant provided only one guess per condition (in-group or out-group) per question, it is not possible to calculate such probabilities, and thus inaccuracies, at the individual level. For all analyses, Mestizo residents of Boca Manu and Atalaya are grouped together in the category Mestizos, as the distributions of responses in these two communities are similar (Figure S3). Further details of analysis, including IRT model definitions and priors, K–L divergence calculations and links to data and analysis scripts are provided in Supplementary Appendix A.3.

### Research ethics

I obtained informed consent from all study participants under University of California, Davis IRB 226284-2, and I presented and discussed results during assemblies in each study community prior to publication. Permission to conduct research inside Manu National Park was provided by the Peruvian Servicio Nacional de Areas Naturales Protegidas (SERNANP), with permit numbers 23-2012-SERNANP-PNM-JEF and 23-2013-SERNANP-PNM-JEF.

## Results

### General response characteristics

**Figure 2.**
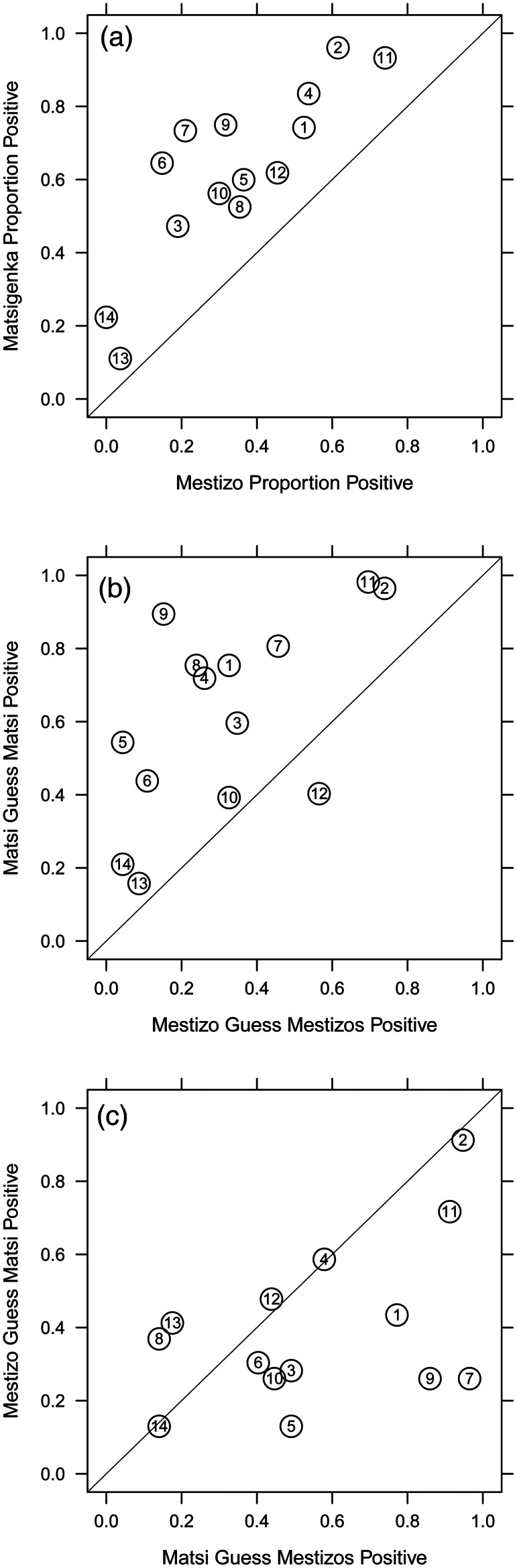
Raw proportions of personally held norms, and in-group and out-group guesses. Proportions of interviewees giving the positive response to the 14 vignette questions in Table 1 are plotted. The diagonal is the line of equal proportions between Matsigenka and Mestizos. The vertical (or horizontal) distance from a point to the diagonal is the difference in proportion between ethnic groups. (a) Personal (ego) Norms for Matsigenka (*n* = 79) and Mestizos (*n* = 82). Note that, for all questions, a larger proportion of Matsigenka than Mestizos gave positive responses, i.e. all points fall above the diagonal. (b) In-group guesses for Matsigenka (*n* = 57) and Mestizos (*n* = 46). Proportions of interviewees who guessed that most members of their in-group gave the positive response to a given question are plotted. Note that if in-group guesses were perfectly accurate in the aggregate for this sample (see Methods: Statistical Analysis), plots (a) and (b) would be identical. (c) Out-group guesses for Matsigenka (*n* = 57) and Mestizos (*n* = 46). Proportions of interviewees who guessed that most members of the out-group gave the positive response to a given question are plotted. Note that if out-group guesses were perfectly accurate in the aggregate for this sample, plots (a) and (c) would be identical.

**Figure 3.**
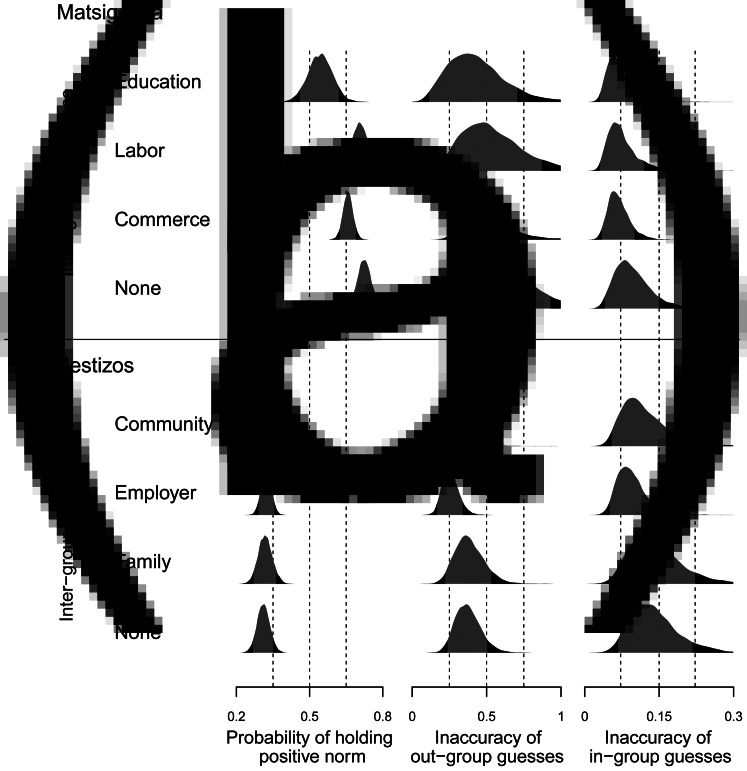
Personally held norms and inaccuracy of in-group and out-group guesses. The four rows of (a) refer to counterfactual (McElreath 2016) (i.e. model estimates of hypothetical) Matsigenka who have interaction experience with Mestizos in only one of the respective domains of education, wage labor or commerce, or none of the three previous domains. Analogously, the four rows of (b) refer to counterfactual Mestizos who have experience in only one of the respective domains of living in an indigenous community, employing indigenous Matsigenka or living in a household with indigenous people, or none of the three previous domains (Supplementary Appendix A.3.3). Left column: posterior distributions (i.e. model estimates with associated uncertainties) of mean probabilities of a positive personally held norm response across all 14 vignette questions (Table 1). Center column: posterior distributions of mean inaccuracy of out-group guesses (calculated as in Figure 1b). Right column: posterior distributions of mean inaccuracy of in-group guesses. Posterior distributions are derived from IRT models (A, m11; and B, m19 in Tables S1 and S2). The 90% highest posterior density intervals (HPDI) are shown in grey.

Figure 2a and the first column of Figure 3 show that Matsigenka and Mestizo participants tended to answer the 14 vignette questions in different ways. For all questions, a larger proportion of Matsigenka than Mestizos gave the response that was arbitrarily coded as positive. This suggests that Matsigenka and Mestizo participants tend to differ in the norms that they personally hold. For example, question 9 dealt with a norm for how to fairly divide an inheritance consisting of two pots among two daughters. One daughter already has her own two pots, while the other has none. Some 68% of Mestizos responded that each daughter should receive one pot, with a typical justification being ‘so that they don't fight’. In contrast, 75% of Matsigenka responded that both pots should go to the daughter who has none, with a typical justification being that ‘the other one already has pots’. Supplementary Appendix A.2 provides ethnographic descriptions of the norms that I attempted to illustrate with each question, as well as typical participants’ justifications for their responses. Figures 2b and c and S9 show that Matsigenka and Mestizo participants tended to guess more accurately about norms personally held by their co-ethnics than about norms held by members of the respective out-group.

There was little evidence of strong pluralistic ignorance among participants for the norms of interest. Inaccuracies of in-group guesses were generally low (see Figure S9), which increases confidence in the appropriateness of the measure of cross-cultural confidence developed here. The most inaccurate in-group guesses are associated with Mestizos on question 5. A randomly chosen Mestizo participant has a 0.37 probability of expressing a personal norm for the corporal punishment of under-performing students, but only a 0.04 probability of guessing that this norm is held by a randomly chosen co-ethnic (compare question 5 on the Mestizo axes in Figure 2a and b). A potential cause of this discrepancy is discussed in Supplementary Appendix B.1.6.

### Cross-cultural competence

In general, Matsigenka tend to make less accurate out-group guesses and more accurate in-group guesses than Mestizos (compare columns 2 and 3 of Figure 3, and see also Supplementary Figures S2, S9, S10 and S13). Thus, by the above definition of cross-cultural competence, it is not possible to make a general claim that one ethnic group is more cross-culturally competent than the other. Figure S9 shows that these results are driven by particularly inaccurate Matsigenka out-group guesses in the domain of healthcare, and particularly inaccurate Mestizo in-group guesses in the domain of education. This finding is discussed in more detail in Supplementary Appendix B.1.6, and its implications for Fiske's (1993) theory of stereotyping and group-level power differences are explored in Supplementary Appendix B.1.7.

**Figure 4.**
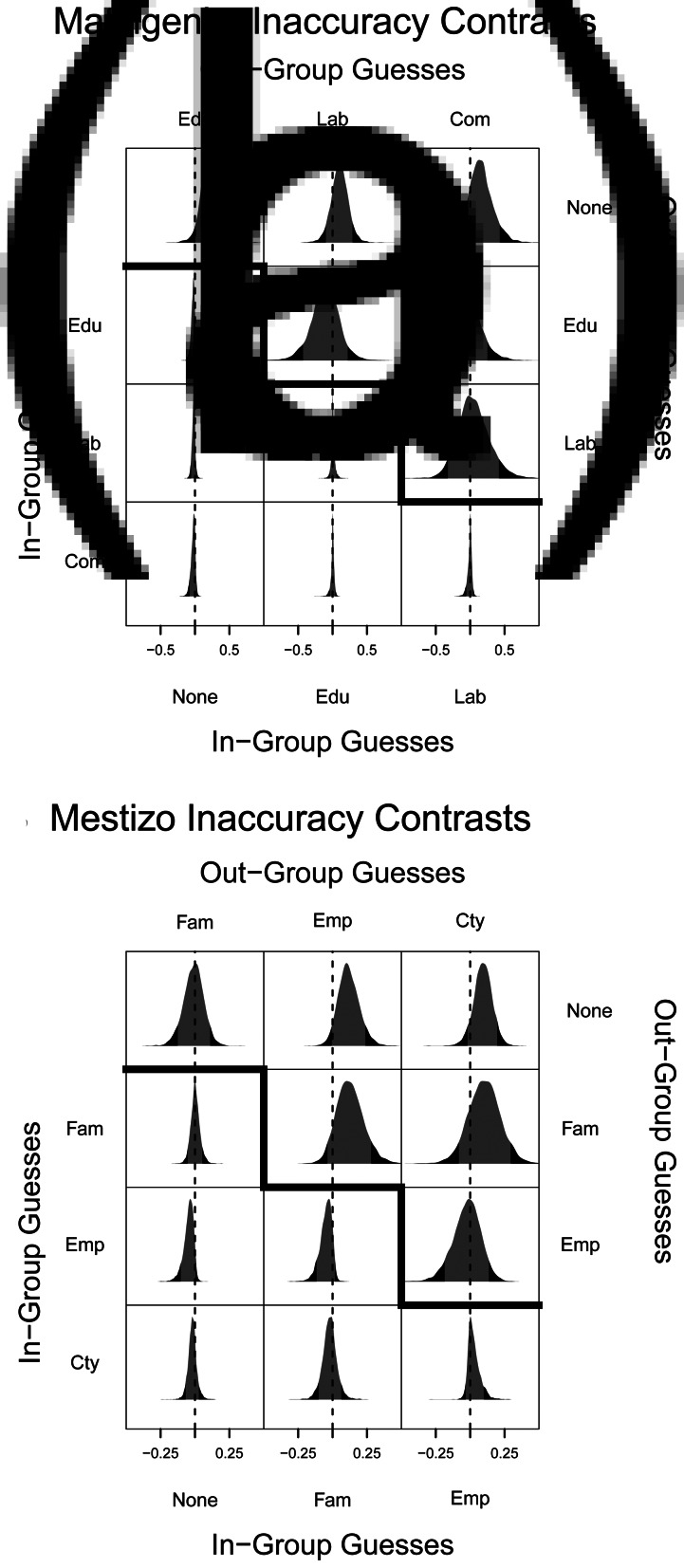
Contrasts (differences) of mean guess inaccuracy. (a) Above diagonal: each cell contains the contrast (row minus column) of the posterior distributions for the mean inaccurary of out-group guesses by counterfactual Matsigenka with each experience type in Figure 3a. Distributions to the right of 0 indicate that the row experience type tended to make more inaccurate guesses than the column experience type. Distributions to the left of 0 indicate the opposite. Distributions around 0 indicate no detectable differences in guess inaccuracy between experience types. (a) Below diagonal: analogous contrasts for Matsigenka mean in-group guess inaccuracies. (b) Analogous contrasts for Mestizo mean out-group (above diagonal) and in-group (below diagonal) guess inaccuracies. The 90% HPDI are shown in grey.

For Matsigenka, Figures 3a and 4a show that education experience with Mestizos, in the absence of and relative to other types of inter-ethnic experience, tends to be associated with: (a) holding cultural norms more similar to those of an average Mestizo; (b) lower inaccuracy when guessing about the norms of Mestizos (particularly compared to no inter-ethnic experience); and (c) comparable inaccuracy when guessing about the norms of co-ethnics (see also Figures S11, S12, S14 and S15). Thus, for Matsigenka, education experience tends to be associated with higher levels of cross-cultural competence. Figure S14 shows that this is due to relatively more accurate guesses about Mestizo norms in the domains of education, healthcare and fairness.

For Mestizos, Figures 3b and 4b show that the experience of living in an indigenous community, in the absence of and relative to other types of inter-ethnic experience, is associated with holding norms more similar to those of an average Matsigenka. This experience, as well as that of employing Matsigenka wage laborers, tends to be associated with: (a) lower inaccuracy when guessing about the norms of Matsigenka, compared with no inter-ethnic experience or indigenous family experience; and (b) comparable inaccuracy when guessing about the norms of co-ethnics (see also Figures S11, S12, S16–S19). Thus, for Mestizos, community experience and employer experience, although each associated with holding different norms, both tend to be associated with higher levels of cross-cultural competence. Figures S16 and S18 show that this is due to relatively more accurate guesses about Matsigenka and Mestizo norms in the domains of education, healthcare, fairness and labor. Additional aspects of these results, including comparisons of variance in responses and implications of ethnic differences in power, are presented and discussed in Supplementary Appendix B.1.

### Ethnographic interpretation

Ethnographic evidence suggests that Matsigenka students acquire certain Mestizo-typical norms while attending Mestizo-run boarding schools, partly as a consequence of their low bargaining power in student–teacher coordination occurring exclusively with Mestizos (Bunce and McElreath 2017). Cross-cultural competence probably results from learning that such norms are common among Mestizos, yet not forgetting that alternative norms are common among their fellow Matsigenka. An analogous process is plausible for Mestizos who live temporarily as minorities in indigenous communities (see Supplementary Appendix B.1.8). In contrast, Mestizos who employ Matsigenka wage laborers (e.g. in agriculture, logging, and tourism) usually also employ at least as many Mestizos for the same jobs. The frequent shortage of labor in this region increases laborers’ bargaining power relative to employers (Bunce and McElreath 2017). Thus, cross-cultural competence of employers (e.g. in domains of labor, fairness and healthcare) facilitates good relations with a wider range of potential laborers, yet there is little incentive for employers to personally adopt Matsigenka-typical norms, as interactions in this domain with their fellow Mestizos are at least as important. Such accommodation of perceived Matsigenka-typical norms is illustrated in the words of a Mestizo who routinely contracted both Matsigenka and Mestizo field hands: ‘[Matsigenka] work well, but I let them work in their own way, because their world is different. A type of person like us [Mestizos] already knows how this kind of work is. [We] work until late, [and are] more demanding. But a Machi [*recte* Matsigenka], when you bring him [to the agricultural field], you let him work in his own way. If he wants to leave, then we leave. If he wants to go for a little while because he is tired, I let him … If I say something to them, like demanding that they do something, they get angry and they leave you, just like that’ (Bunce and McElreath 2017).

## Discussion

As shown, cross-cultural competence may develop in individuals who retain personal norms typical of their co-ethnics, such that knowledge of out-group norms is added in a supplementary capacity to the norms that they personally hold (e.g. Mestizo employers of Matsigenka). For convenience, I refer to this as the ‘supplement’ path to cross-cultural competence. Alternatively, cross-cultural competence may be a side effect of the process of adopting out-group norms, such that these new norms replace in-group-typical norms as those that individuals personally hold (e.g. Matsigenka educated among Mestizos). I refer to this as the ‘replacement’ path. The ethnographic evidence above suggests that the path taken may be a function of the frequency of intra- and inter-ethnic coordination interactions and the balance of bargaining power during such interactions. Other factors may also contribute. For instance, the desire both to maintain an ethnic identity closely linked to a suite of cultural norms, while also engaging with members of an ethnically distinct group, may facilitate the supplement path to cross-cultural competence (e.g. integration of immigrants: Berry 1997). In contrast, if ethnic groups exhibit salient differences in subjectively perceived success or prestige, cultural norms of the more prestigious group may be personally adopted by members of the other, regardless of the frequency of direct inter-group interaction (success-biased inter-ethnic social learning; Bunce and McElreath 2017), facilitating the replacement path.

The two paths to cross-cultural competence suggested here parallel two paths to bilingualism proposed by Lambert (1981): additive bilingualism occurs when two languages learned by an individual have comparable social value and respect. The second language is learned in order to supplement, rather than replace, the first. In contrast, subtractive bilingualism results from social pressure on individuals to reject their native language in favor of a more prestigious second language. Subtractive bilingualism constitutes a transition state from a low- to a high-status language, and, as such, would be expected to result in the eventual loss of language diversity in a population (Kandler 2009).

Multiethnic societies are often characterized by a high prevalence or prominence of cross-culturally competent individuals (Wise and Velayutham 2014). However, the long-term sustainability of extant cultural diversity in such societies seems doubtful if one ethnic group becomes cross-culturally competent as a consequence of adopting personally held out-group norms. Similar to subtractive bilingualism, this replacement path to cross-cultural competence may contribute to the loss of cultural diversity, as it can facilitate the invasion of a group by out-group-typical norms (see Supplementary Appendix B.2). Inversely, the scope for sustainability may be wider if the supplement path is followed, such that cross-cultural competence is achieved while retaining personally held in-group norms.

Supplementary Appendix B.2 outlines theoretical support for this latter prediction. The argument depends on cross-culturally competent individuals receiving a benefit to inter-ethnic coordination interactions that outweighs the benefit to such interactions received by non-cross-culturally competent individuals. Under such conditions, the supplement path to cross-cultural competence could facilitate stable states of a population where cultural norms typical of each group are present. Whether these conditions are sufficient, and relevant in the real world, awaits further theoretical and empirical investigation. However, if shown to be widely applicable across populations, this finding suggests that immigration-induced inter-ethnic engagement following the supplement path to cross-cultural competence may, in theory, result in neither the inevitable loss nor dilution of either host-nation or immigrant cultural norms within their respective communities. Consequently, the two paths to cross-cultural competence suggested here have potentially important implications for our understanding of cultural dynamics in humans, and perhaps other species that use horizontal social learning to acquire group-specific coordination behavior (e.g. *Cebus*: Perry *et al.* 2003).

It is important to emphasize the tentative nature of the above proposals regarding paths for the individual-level development of cross-cultural competence, and the population-level implications of these paths for the long-term sustainability or loss of cultural norms. A rigorous empirical study of individual development or cultural dynamics requires a longitudinal research design, preferably replicated in multiple societies. The primary contribution of the present cross-sectional study is methodological: I present a simple field data collection technique that can be used to construct a quantifiable proxy of two forms of cross-cultural competence, and a statistical method to compare people's interaction experiences on the basis of this measure. These tools facilitate future longitudinal cross-cultural work.

## Research transparency and reproducibility

Data and code in R and Stan to reproduce the analysis in the main text and the Supplementary Materials are available on Github at https://github.com/jabunce/bunce-2018-cross-cultural-competence.

## Supplementary material

For supplementary material accompanying this paper visit http://dx.doi.org/10.1017/ehs.2020.1. click here to view supplementary material

## References

[ref1] Akkerman T and Hagelund A (2007) ‘Women and children first!’: anti-immigration parties and gender in Norway and the Netherlands. Patterns of Prejudice 41, 197–214.

[ref2] Anand R and Lahiri I (2009) Intercultural competence in health care: developing skills for interculturally competent care. In DK Deardorff (ed.), The SAGE Handbook of Intercultural Competence. Los Angeles, CA: SAGE, book section 23, pp. 387–402.

[ref3] Bafumi J, Gelman A, Park DK and Kaplan N (2005) Practical issues in implementing and understanding Bayesian ideal point estimation. Political Analysis 13, 171–187.

[ref4] Barth F (1998) Introduction. In F Barth (ed.), Ethnic Groups and Boundaries: The Social Organization of Culture Difference. Long Grove: Waveland Press, pp. 9–38.

[ref5] Berry JW (1997) Immigration, acculturation, and adaptation. Applied Psychology 46, 5–68.

[ref6] Betz HG and Meret S (2009) Revisiting Lepanto: the political mobilization against Islam in contemporary Western Europe. Patterns of Prejudice 43, 313–334.

[ref7] Bicchieri C (2006) The Grammar of Society: The Nature and Dynamics of Social Norms. Cambridge: Cambridge University Press.

[ref8] Boyd R and Richerson PJ (2009) Voting with your feet: payoff biased migration and the evolution of group beneficial behavior. Journal of Theoretical Biology 257, 331–339. 19135062 10.1016/j.jtbi.2008.12.007

[ref9] Bunce JA and McElreath R (2017) Interethnic interaction, strategic bargaining power, and the dynamics of cultural norms: a field study in an Amazonian population. Human Nature 28, 434–456. 28822079 10.1007/s12110-017-9297-8 PMC5662675

[ref10] Bunce JA and McElreath R (2018) Sustainability of minority culture when inter-ethnic interaction is profitable. Nature Human Behaviour 2, 205–212.

[ref11] Carvalho JP (2017) Coordination and culture. Economic Theory 64, 449–475.

[ref12] Chiu CY, Gelfand MJ, Yamagishi T, Shteynberg G and Wan C (2010) Intersubjective culture: the role of intersubjective perceptions in cross-cultural research. Perspectives on Psychological Science 5, 482–493. 26162194 10.1177/1745691610375562

[ref13] Creanza N, Kolodny O and Feldman MW (2017) Greater than the sum of its parts? Modelling population contact and interaction of cultural repertoires. Journal of the Royal Society Interface 14. 10.1121/1.2934779. PMC5454306 28468920

[ref14] Cronk L (1991) Intention versus behaviour in parental sex preferences among the Mukogodo of Kenya. Journal of Biosocial Science 23, 229–240. 2061350 10.1017/s0021932000019246

[ref15] Erten EY, van den Berg P and Weissing FJ (2018) Acculturation orientations affect the evolution of a multicultural society. Nature Communications 9, 58. 10.1038/s41467-017-02513-0 PMC5754364 29302036

[ref16] Fantini AE (2009) Assessing intercultural competence: issues and tools. In DK Deardorff (ed.), The SAGE Handbook of Intercultural Competence. Los Angeles, CA: SAGE, book section 27, pp. 456–476.

[ref17] Fernando C, Valijärvi RL and Goldstein RA (2010) A model of the mechanisms of language extinction and revitalization strategies to save endangered languages. Human Biology 82, 47–75. 20504171 10.3378/027.082.0104

[ref18] Fiske ST (1993) Controlling other people: the impact of power on stereotyping. American Psychologist 48, 621–628. 8328729 10.1037//0003-066x.48.6.621

[ref19] Foley RA and Lahr MM (2011) The evolution of the diversity of cultures. Philosophical Transactions of the Royal Society of London B 366, 1080–1089. 10.1098/rstb.2010.0370 PMC3049104 21357230

[ref20] Ginges J, Atran S, Medin D and Shikaki K (2007) Sacred bounds on rational resolution of violent political conflict. Proceedings of the National Academy of Sciences of the USA 104, 7357–7360. 17460042 10.1073/pnas.0701768104 PMC1863499

[ref21] Golder M (2016) Far right parties in Europe. Annual Review of Political Science 19, 477–497.

[ref22] Gurven M, Zanolini A and Schniter E (2008) Culture sometimes matters: intra-cultural variation in pro-social behavior among Tsimane Amerindians. Journal of Economic Behavior & Organization 67, 587–607. 19122839 10.1016/j.jebo.2007.09.005 PMC2582818

[ref23] Henrich J and McElreath R (2003) The evolution of cultural evolution. Evolutionary Anthropology 12, 123–135.

[ref24] Hong Yy, Morris MW, Chiu Cy and Benet-Martínez V (2000) Multicultural minds: a dynamic constructivist approach to culture and cognition. American Psychologist 55, 709–720. 10916861 10.1037//0003-066x.55.7.709

[ref25] Jackman S (2001) Multidimensional analysis of roll call data via Bayesian simulation: identification, estimation, inference, and model checking. Political Analysis 9, 227–241.

[ref26] Johnson JP, Lenartowicz T and Apud S (2006) Cross-cultural competence in international business: Toward a definition and a model. Journal of International Business Studies 37, 525–543.

[ref27] Kandler A (2009) Demography and language competition. Human Biology 81, 181–210. 19943743 10.3378/027.081.0305

[ref28] Kandler A, Unger R and Steele J (2010) Language shift, bilingualism and the future of Britain's Celtic languages. Philosophical Transactions of the Royal Society of London B: Biological Sciences 365, 3855–3864. 21041210 10.1098/rstb.2010.0051 PMC2981914

[ref29] Katz D and Allport F (1931) Students’ Attitudes: A Report of the Syracuse University Reaction Study. Syracuse, New York: Craftsman Press.

[ref30] Kopenawa D and Albert B (2013) The Falling Sky: Words of a Yanomami Shaman. Cambridge, MA: The Belknap Press of Harvard University Press.

[ref31] Kullback S and Leibler RA (1951) On information and sufficiency. The Annals of Mathematical Statistics 22, 79–86.

[ref32] Kuran T and Sandholm WH (2008) Cultural integration and its discontents. The Review of Economic Studies 75, 201–228.

[ref33] Labov W, Fisher S, Gylfadottír D, Henderson A and Sneller B (2016) Competing systems in Philadelphia phonology. Language Variation and Change 28, 273–305.

[ref34] Lamana G (2008) Domination Without Dominance: Inca–Spanish Encounters in Early Colonial Peru. Duke, NC, Durham.

[ref35] Lambert WE (1981) Bilingualism and language acquisition. Annals of the New York Academy of Sciences 379, 9–22. 10.1111/j.1749-6632.1981.tb41998.x 6951502

[ref36] Llosa Isenrich E and Nieto Degregori L (2003) El Manu a Través de la Historia. Lima: PRO-MANU.

[ref37] McElreath R (2016) Statistical Rethinking: A Bayesian Course with Examples in R and Stan. Texts in Statistical Science. Boca Raton, FL: CRC Press.

[ref38] Medin D, Ross N, Cox D and Atran S (2007) Why folkbiology matters: resource conflict despite shared goals and knowledge. Human Ecology 35, 315–329.

[ref39] Mesoudi A (2018) Migration, acculturation, and the maintenance of between-group cultural variation. PLoS ONE 13, 1–23. 10.1371/journal.pone.0205573 PMC6191118 30325943

[ref40] Miller DT and McFarland C (1987) Pluralistic ignorance: when similarity is interpreted as dissimilarity. Journal of Personality and Social Psychology 53, 298–305.

[ref41] Mor S, Morris MW and Joh J (2013) Identifying and training adaptive cross-cultural management skills: The crucial role of cultural metacognition. Academy of Management Learning & Education 12, 453–475.

[ref42] Morris MW, yi Hong Y, Yue Chiu C and Liu Z (2015) Normology: integrating insights about social norms to understand cultural dynamics. Organizational Behavior and Human Decision Processes 129, 1–13.

[ref43] Perry S, Baker M, Fedigan L, Gros-Louis J, Jack K, MacKinnon KC, Manson JH, Panger M, Pyle K and Rose L (2003) Social conventions in wild White-faced Capuchin monkeys: evidence for traditions in a Neotropical primate. Current Anthropology 44, 241–268.

[ref44] Prentice DA and Miller DT (1993) Pluralistic ignorance and alcohol use on campus: some consequences of misperceiving the social norm. Journal of Personality and Social Psychology 64, 243–256. 8433272 10.1037//0022-3514.64.2.243

[ref45] R Core Team (2017) R: A Language and Environment for Statistical Computing. Vienna: R Foundation for Statistical Computing.

[ref46] Richerson PJ and Boyd R (2005) Not by Genes Alone: How Culture Transformed Human Evolution. Chicago, IL: University of Chicago Press.

[ref47] Rydgren J (2007) The sociology of the radical right. Annual Review of Sociology 33, 241–262.

[ref48] Sahlins M (1994) Cosmologies of capitalism: the trans-Pacific sector of ‘The World System’. In NB Dirks G Eley and SB Ortner (eds), Culture/Power/History: A Reader in Contemporary Social Theory. Princeton, NJ: Princeton University Press, book section 13, pp. 412–455.

[ref49] Schacht R and Grote M (2015) Partner choice decision making and the integration of multiple cues. Evolution and Human Behavior 36, 456–466.

[ref50] Ségurel L and Bon C (2017) On the evolution of lactase persistence in humans. Annual Review of Genomics and Human Genetics 18, 297–319. 10.1146/annurev-genom-091416-035340 28426286

[ref51] Shepard Glenn HJ, Rummenhoeller K, Ohl J and Yu DW (2010) Trouble in paradise: Indigenous populations, anthropological policies, and biodiversity conservation in Manu National Park, Peru. Journal of Sustainable Forestry 29, 252–301.

[ref52] Spitzberg BH and Changnon G (2009) Conceptualizing intercultural competence. In DK Deardorff (ed.), The SAGE Handbook of Intercultural Competence. Los Angeles, CA: SAGE, book section 1, pp. 2–52.

[ref53] Stan Development Team (2018) *RStan: the R Interface to Stan*.

[ref54] Trapnell LA (2003) Some key issues in intercultural bilingual education teacher training programmes – as seen from a teacher training programme in the Peruvian Amazon basin. Comparative Education 39, 165–183.

[ref55] United Nations General Assembly (2007) United Nations Declaration on the Rights of Indigenous Peoples. A/RES/61/295.

[ref56] van der Linden WJ (2016) Unidimensional logisitic response models. In WJ van der Linden (ed.), Handbook of Item Response Theory: Models. Boca Raton, FL: CRC Press, Volume 1, pp. 13–30.

[ref57] Wise A and Velayutham S (2014) Conviviality in everyday multiculturalism: some brief comparisons between Singapore and Sydney. European Journal of Cultural Studies 17, 406–430.

[ref58] Wolf ER (1982) Europe and the People Without History. Berkeley, CA: University of California Press.

