# Supplementary material for: Field evidence for two paths to cross-cultural competence: implications for cultural dynamics
Source: Evol Hum Sci. 2020 Feb 7;2:e3. doi: 10.1017/ehs.2020.1 (PMC10427313; doi:10.1017/ehs.2020.1)
Supplement: Supplementary file 1 [file S2513843X20000018sup001.pdf]

APPENDICES

Field evidence for two paths to cross-cultural competence: implications for cultural dynamics

John A. Bunce (ORCID 0000-0003-4092-485X)

Department of Human Behavior, Ecology, and Culture, Max Planck Institute for Evolutionary Anthropology, Leipzig, Germany
Department of Anthropology, University of California, Davis, USA

Email: john_bunce@eva.mpg.de

Appendix A. Supplementary Methods
Appendix B. Supplementary Discussion
Appendix C. Supplementary Figures and Tables
Supplementary References

Appendix A. Supplementary Methods

Appendix A.1. Procedure for in-group and out-group guess interviews

I repeated the following procedure for each of the 14 questions: I read one question. The interviewee made in-group and out-group guesses for that question. The interviewee selected one card at random from the in-group stack and one card from the out-group stack. I compared the interviewee's in-group guess to the Personal Norm response for that question written on the selected in-group card, and the same for the out-group guess relative to the response on the out-group card. I informed the interviewee whether she had won money for her in-group and out-group guesses. I then read the next question and repeated the procedure. Interviewee winnings were tallied at the end of the 14 questions and dispensed in cash. This methodology bears some similarities to that employed by Gurven et al. (2008), Ross et al. (2007), and Medin et al. (2007). Responses recorded during this round of interviews are referred to as in-group and out-group guesses, and are distinguished from the personally-held (ego) norms recorded during the Personal Norm interview, above.

Further description of data collection methodology, as well as translations of vignette questions are provided in Section Appendix A.2.

Appendix A.2. Vignette question development

In Tayakome and Atalaya (Figure S1), I lived with a host family, routinely participating in household and community activities and sharing meals. In Boca Manu, I lived in a rented room, ate at local restaurants, and participated in activities such as boat building and wood extraction at the invitation of residents. In all three communities I conducted several rounds of individual semi-structured and structured interviews with adult residents. All interviews were conducted by me in the interviewee's preferred language (Matsigenka or Spanish) while out of earshot of other community members. I conducted at least one interview with all but two residents of Tayakome, and with approximately two thirds of the residents of Boca Manu and Atalaya. Included in this study are interviews with members of two of the three Matsigenka families living in Boca Manu, two Matsigenka residents of Atalaya, as well as one Mestizo technician working in the health post in Tayakome. The first rounds of interviews entailed soliciting a life-history narrative. From these interviews, I identified education, labor, commerce, domestic relations,

and community relations as domains of common Matsigenka-Mestizo inter-ethnic interaction. In follow-up interviews I recorded each individual's recollection of personal intra- and inter-ethnic experience in these domains. Based on my own experience living in these communities, I then designed a set of vignette questions (Bernard, 2006), fourteen of which are presented below. From the perspective of this study, none of the vignette questions has a correct or incorrect answer, so no value is assigned to affirmative versus negative responses to any question. In cases of individuals who are bi-culturally competent, I assume that these vignette questions measure an individual's favored norms. Thus, during some inter-ethnic interactions, such an individual may actually coordinate using non-favored norms which do not coincide with her answers to the vignette questions (e.g., Mestizos hiring Matsigenka wage laborers).

Table 1 presents the fourteen vignette questions employed in this study, across eight social coordination contexts. I developed questions in Spanish with the help of an English-Spanish bilingual Peruvian collaborator. I translated the questions into Matsigenka, and then had them back-translated from Matsigenka to Spanish with the help of a Matsigenka-Spanish bilingual Matsigenka collaborator in Tayakome (translations below). Due to organizational difficulties during the Personal Norm Interview, questions were presented in the same order to interviewees from Tayakome and Boca Manu, but the order of questions 13 and 14 changed for interviewees in Atalaya. Questions were presented in the same order to all interviewees in all communities during the in-group and out-group guess interview.

The number of questions per social context is not balanced, i.e., there are two questions for some contexts, while other contexts have only one question. Only a subset of the questions asked during Personal Norm Interviews was also asked during the guess interviews. The particular questions chosen for the guess interviews represent a balance between covering all social coordination contexts of interest and choosing questions that had (according to my impressions) been easiest for participants to understand and would likely pose fewer problems of interpretation in the context of the novel guessing interview. Only questions that were asked in both Personal Norm Interviews and Guess Interviews are presented and analyzed here.

During the Personal Norm Interviews, interviewees responded affirmatively or negatively (or, for some questions, selected one of the answer options) after I initially presented each vignette question. For a subset of interviewees, I then asked them why they responded the way they did. Upon

hearing my request for an explanation, occasionally they then changed their answer from negative to affirmative, or vice versa. If the interviewee did not wish to explain why they changed their initial answer, I did not include their response to that particular question in the present analysis. I exclude these responses in case my request to explain their initial answer led them to believe that I was not in agreement with their answer, which they then changed in order to answer in the way they believed I wanted them to. This occurred despite assurances that their answers were confidential and that I believed there were no right or wrong answers to the vignette questions. These cases of response changes may be due to the fact that interviewees were unaccustomed to answering such unusual questions in this unfamiliar format. On other occasions, some interviewees explained that their response (affirmative or negative) to a vignette question was contingent on additional information that I had not provided in the vignette itself. For example, for Question 1 (below), an informant responded that it is okay for a wife to go hunting sometimes while her husband stays home, but it is not okay if she does it often. Such responses resulted from interviewees thinking more deeply about a vignette than I had when I designed it. I excluded these contingent responses from the present analysis, because I had not provided the requested details (e.g., frequency of the behavior in question) to the rest of the interviewees. For all those interviewees who answered the question without requesting additional details, I assume that they interpreted the question in a similar way (e.g., the wife always goes hunting, not just sometimes). As a result of these two classes of interviewer-interviewee misunderstanding, I excluded a total 155 out of 2310 Personal Norm responses (6.7%). To check the effect of excluding these data, I included people's initial responses, and included contingent responses after providing the additional requested information such that these interviewees interpreted the questions in the way I assumed all other interviewees had. Including or excluding these data has no qualitative effect on the conclusions. For analysis of the in-group and out-group guess questions, only one interviewee's guesses for one question were excluded, as this individual guessed that the majority of people in both the in-group and the out-group had rejected the binary answer choice offered to them for this question and had instead given an alternative answer.

Abbreviations of the vignette questions, corresponding to the figures, are in bold after the question numbers, below. The social context of each question (education, wage labor, commerce, spousal relations, parent-offspring relations, inheritance, fairness, healthcare, and religion) is included in paren-

theses. The response coded as positive (1) on the ego axis (see Appendix A.3.2, below) is indicated below the Matsigenka translation. Questions that were coded differently for the in-group and out-group axes are indicated. My rational for designing each vignette, my expectations for Personal Norm responses, and examples of people's actual responses are provided below.

Question 1, reverse gender (Spousal Relations)

There is a married couple with no children. The woman hunts and fishes (Mestizo: has a job and makes more money). The man stays home and cooks, weaves (Mestizo: cleans), and washes clothes. Is this okay or not okay?

Hay una pareja sin hijos. La mujer trabaja y gana plata. El hombre se queda en la casa, cocina, limpia, y lava ropa. ¿Está bien?

Ainho ogari tsinane, mameri otomi, mameri oshinto. Iroro ogotake omatsigatake ochacopi cameti. Onti kovinsari. Iroro oati onkogira ivatsa inkenishkue. Yogari ojina ainho iripitake ivanko, yonkotia sekatsi, intiri ijamatia, ikiviasarati igamisate. Cameti o tera cameti?

A response of "Okay" was coded as positive. This question is coded in the same way for the in-group and out-group latent axes. Differences between questions asked of Matsigenka and Mestizo interviewees reflect the different tasks stereotypical of each gender in the two ethnic groups. This illustrates a norm for flexibility in gendered labor roles.

This question was inspired by my observation of the salient division of labor by sex in many domains of Matsigenka life in Tayakome, e.g., men hunt, build houses, and clear swidden fields, while women cook, weave, and sweep the home area. However, I also observed many activities in which men and women often participate together, e.g., fishing, making roof thatching, planting and weeding fields. Occasionally I observed some men helping with "women's work", e.g., peeling manioc, and some women doing "men's work", e.g., going into the forest alone in search of meat. Though people would laugh about such behavior being unusual, there seemed to be little negative stigma associated with it. In Boca Manu and Atalaya, I observed more overlap in men's and women's labor. For instance, both made wooden boats, tended stores, served food, drove boats, and worked in banana fields. However, despite this overlap, my impression was that there was considerably more

machismo in the Mestizo towns, i.e., more value was placed on men and the labor that they performed. Among the Matsigenka, my impression was that, despite a stricter division of labor, the activities performed by each sex were valued to a similar degree. I did not have a strong a priori hypothesis about how Matigenka and Mestizos would answer this question.

A large majority (74%) of Matsigenka answered "Okay", while only about half (53%) of Mestizos did (Figure 2A). Most Matsigenka thought this question was highly unusual, and an example of a typical initial response is, "Women don't go to the forest . . . A woman stays in the house and cooks manioc", which reflects the behavior actually performed by most Matsigenka women. However, when I asked if it would be okay if a woman wanted to engage in male activities, an example of a typical response is, "[Women performing those activities] is okay sometimes [i.e., in some cases]. If she has no sons and no daughters, it's okay if she goes to hunt. [The man says to her] go hunt spider monkey. I'll cook manioc, I'll wash your clothes." An example of a typical Mestizo response is, "[Reversal of gender roles] is okay because work must be shared. Just because they're men doesn't mean that they aren't going to wash, or if they're women they won't do other things. Rather, the work must be shared." However, an example of another common Mestizo response is, "In my way of thinking, the one who should work is the man. He should support the house, and the woman should take care of the house. Or also the woman could help him with work, but both together should share responsibilities. [In the question, the portrayed activities by gender] should be reversed."

Question 2, daughter babysits (Parent-Offspring Relations)

After school, a ten-year-old daughter cannot go to friend's house to play because she has to care for her two-year-old brother until their parents come home at night. Is this okay?

Después de salir de la escuela en la tarde, una hija de diez años no puede ir a jugar con sus amigas porque tiene que volver a la casa y cuidar a su hermano menor de dos años hasta que lleguen sus papás en la noche. ¿Está bien?

Oga oshinto oshiriagakota diez años. Hatanai chavini, okonteiganae escuela. Hanki agavea omagempitira. Ogari oatae ovankokue ogierira iariri itiomiani ishiriagakota dos años, iripigaira iriegi tsitenigeti. Cameti o tera

cameti?

A response of “Okay” was coded as positive. This question is coded in the same way for the in-group and out-group latent axes. This illustrates a norm that older children share responsibility for care of their siblings.

This question was inspired by my observation that Matsigenka children, especially girls, are given considerable responsibility for tending to their younger siblings. Girls as young as six or seven are already adept at carrying and entertaining infants. I did not observe Mestizo children being given so much responsibility. I hypothesized that Matsigenka interviewees would answer “Okay” and Mestizos would not.

Nearly all (96%) Matsigenka answered “Okay”, and a slight majority (62%) of Mestizos did as well (Figure 2A). Most Matsigenka responded with a decisive “Okay”, as if the answer was obvious. In contrast, an example of a common Mestizo response is, “You know that all children have the right to a little bit of freedom to have fun. It’s a child and children tend to play a lot. And it’s too much responsibility for a girl of that age to take care of a child.”

Question 3, not wear dead hat (Inheritance)

A man always wears his favorite hat. After he dies, his son takes the hat and wears it. When he wears it he remembers his father. Is this okay?

Un hombre siempre lleva un gorro favorito. Se muere. El hijo coge el gorro y se lo pone. Cuando se lo pone, piensa en su papá. ¿Está bien?

Yogari sidadi kantani yamea igorate. Imbogini ikamake. Itomi yagakero igorate irashi iriri ikamake. Itomi kantani igoraterora, kantani ikenkirira iriri. Cameti o tera cameti?

A response of “Not okay” was coded as positive. This question is coded in the same way for the in-group and out-group latent axes. This illustrates a norm for the transfer of goods with sentimental value from the deceased to the living.

This question was inspired by my observation, during a Matsigenka funeral, that nearly all of the (few) possessions of the deceased that had been used (e.g., clothes, pots, sleeping mat, arrows) are either destroyed or buried

with the person. Results of my conversations with people coincide with the interpretation of Shepard (2002), that spirits of the deceased return to look for their possessions and companionship, causing sickness in the living. I did not observe such concern about the belongings or dangerous spirits of the deceased while attending a Christian Mestizo wake, though I was not as close to this family and so I did not learn what happened to the belongings of the deceased. I hypothesized that Matsigenka would answer ”Not okay” and Mestizos would answer ”Okay”.

About half (47%) of Matsigenka answered “Not okay”, while a large majority (81%) of Mestizos answered “Okay” (Figure 2A). An example of a typical Matsigenka explanation is, “The old man dies, he takes his hat with him, and wears it. The son says, ‘That hat, I'll leave it alone. It's yours, a Viracocha [Mestizo] gave it to you. Now I'll leave it alone there. I won't take it.’ [The dead father] takes it with him . . . [If you take your dead father's hat] you will remember your father and you will get sick.” An example of a typical Mestizo response is, “Some who want to be close to their most beloved [deceased] person take it [i.e., the hat] . . . But to be in that moment with all those memories also brings other bad things, heart problems, anxiety, to be thinking that your mother is watching you, or is here. It brings with it many consequences. But I would do it [i.e., take the hat]. In my opinion, I would do it.”

Question 4, wife drinks alone (Spousal Relations)

A woman wants to drink alcohol, but her husband doesn't want to drink. So the wife goes to drink without her husband. Is this okay?

Una mujer quiere tomar, pero su esposo no quiere tomar. Así que ella va sola a tomar. ¿Está bien?

Oga tsinane okogake ovikempara owidoki. Yogari ojina tenka inkogira irovikemprara owidoki. Paniro oatake ovikempara ijina owidoki aiñoni. Cameti o tera cameti?

A response of “Okay” was coded as positive. This question is coded in the same way for the in-group and out-group latent axes. This illustrates a norm for the proper behavior of married women with respect to alcohol.

This question was inspired by my observation that there is, in general, no

stigma against Matsigenka women drinking and getting drunk with fermented manioc beer in Tayakome. This is the only readily available alcohol in the community. It is made in great quantities, and, as interpreted by Shepard (2002) and confirmed by my own observations, is vital to Matsigenka social life, identity, and emotional equilibrium. In my experience, it is not very common for a Matsigenka woman to go to a drinking party without her husband, but it does indeed occur, and seems to lack any negative stigma. In contrast, rarely have I observed Mestizo women drink beer (by far the most common type of alcohol in these Mestizo communities), with or without their husbands, and I have never seen them drunk. In contrast, for most Mestizo men (excepting some evangelical Christians), drinking serves an important social function to establish and maintain friendships, and several Mestizo men in the communities are drunk quite often, in my experience. My impression is that there is a belief among Mestizos that drinking is a male activity and is not appropriate behavior for a woman. I hypothesized that Matsigenka would respond "Okay", and Mestizos would respond "Not okay".

The vast majority (84%) of Matsigenka responded "Okay", while a slight majority (54%) of Mestizos responded "Okay" (Figure 2A). An example of a typical Matsigenka response is, "My wife wants to go drink manioc beer, I say, 'Go, drink manioc beer. I don't want to go. Bring some manioc beer back for me, I'll drink it here.' That is good." An example of a common Mestizo response is, "[A wife drinking along] is bad. How am I going to go have fun knowing that my husband doesn't have time to go with me? Then (and this occurs often around here), that brings gossip. We call the husband [who stays at home] a 'long coat', and things like that. Also, one must take care of the couple's image [i.e., reputation] . . . [the wife can drink] maybe with her husband. As a couple there is more protection."

Question 5, teacher hits (Education)

A teacher hits students when they don't learn. Is this okay?

Un profesor golpea a sus alumnos cuando no aprenden. ¿Está bien?

Yogotagantasirira ipasatakeri estudiante tera irogoigia sankevantera. Cameti o tera cameti?

A response of "Okay" was coded as positive. This question is coded in

the same way for the in-group and out-group latent axes. This illustrates a norm for the appropriate behavior of teachers with respect to students.

This question is inspired by my observation that, though rare among the Matsigenka families that I lived with, corporal punishment of children was occasionally deemed both acceptable and necessary. This usually entailed spanking or hitting with the stem of a stinging nettle plant (*Urtica* sp.) which causes intense but transient discomfort. Causes included disobedience, or neglect of a younger sibling under the child's care. I never observed or heard about either of the two Matsigenka primary school teachers in Tayakome hitting students, except for one instance of a particularly disobedient older student, who was hit after school in the presence, and with the approval, of the child's parents. I did not observe corporal punishment of children by their parents in the Mestizo towns, though beating of women by their male partners is a serious problem. I did observe corporal punishment of Mestizo primary school children by their teacher. Students formed a line, and the teacher asked each in turn to answer a math problem learned the previous day. Those answering incorrectly received a slap on the palm with a stick. The slaps were not delivered with much force, and I did not observe any of the struck students to be in obvious discomfort afterward. I did not have a strong a priori hypothesis about how Matsigenka and Mestizos would answer this question.

More than half of the Matsigenka (60%) responded "Okay", while less than half of Mestizos (37%) responded "Okay" (Figure 2A). An example of a common Matsigenka response was, "It is okay to hit him [i.e., the student who doesn't learn]. He'll learn faster." An example of a common Mestizo answer was, "It's not okay [to hit students], because one must teach children . . . Some children are best treated with tenderness, but others are best treated strictly. But this doesn't mean hitting them. Rather, constant communication . . . It's best [if the teacher] talks with the parents, saying, 'Your son isn't doing his homework.' Then, between father and son they settle the score in their house because the son didn't do his homework."

Question 6, no questions (Education)

A student pays attention to the teacher and never asks any questions. Is this okay?

Un estudiante siempre escucha y hace caso al profesor. Nunca hace

ninguna pregunta. ¿Está bien?

Yogari estudiante kantani ikemisantake yogatagantasirira. Tenka inkogakote. Cameti o tera cameti?

A response of “Okay” was coded as positive. This question is coded in the same way for the in-group and out-group latent axes. This illustrates a norm for the appropriate behavior of students in the classroom.

This question is inspired by my experience teaching English to Matsigenka and Mestizo primary school students at the request of their teachers. In Tayakome, my wife (a fellow anthropologist conducting research in Tayakome independent of this study) and I taught every weekend for a year in the schoolhouse. In the Mestizo communities I taught kindergarten, primary school, and secondary school students, during one school-day. Matsigenka students listened attentively, copied assiduously from the chalkboard, and performed the tasks given to them. They did not ask questions, and most attempted to avoid answering them. The general form of this behavior persisted over the entire year, despite the fact that I taught them in their language, knew them and their families quite well, and had a much more relaxed and egalitarian relationship with them outside of school. Their behavior during class coincided with my observation of their behavior during the normal school day with their regular Matsigenka teachers, during learning interactions with other adults outside of school (e.g., learning to weave or make arrows), and also appears very similar to student school-room behavior described by Aikman (2003) for Harakmbut children in another indigenous community in this region of Peru. In contrast, teaching Mestizo students (of all ages) was a constant negotiation. Students were not shy about asking questions, nor answering them. I felt I needed to convince them to do assigned tasks, and talk continuously and authoritatively in order to maintain their attention. Students' behavior appeared similar when I observed them in class with their normal Mestizo teachers. I hypothesized that Matsigenka would respond “Okay”, and Mestizos would respond “Not Okay”.

A majority (65%) of Matsigenka responded “Okay”, while a majority (85%) of Mestizos responded “Not okay” (Figure 2A). An example of a typical Matsigenka response is, “He [i.e., the student] pays attention, writes, and reads the book. That's good.” Such behavior was sufficient, and questioning on the part of the student was not necessary. However, when I asked if it was okay if a student also asked questions, this interviewee stated, ”[If] he asks

questions, that's good [too]." An example of a typical Mestizo response was, "[Not asking questions] is not good. The student must always be a person who is listening, and he must be asking whether he agrees with what is said or not. For this, the child should be prepared. One must awaken the interest of children . . . [If the student doesn't ask questions then] that is being a conformist. Up to a certain point and no more they close off from what they have learned. But the child must [rather] be open to the world."

Question 7, post flu (Healthcare)

If you get a respiratory illness (influenza), do you first go to the health post, first use home remedies, or first go to a shaman or curandero?

¿Si tiene gripe, Ud. va primero a la posta, primero usa remedios caseros, o primero va al curandero?

Agakempi merentsi. Okityo piatake postakue? Okityo povetsike inchashi pankotsikue? Okityo piatake irishipokempira? Okityo piatake iritasonkempira?

A response of "Health post" was coded as positive. All other responses were coded as negative. This question is coded in the same way for the in-group and out-group latent axes. This illustrates a norm for the appropriate treatment of respiratory illnesses.

This question is inspired by my observation that respiratory infections occur frequently among the Matsigenka of Tayakome (especially during the wet season) and are a common reason that people visit the health post in the community. I had less opportunity to observe Mestizos' behavior when ill. My impression is that respiratory infections tend to be both less common and less serious among Mestizos than among Matsigenka. I had no strong a priori hypothesis about how Matsigenka and Mestizos would answer this question.

A majority (73%) of Matsigenka responded "Health post", while a majority (79%) of Mestizos responded "Home remedies" (Figure 2A). An example of a typical Matsigenka response is, "First I go to the post." An example of a typical Mestizo response is, "[When I get a respiratory illness I stay] just in my house. I grab a pill, or a hot tea, or I dress warmly, or [I use] some cream like Vics Vapor-rub. That's it."

Question 8, not post chest (Healthcare)

You have chest pain (Mestizos: You wake up one day with chest pain). Do you first go to the health post, first use home remedies, or first go to a shaman or curandero?

Algún día Ud. se despierte y le duele el pecho. ¿Ud. va primero a la posta, primero usa remedios caseros, o primero va al curandero?

Pikatsinegetira. Okityo piatake postakue? Okityo piatake irishipokempira? Okityo piatake iritasonkempira? Okityo povetsike inchashi pankotsikue?

A response of "Health post" was coded as negative. All other responses were coded as positive. This question is coded in the same way for the in-group latent axes. However, for the out-group latent axis, this coding was reversed: "Health post" was coded as positive and all other responses were coded as negative. This illustrates a norm for the appropriate treatment of chest pain.

This question is inspired by my observation that chest pain is believed by many Matsigenka of Tayakome to be a classic symptom of attack by a witch (see also Shepard (2002)). According to my conversations with Mestizo technicians in the community health post, heart disease appears to be extremely rare among the Matsigenka. For many Matsigenka, illness resulting from witch attack can be cured (or reversed, inflicted back on the witch) with the help of: 1) medicinal plants; 2) a curer, irishipokempira, who uses steam to remove objects (e.g., spines, string) inserted into the victim's body by the witch; or 3) another type of curer, iritasokempira, who sucks the object out of the body. In conversations with people undergoing these treatments, my impression is that Western medicine is believed to be ineffective against witchcraft. This is consistent with the disconnect between Western indicators of physical health and Matsigenka impressions of well-being in the face of sorcery, observed by Izquierdo (2005). In the Mestizo communities, chest pain is much more salient as an indicator of a serious heart problem, requiring Western medical attention. I suspect that rates of heart-related medical problems are considerably higher in Mestizo communities, as many interviewees told me of conditions, such as type 2 diabetes, that tend to

covary with heart disease in Western societies. I hypothesized that Matsigenka would give a response other than "Health post", while Mestizos would respond "Health post".

Less than half (47%) of Matsigenka and more than half (65%) of Mestizos responded "Health post" (Figure 2A, note inverse coding). An example of a typical Matsigenka response is, "You have chest pain in your heart, you want to go to the health post. [The technician] gives you pills [but] it [i.e, the illness] doesn't get better. Then you go get herbs. There is heart-pain-herb. You mash it up with a rock: ping ping ping. Then you drink it. In the afternoon you feel a little better . . . [if] you drink it and the illness doesn't get better, you go to the healer. He blows on you. [He asks,] 'What hurts you?' He visits you [and he asks] 'What is inside you? A palm-wood splinter? A stone?' . . . He is able to take it out of you, so that you can heal." An example of a typical Mestizo response is, "If I wake up with a painful heart, it's because I have thought a lot, and there is something wrong with that organ. Well, I'd have to go to the doctor. Because a shaman would lead me in the wrong direction [i.e., not cure me]."

Question 9, not pot each (Fairness)

An old woman has two new pots and two adult daughters. One daughter has her own two pots, but wants her mothers pots. The other daughter has no pots, and also wants her mothers pots. When the mother dies, who should inherit the pots? (Illustrated with a diagram. Possible responses: one pot to each daughter; both pots to the daughter who has none)

Hay una mujer vieja con dos ollas nuevas. Tiene dos hijas adultas. Una hija tiene sus propias dos ollas, pero quiere las ollas de su mamá. La otra hija no tiene ollas. También quiere las ollas de su mamá. Cuando la mamá se muere, ¿a quién debería heredarle las dos ollas?

Ogari tsinane okamake. Aityo pitieti ojiromanga otierira. Ainho piteni oshinto antaroni. Paniro oshinto aityo pitieti ojiromangane ashi iroro. Okogake oka otierira jiromanga. Ogari apiteni oshinto, mameri ojiromangane. Ariompa okogake oka otierira jiromanga. Tyani gakerone otierira jiromanga ashi iniro?

A response of "Both pots to the daughter who has none" was coded as

positive. This question is coded in the same way for the in-group and out-group latent axes. This illustrates a norm for fair division according to right versus according to need.

This question was inspired by my life history interviews with Mestizos, in which several people recounted instances of tension between siblings over the division of wealth belonging to a recently-deceased parent. In addition to norms of inheritance, this question is designed to investigate norms of fairness, e.g., division according to entitlement (equal shares to both daughters) or division according to need (both shares to the daughter who has less). My impression was that Mestizos tended to emphasize entitlement, and Matsigenka tended to emphasize need. Thus I hypothesized that Mestizos would give the negative response and Matsigenka would give the positive response. The qualification that the mother's pots are new (i.e., unused) when she dies is to circumvent the Matsigenka-typical norm of destroying or burying the used belongings of the deceased in order to avoid attracting a dangerous dead spirit (see Question 3). I hypothesized that Matsigenka would respond "Both pots to the daughter who has none", while Mestizos would respond "One pot to each daughter."

A large majority (75%) of Matsigenka responded "Both pots to the daughter who has none", while most (68%) Mestizos responded "One pot to each daughter." (Figure 2A). An example of a typical Matsigenka explanation for giving both pots to the daughter who has none is because, "the other one already has pots." An example of a typical Mestizo response is, "I would give one [pot] to each one [i.e., daughter], because the first two pots [of the daughter who already has two] are her own. But I as a mother want to give the inheritance. And as I have two pots, and my other daughter has none, then I should give to each one [i.e., daughter] so that they don't fight. But I can't say to the other daughter [who already has two pots], 'Give her [i.e., your sister] the two pots', because [she] bought [her own two pots] with her own money. So I just give one [to each] in order to avoid [fights]. And with the disadvantage that one [daughter] has three [pots] and the other one."

Question 10, good non-baptized not heaven (Religion)

A good person does not want to be baptized. Where does his or her soul go when they die? Possible responses: up (heaven); down (hell); somewhere else.

Una persona es buena, pero no quiere bautizarse. Cuando se muere, ¿adónde va su alma?

Yogari sidadi inti cameti. Tenka ikogira ibautizatakeri Padre. Imbo ikamake. Tyara iriatae isire? Enokue? Saviakue? Pashini?

A response of "Up" or "Heaven" was coded as negative. All other responses were coded as positive. This question is coded in the same way for the in-group and out-group latent axes. Thus illustrates a norm for the appropriate way to obtain a reward in the afterlife.

For some Matsigenka, there is a distinction between a soul going into the ground (kipatsikue) and going to a place with flames below the world (morekakue)(see also Rosengren (2004)). Both of these places can be indicated by the preposition down (saviakue), but they are not necessarily equivalent in terms of favorable or unfavorable outcomes after death. I realized this after most of the interviews were completed. Therefore, a response of "down" to this question by Matsigenka may be interpreted as "anywhere other than a heaven-like place above the world".

This question was inspired by my interest in how much importance the Matsigenka place on foreign religious (e.g., Catholic) rituals. A Catholic priest ministers to the Matsigenka communities inside Manu National Park, as well as to the Mestizo town of Boca Manu. He usually visits each community twice per year. I was present for one of his visits to Tayakome, during which he said mass (in Spanish), baptized all available children, and distributed gifts (clothes, soap, candy) to all attendees. I had the impression that many attendees came simply to observe the spectacle of the mass, the baptisms, and the gifts, and did not necessarily share the priest's interpretation that baptism removes the "original sin" of their children. However, most did seem to desire that their children be baptized. This question was designed to explore such subjective impressions. Evangelical Christian proselytizing is not permitted within Manu National Park by the park administration. However, two such groups are active in Boca Manu, and provide weekly services. I did not observe religious services of any variety in Atalaya, though residents self-identifying as Catholic or Evangelical Christian were present in the town. My impression is that residents generally travel 30min by road to the larger towns of Pilcopata or Salvación if they seek religious services.

I interpret a response of "Up"/"Heaven" as suggesting a belief that individual autonomous actions are more important in determining supernatural

benefits than is formal acceptance by a representative of an organized religious community. I had no strong a priori hypotheses about how Matsigenka and Mestizos would answer this question.

A slight majority (56%) of Matsigenka responded that the person would not go to Heaven, while a large majority (70%) of Mestizos responded that the person would go to Heaven (Figure 2A). An example of a typical Matsigenka explanation of the possible destinations of the soul after death is, “Up is good. It’s like here [i.e., the earth]. You’re an angry person, you die, the fire burns you in morekari, down.” This interviewee stated that the soul of a good non-baptized person goes “down”. When I then asked about the soul of a bad person who is baptized, this interviewee responded that their soul goes “up”. Thus, it appears that, for this interviewee, baptism is more important than behavior in determining the destination of the soul after death. An example of a typical Mestizo response is, “That’s a problem. Where would it [i.e., the soul of a good non-baptized person] go? Hell? I don’t think so. If he is not baptized, he could go to Heaven in the end, because he was not a bad person on earth. So he goes directly to Heaven, even if he was not baptized.” When I asked about the soul of a baptized child who grows up to be a bad person, this interviewee responded, “To Hell. It doesn’t matter if he is baptized or not baptized, because he is very bad.” Thus it appears that, behavior is more important than baptism in determining the destination of the soul after death.

Question 11, postpone work visit (Wage Labor)

A man is hired to prepare an agricultural field. He stops work at noon in order to go visit a friend. He returns the next day to finish the job. Is this okay?

Un hombre está contratado limpiar una chacra. Deja de trabajar a mediodía porque quiere visitar a su amigo. El día siguiente termina de limpiar la chacra. ¿Está bien?

Kamatitya, ipuinatakeri koriki yogari sidadi itsamaitera imagashipogote irashi Viracocha. Katinga poriatsiri, yoga sidadi yapakuianae itsamaitera, ikogake ikamosote ishaninka. Paita onkuita yagatake itsamaitera, itsongatero imagashipogote irashi Viracocha. Cameti o tera cameti?

A response of “Okay” was coded as positive. This question is coded in the same way for the in-group and out-group latent axes. This illustrates a norm for prioritizing social relations over labor contracts.

This question was inspired by my interviews with Mestizo employers of Matsigenka wage laborers in plantain fields in Atalaya, and my experience working with Matsigenka families in their own manioc fields in Tayakome. Some Mestizo employers complained to me that Matsigenka laborers were not diligent workers (see examples in Bunce and McElreath (2017)). Working alongside Matsigenka and Mestizo wage laborers for a day in both Atalaya and Boca Manu, I did not observe ethnic differences in diligence, though my sample size was small. In Tayakome, social obligations can sometimes take priority over a day of weeding a manioc field, but, in my observation, never to the point of jeopardizing the productivity of a field. I hypothesized that Mestizos would respond “Not okay”, but I did not have a strong a priori hypothesis about how Matsigenka would answer.

A large majority of both Matsigenka (94%) and Mestizos (74%) responded “Okay” (Figure 2A). Most Matsigenka answered this question with a decisive “Okay”, as if the answer was obvious. An example of a typical Mestizo response is, “He can do it [i.e., stop work to visit a friend], because there was no time limit [in the contract] for when he should finish the field.”

Question 12, cheap store (Commerce)

There are two stores. One is cheap with a mean owner. The other is expensive with a nice owner. Where would you buy? (Illustrated with a diagram for Matsigenka.)

Hay dos tiendas. Una tienda es barata pero el dueño es malo. La otra tienda es cara pero el dueño es bueno. ¿Dónde iría Ud. para comprar?

Aityo pitieti otiendate Viracocha. Patiro otiendate tenka opuinatasanotempa. Yogari shintarorira inti kisantari. Apiteni otiendate onti puinatasanori. Yoga shintarorira tenka irikisante. Tyaka picompratake? Hanta puinatasanori irashi cameti shintarorira o apiteni tenka opuinatasanotempa irashi kisantari?

A response of “Cheap store with mean owner” was coded as positive for the ego latent axis. However, for both the in-group and out-group latent axes, the response of “Expensive store with nice owner” was coded as positive. This

contraposes a norm of frugality with a norm of courtesy.

This question was inspired by my observation that Mestizos tended to have strong norms of personal respect in the domain of commerce (e.g., courtesy and formality, unless among very close friends), yet also tended to be concerned with frugality and not being cheated. Matsigenka tend to have smaller amounts of money, which they are usually anxious to convert into as much Western merchandise as possible, yet they also tend to avoid displays of anger (see also Shepard (2002)). I had no strong a priori hypotheses about how Matsigenka or Mestizos would answer this question.

A majority (62%) of Matsigenka and less than half (46%) of Mestizos responded "Cheap store with mean owner" (Figure 2A). An example of a typical Matsigenka explanation for buying in the cheap store with a mean owner is, "The merchandise costs little. If he [the owner] gets angry at me, I'll just buy a little." An example of a typical Mestizo explanation for buying in the expensive store with the nice owner is, "It doesn't matter if the product costs a little more. I go to the store that has an owner who is more friendly, more welcoming, more open. That's where I go. It doesn't matter if the price costs a little more."

Question 13, daughter must marry (Parent-Offspring Relations)

Parents want their daughter to marry a certain man that she does not like. She wants to marry someone else. Should she obey her parents and marry him anyway or not? (Matsigenka: she does not obey her parents, and instead marries the man she wants. Is this okay?)

Los papás quieren que su hija se case con un chico. Pero a la hija no le gusta el chico. Ella quiere otro. ¿Debería hacerles caso a sus papás y casarse con ese chico, o no?

Iriegi ikogaigake ojinantempera irishinto antaroni. Ikantakero "Noshinto, gaeri yogari sidadi, inti cameti." Kantangicha, ishinto tenka okogiri yogari sidadi, okogakeri pashini. Iroventi, iroro, irishinto, tenka okematsataeri iri. Tenka ojinantaigiri yoga sidadi. Agakeri pashini. Cameti o tera cameti?

A response of "She must obey her parents and marry the man she does not like" (Matsigenka: "Not okay") was coded as positive for the ego and in-group latent axes. However, for the out-group latent axis, the response of "She

must not obey her parents. She should marry who she wants" (Matsigenka: "Okay") was coded as positive. This illustrates a norm of arranged marriage.

This question was inspired by my conversations with some Mestizo men who were of the opinion (derived from second-hand stories they reported having heard) that Matsigenka fathers "give away" their daughters to non-Matsigenka men. The daughters apparently have no choice in the matter. This was explained to me as an exotic custom of the Matsigenka, meant to contrast with the more conservative norms held by the Mestizos who told me the stories. Such behavior was inconsistent with my observations in Tayakome, where I observed the formation of one young couple. In that case, the decision to be together seemed mutual, as I saw them openly flirting at a community-wide party before they moved in together. Neither set of parents seemed particularly happy about the match, evidenced by a public argument over the marriage between the two mothers during a community meeting. However, no one objected, as the decision was regarded to be that of the two young people. The marriage only lasted a few months before the young man left to work downriver and the young woman returned to her parents' house. However, during this incident I did not observe any norms that indicated a system of arranged marriage. However, a young married woman in Tayakome recounted to my wife that she was initially resistant to the idea of marrying her current husband. She said that her parents pressured her until eventually she agreed to marry him. Ultimately the choice was hers. I hypothesized that neither Matsigenka nor Mestizos would respond that the daughter should obey her parents and marry a man she does not want.

The vast majority of both Matsigenka (89%) and Mestizos (96%) responded "She must not obey her parents. She should marry who she wants" (Figure 2A). An example of a common Matsigenka explanation for this response, told from a father's perspective is, "Someone comes and says, 'I want to marry your daughter', [but] she doesn't want him, [she says] 'I don't want him'. There is someone else whom she loves . . . She doesn't want to marry him, [she says] 'I don't want him'. She gets mad at him, and she says 'Go away!'. She wants someone else." Although this explanation diverges slightly from the original question, it illustrates the autonomy of partner choice generally recognized for Matsigenka women of marriage age. An example of a typical Mestizo response is, "Before it was like that [i.e., arranged marriage]. The dad would say, 'You know what, daughter? I've found your ideal partner. He is for you.' If the girl is not in love with him, she doesn't pay attention to him. But the parents insist insist insist. The girl who is not in

love ends up having children, and that's when the fights come, the problem, the resentment against the father. [The daughter says to her father] 'I didn't want to, but you told me to, and now I did it!' So, I don't agree [with arranged marriage]. The person who choses the partner should be the one who is going to live and share their whole life [with the partner]."

Question 14, laborer is drunk (Wage Labor)

A man is hired to work two days: Monday and Tuesday. Monday night there is a party (Matsigenka: hosted by a Matsigenka). Should he go and get drunk? (Matsigenka: He goes and gets so drunk that he cant work on Tuesday. Is this ok?)

Un hombre está contratado trabajar dos días: lunes y martes. Lunes en la noche hay una fiesta. ¿El hombre debería ir y emborracharse?

Itinkame ipuinatakeri koriki yogari sidadi intsamaitera pitieti kuitagita: Lunes ontiri Martes. Lunes tsitenigeti aityo owidoki irashi ishaninka. Iriro iati, para ishinkitaka. Tenka iragavea intsamaitira Martes. Cameti o tera cameti?

A response of "Okay to go and become drunk" was coded as positive. This question is coded in the same way for the in-group and out-group latent axes. This illustrates a norm for the importance given to a labor contract. In my experience, all wage-labor contracts are verbal.

This question is inspired by my observation that there are a few members of both Matsigenka and Mestizo communities (all men, in my experience) who are occasionally so drunk that they neglect previously-made commitments. This behavior is generally frowned upon in both ethnic groups. However, because of the important role of manioc beer parties in Matsigenka society (see Question 4, above), and Matsigenka autonomy over their own production while living in Tayakome (i.e., a lack of work contracts), I hypothesized that Matsigenka might be more accepting of this behavior than Mestizos and would thus be more likely to respond "Okay".

The vast majority of both Matsigenka (78%) and Mestizos (100%) responded "Not okay", though there were a few Matsigenka who believed this behavior to be acceptable (Figure 2A). An example of a typical Matsigenka response is, "He goes [to the party]. He won't get drunk. He'll come back."

An example of a typical Mestizo response is,"He should not go [to the party] because he is contracted to work. Also, he should cultivate the values of responsibility, because if I don't keep my word with the woman who contracted me, at another time [in the future] she is not going to give me work. Surely I won't get any [work]. Then she'll point me out with an accusing finger, 'That is an irresponsible [person].' . . . to work one must go sober."

Appendix A.3. Statistical analysis

Appendix A.3.1. Overview

A series of Item-Response Theory (IRT) models (Bafumi et al., 2005; Fox, 2010; Jackman, 2001; Schacht and Grote, 2015; Bunce and McElreath, 2017; van der Linden, 2016), in a Bayesian framework (McElreath, 2016), was fit to interviewees' Personal Norm (ego) responses, in-group guesses, and out-group guesses. Responses to the 14 vignette questions for each of these targets (ego, in-group, and out-group) co-varied in a single dimension, represented by a latent axis for each target. Models estimated individual-level variance in interviewees' positions on the three latent target axes (random intercepts), covariance in interviewees' positions across target axes, covariance in question positions and discriminations within and across target axes, as well as the effects of binary individual-level predictors for inter-ethnic experience in the contexts of education, wage labor worker, wage labor employer, commerce, family, and community (see Section Appendix A.3.3). Statistical inference is drawn from distributions of posterior predictions for the probability of positive responses to the vignette questions (see Table 1) for each target. Kullback-Leibler divergences (McElreath, 2016; Kullback and Leibler, 1951) are calculated between probabilities of ego responses and in-group and out-group guesses for average Matsigenka and Mestizo individuals with different types of inter-ethnic experience (Figure 1). For all analyses, Mestizo residents of Boca Manu and Atalaya are grouped together in the category Mestizos, as the distributions of responses in these two communities are similar (Figure S3). Further details of analysis, including IRT model definitions and priors are provided below. Data and code in R (R Core Team, 2017) and RStan (Stan Development Team, 2018) to repeat this analysis can be found on Github at https://github.com/jabunce/bunce-2018-cross-cultural-competence.

Appendix A.3.2. IRT model structure

The norms measured by the vignette questions may covary, such that knowing how an individual answered one question gives you information about how she answered another question, and, in the ideal case, about how she answered all of the other questions. If true, then people's responses to the fourteen vignettes can be represented by a smaller number of latent dimensions, and, ideally, by a single latent dimension. I use Item Response Theory (IRT) models (Bafumi et al., 2005; Fox, 2010; Jackman, 2001; Schacht and Grote, 2015; Bunce and McElreath, 2017; van der Linden, 2016) in a Bayesian framework (McElreath, 2016) to show that, for this study, the vignette responses of each interviewee are well represented by a single dimension. This latent dimensional axis constitutes a convenient way to compare individuals on the basis of all fourteen measured norms simultaneously. It does not necessarily represent a unitary, overarching belief held by actual people (e.g., a meta-norm). For instance, it may be that the fourteen measured norms are functionally independent but happen to covary within this sample of people. Questions are coded such that a response corresponding to the positive pole of the latent axis is represented as 1, and a response corresponding to the negative pole is represented as 0. Such coding is arbitrary and can be reversed without changing interpretation of the model. From the perspective of this investigation, the vignette questions have no correct or incorrect answers, and thus responses coded as "positive" imply no judgment about the "correctness" of such a response. Although the latent axes of IRT models are often given interpretations (e.g., (Jackman, 2001; Schacht and Grote, 2015; Bunce and McElreath, 2017)) the conclusions below do not depend on interpretation of such constructed axes. The location on the latent axis of individual j is represented by the parameter α_j. I model α_j as a linear function of a random (i.e., individual-specific) intercept b_j, for each individual j, and various hypothesized predictors, including inter-ethnic commerce, wage labor, education, family, employer, and community experience:

$$\alpha_j = b_j + mCOM \cdot COM_j + \ ..., \qquad \text{for } j \text{ in } 1, ..., J \tag{A.1}$$

where J is the number of interviewees. An example fixed effect predictor, $mCOM \cdot COM_j$, is the product of the coefficient for inter-ethnic commerce experience and the binary commerce experience indicator for individual j.

To construct an IRT model, the linear model α_j is embedded within a logistic function. This allows us to simultaneously evaluate properties of each

individual and each vignette question with respect to the latent dimension. The probability that the response y of a particular interviewee j to a particular vignette question k is the positive response (column four of Table 1), $\Pr(y_{jk} = 1)$, is given by a logistic function (inverse logit) ranging between zero and one:

$$\Pr(y_{jk} = 1) = \text{logit}^{-1}[\gamma_k(\alpha_j - \beta_k)] \tag{A.2}$$

The domain (x axis) of this logistic function is the latent dimension. The slope at the function's inflection point, γ_k, is the degree to which a positive (1) versus negative (0) response to question k discriminates among individuals' positions on the latent axis. The location of the inflection point on the latent axis, β_k, is how close a person must be to the positive pole of the latent dimension in order for the model to predict that she give the positive response to question k. See Bunce and McElreath (2017) and Bafumi et al. (2005) for further explanation and illustrations.

In the present analysis, I fit an IRT model to personally-held (ego) norms, in-group guesses, and out-group guesses simultaneously. I had an a priori hypothesis that peoples' answers with respect to each of these targets (ego, in-group, and out-group, respectively) would covary, either positively or negatively. For instance, an individual's personal norms may coincide with the norms she believes are held by the majority of her co-ethnics, and diverge from the norms she believes are held by most members of the out-group. Thus, the model constructs a separate axis for ego responses, in-group guesses, and out-guesses, and I estimate the covariance among people's positions on these three latent axes (α_{jt} for person j on target axis t). I also allow covariance between individual question locations and discriminations on each axis (β_{kt} and γ_{kt} for question k on target axis t), as well as among the axes. Note that the three latent axes, because they are constructed separately by the IRT model, do not represent identical covariance structures among the questions (though, in these models, they are fairly similar). Thus, the positive pole of the ego axis does not correspond exactly with the positive poles of the in-group and out-group axes, as the coding of some questions changes on each axis in order to better fit the data, and the contributions of each question to the construction of the three axes (γ_k) are estimated separately for each axis t (though they are allowed to covary).

I incorporate ethnicity into the model by estimating the mean location of Matsigenka individuals separately from the mean location of Mestizo in-

dividuals on each axis, as well as ethnicity-specific covariances across axes. This has the same effect as including a main effect predictor for ethnicity in the linear model for α. I also allow the effects of inter-ethnic experience predictors (e.g., inter-ethnic commerce or education experience) for each axis to vary by ethnicity. This has the same effect as including an interaction of the predictors for ethnicity and inter-ethnic experience in the linear model for α. Thus, an IRT model incorporating ethnicity and inter-ethnic commerce experience is:

$$y_{jkt} \sim \text{Binomial}(1, p_{jkt}) \tag{A.3}$$

$$p_{jkt} = \text{logit}^{-1}[\gamma_{kt}(\alpha_{jt} - \beta_{kt})] \tag{A.4}$$

$$\alpha_{jt} = b_{jt} + mCOM_{t,\text{ETH}[j]} \cdot COM_j + \ \ldots \tag{A.5}$$

$$\begin{bmatrix} b_{t=\text{ego}} \\ b_{t=\text{in}} \\ b_{t=\text{out}} \end{bmatrix}_j \sim \text{MVNormal}\left(\begin{bmatrix} \mu_{b_{t=\text{ego}}} \\ \mu_{b_{t=\text{in}}} \\ \mu_{b_{t=\text{out}}} \end{bmatrix}_{\text{ETH}[j]}, \mathbf{S}_{\text{ETH}[j]} \right) \tag{A.6}$$

$$\mathbf{S}_{\text{ETH}[j]} = \begin{bmatrix} \sigma_{b_{t=\text{e}}} & 0 & 0 \\ 0 & \sigma_{b_{t=\text{i}}} & 0 \\ 0 & 0 & \sigma_{b_{t=\text{o}}} \end{bmatrix}_{\text{ETH}[j]} \mathbf{R}_{\text{ETH}[j]} \begin{bmatrix} \sigma_{b_{t=\text{e}}} & 0 & 0 \\ 0 & \sigma_{b_{t=\text{i}}} & 0 \\ 0 & 0 & \sigma_{b_{t=\text{o}}} \end{bmatrix}_{\text{ETH}[j]} \tag{A.7}$$

$$\begin{bmatrix} \beta_{t=\text{ego}} \\ \beta_{t=\text{in}} \\ \beta_{t=\text{out}} \\ \gamma_{t=\text{ego}} \\ \gamma_{t=\text{in}} \\ \gamma_{t=\text{out}} \end{bmatrix}_k \sim \text{MVNormal}\left(\begin{bmatrix} \mu_{\beta_{t=\text{ego}}} \\ \mu_{\beta_{t=\text{in}}} \\ \mu_{\beta_{t=\text{out}}} \\ \mu_{\gamma_{t=\text{ego}}} \\ \mu_{\gamma_{t=\text{in}}} \\ \mu_{\gamma_{t=\text{out}}} \end{bmatrix}, \mathbf{T} \right) \tag{A.8}$$

$$\mathbf{T} = \begin{bmatrix} \sigma_{\beta_{t=\mathrm{e}}} & 0 & 0 & 0 & 0 & 0 \\ 0 & \sigma_{\beta_{t=\mathrm{i}}} & 0 & 0 & 0 & 0 \\ 0 & 0 & \sigma_{\beta_{t=\mathrm{o}}} & 0 & 0 & 0 \\ 0 & 0 & 0 & \sigma_{\gamma_{t=\mathrm{e}}} & 0 & 0 \\ 0 & 0 & 0 & 0 & \sigma_{\gamma_{t=\mathrm{i}}} & 0 \\ 0 & 0 & 0 & 0 & 0 & \sigma_{\gamma_{t=\mathrm{o}}} \end{bmatrix} \mathbf{U} \begin{bmatrix} \sigma_{\beta_{t=\mathrm{e}}} & 0 & 0 & 0 & 0 & 0 \\ 0 & \sigma_{\beta_{t=\mathrm{i}}} & 0 & 0 & 0 & 0 \\ 0 & 0 & \sigma_{\beta_{t=\mathrm{o}}} & 0 & 0 & 0 \\ 0 & 0 & 0 & \sigma_{\gamma_{t=\mathrm{e}}} & 0 & 0 \\ 0 & 0 & 0 & 0 & \sigma_{\gamma_{t=\mathrm{i}}} & 0 \\ 0 & 0 & 0 & 0 & 0 & \sigma_{\gamma_{t=\mathrm{o}}} \end{bmatrix} \quad \text{(A.9)}$$

$$\begin{aligned} (mCOM_{t=\mathrm{e}}, mCOM_{t=\mathrm{i}}, mCOM_{t=\mathrm{o}})_{\mathrm{ETH=Mat}} &\sim \mathrm{Normal}(0,1) \\ (mCOM_{t=\mathrm{e}}, mCOM_{t=\mathrm{i}}, mCOM_{t=\mathrm{o}})_{\mathrm{ETH=Mes}} &\sim \mathrm{Normal}(0,1) \end{aligned} \quad \text{(A.10)}$$

$$\begin{aligned} (\mu_{b_{t=\mathrm{e}}}, \mu_{b_{t=\mathrm{i}}}, \mu_{b_{t=\mathrm{o}}})_{\mathrm{ETH=Mat}} &\sim \mathrm{Normal}(0,1) \\ (\mu_{b_{t=\mathrm{e}}}, \mu_{b_{t=\mathrm{i}}}, \mu_{b_{t=\mathrm{o}}})_{\mathrm{ETH=Mes}} &\sim \mathrm{Normal}(0,1) \end{aligned} \quad \text{(A.11)}$$

$$\begin{aligned} (\sigma_{b_{t=\mathrm{e}}}, \sigma_{b_{t=\mathrm{i}}}, \sigma_{b_{t=\mathrm{o}}})_{\mathrm{ETH=Mat}} &\sim \mathrm{Exponential}(2) \\ (\sigma_{b_{t=\mathrm{e}}}, \sigma_{b_{t=\mathrm{i}}}, \sigma_{b_{t=\mathrm{o}}})_{\mathrm{ETH=Mes}} &\sim \mathrm{Exponential}(2) \end{aligned} \quad \text{(A.12)}$$

$$(\mathbf{R}_{\mathrm{ETH=Mat}}, \mathbf{R}_{\mathrm{ETH=Mes}}) \sim \mathrm{LKJcorr}(4) \quad \text{(A.13)}$$

$$(\mu_{\beta_{t=\mathrm{e}}}, \mu_{\beta_{t=\mathrm{i}}}, \mu_{\beta_{t=\mathrm{o}}}) \sim \mathrm{Normal}(0,1) \quad \text{(A.14)}$$

$$(\mu_{\gamma_{t=\mathrm{e}}}, \mu_{\gamma_{t=\mathrm{i}}}, \mu_{\gamma_{t=\mathrm{o}}}) \sim \mathrm{HalfNormal}(0,1) \quad \text{(A.15)}$$

$$(\sigma_{\beta_{t=\mathrm{e}}}, \sigma_{\beta_{t=\mathrm{i}}}, \sigma_{\beta_{t=\mathrm{o}}}, \sigma_{\gamma_{t=\mathrm{e}}}, \sigma_{\gamma_{t=\mathrm{i}}}, \sigma_{\gamma_{t=\mathrm{o}}}) \sim \mathrm{Exponential}(2) \quad \text{(A.16)}$$

$$\mathbf{U} \sim \mathrm{LKJcorr}(40) \quad \text{(A.17)}$$

for j in $1, ..., J$ individuals, k in $1, ..., K$ questions, and t in (ego, in-group, out-group) target axes. This notation roughly follows that of McElreath (2016, pg 393) for varying-slopes models. See Bafumi et al. (2005) for notational changes if not all interviewees answered all questions. The subscript ETH[j] is an indicator for the ethnicity (Matsigenka or Mestizo) of individual j. Thus, $mCOM_{t,\mathrm{ETH}[j]}$ is the predictor for inter-ethnic commerce experience on target axis t for the ethnic group to which individual j belongs. It is multiplied by a binary indicator of individual j's commerce experience (0 or 1).

Informative Normal(0, 1) priors for mean intercepts (μ_b's), mean question locations (μ_β's), and predictor coefficients (e.g., $mCOM$'s), together

with exponential hyperpriors on standard deviations to control ceiling and floor effects common in such logistic models (McElreath, 2016, pg 363-364), effectively identify the model by constraining the position and scale of the α_j's and β_k's to fall within a reasonable distance (e.g., usually within two standard deviations) on either side of zero, resolving additive and multiplicative aliasing (Bafumi et al., 2005). Any choice of position and scale in IRT models is arbitrary and results in equivalent inference. Reflection (or rotational) invariance (Jackman, 2001; Bafumi et al., 2005) is not a concern as mean discriminations of all questions are positive (justifying positive priors on the μ_γ's), effectively polarizing the latent axis. **R** and **U** are correlation matrices, and are given conservative regularizing priors biasing against extreme correlations (McElreath, 2016, pg 394). These priors can be overcome by relatively strong signal in the data.

After fitting the model, the variance-covariance matrix **S** contains estimates of residual covariance among individual-specific (i.e., random) intercepts for individuals' locations (b_{jt}) across target axes, after accounting for the variance in location among individuals on each target axis explained by their inter-ethnic experience. For instance, how an individual answered the vignette questions (ego responses) may covary with how she guessed members of the out-group answered the questions (out-group guesses), even after accounting for the fact that certain types of inter-ethnic experience may affect both her own answers and her guesses about out-group individuals' answers. If true, estimates of the ego - out-group covariance contained in **S** will be non-zero.

To check the robustness of results to the effects of different predictors, I fit a series of 20 models varying in the fixed effect predictors included in the linear function for α_{jt} (Tables S1 and S2). Parameter estimation for each model was accomplished with RStan 2.17.3 Stan Development Team (2018), running four Hamiltonian Monte Carlo chains in parallel until convergence was suggested by a high effective number of samples (> 500) and $\hat{R}$ estimates of 1.00 (McElreath, 2016, pg 257). This entailed 3000 samples per chain, half of which were warm-up. In practice, a non-centered parameterization of the above model with Cholesky factorization of the correlation matrices **R** and **U** was fit in RStan (Stan Development Team, 2017, pg 151). I compared model fit with WAIC (McElreath, 2016, pg 191). Data and statistical analysis scripts in R (R Core Team, 2017) implementing RStan are available from Github at https://github.com/jabunce/bunce-2018-cross-cultural-competence.

Appendix A.3.3. Model predictors

Combinations of the following individual-level predictors are incorporated into linear models for individuals' locations on the latent axes (α_{jt}) in the IRT models.

Ethnicity

Interviewees were coded as Matsigenka if they self-identified as Matsigenka. Two siblings had a Matsigenka mother and a Mestizo father. Both of them had lived for several years in Matsigenka communities in Manu, self-identified as only Matsigenka upon questioning, and were coded as such. All non-Matsigenka in this study were classified as Mestizos. Within the study communities, the ethnic boundary between these two groups is stark, and I know of only three cases of marriage between Matsigenka and non-Matsigenka in the three study communities in the last three generations. Other interviewees who could plausibly have self-identified as bi-cultural, i.e., Matsigenka residing in Mestizo towns, all, without hesitation, self-identified as only Matsigenka. This coincided with how I observed them to interact with other Matsigenka and Mestizos. Further description of the relationship between Matsigenka and Mestizos in this population is provided in Bunce and McElreath (2017). Although this study does not explore in detail conceptions of ethnic identity, it is important to note that the relationship between ethnic identity and personally-held norms in cross-culturally competent individuals can be complex (Benet-Martínez and Haritatos, 2005; Schwartz et al., 2010). Sample sizes of Matsigenka and Mestizo interviewees are provided in Table 1.

Sex (1=Male)

Sex was coded according to self-identified gender. Sample sizes (# interviewees) for Matsigenka ego (female, male), in-group (female, male), out-group (female, male): (41, 38), (30, 27), (30, 27). Mestizos: (40, 42), (25, 21), (25, 21).

Age Category (Adolescent, Adult, Elder)

Many Matsigenka, especially older adults, do not know their exact age in years. For this reason I use age categories as predictors, estimating ages where necessary. Adolescents were younger than 20, adults were 20 to 50, and elders were older than 50. Note that binary predictors for only two of these categories need be included in the model at the same time. Adolescent

interviewees (ego, in-group, out-group): 9, 5, 5. Adults: 119, 76, 76. Elders: 33, 22, 22.

Education Experience (Edu $= 1$: attended school with Mestizos)
All Mestizos attended primary and/or secondary school with other Mestizos, so all were coded as 1. Several Matsigenka interviewees grew up outside of Tayakome and went to either a boarding- or non-boarding primary school with Mestizos. These individuals were coded as 1. Most Matsigenka in Tayakome attended primary school in Tayakome, with Matsigenka teachers and all Matsigenka students. If this was an interviewee's only education experience, she or he was coded as 0. There is no secondary school in Tayakome. A few Matsigenka from Tayakome attended boarding secondary schools with Mestizos outside of Tayakome for at least four of the requisite five years, and some had additional educational training after high school (e.g., for tour-guide certification). These boarding school attendees were coded as 1. Two Matsigenka interviewees attended a boarding secondary school for a few months before either being expelled or leaving because they did not like it. These interviewees were coded as 0. The average amount of inter-ethnic education experience among Matsigenka scored as 1 was approximately 6.5 years. Matsigenka interviewees coded as 1 (ego, in-group, out-group): 17, 10, 10.

Wage Labor Worker Experience (Lab $= 1$: wage labor experience with Mestizos)
All Mestizos were coded as having wage labor experience with other Mestizos. The vast majority of these interviewees, both men and women, had, at some point in their lives, worked for someone else to earn money, helped their spouse or relative earn money, and/or hired people to work for them. In contrast to the wage labor experience of most Matsigenka, many Mestizos are currently self-employed (e.g., banana farmers, small restaurant owners, boat builders) and do not have a "boss". Matsigenka were coded as 1, i.e., having wage labor experience with Mestizos, if they spent an approximate total of at least 1 month living with and working under Mestizos in their lifetime. This experience occurred in Mestizo towns, Mestizo-run tourist lodges or work camps (e.g., for logging or oil companies), or Mestizo-staffed park guard posts. Several Matsigenka interviewees attended boarding secondary school in Mestizo towns for several years and were hired as wage laborers on most weekends. These individuals were also scored as 1. The average amount

of wage labor experience among Matsigenka scored as 1 was approximately 3.5 years. Matsigenka interviewees coded as 1 (ego, in-group, out-group): 37, 26, 26.

Commerce Experience (Com = 1 : commerce experience with Mestizos)
All Mestizos were coded as 1, i.e., having commerce experience with other Mestizos, as all had purchased items in stores. Matsigenka were coded as 1 if they had ever directly bought or sold/traded items to Mestizos. Most Matsigenka had bought from Mestizo stores at least a few times in their life, although quantification based on memory was difficult. Matsigenka interviewees coded as 1 (ego, in-group, out-group): 65, 47, 47.

Family Experience (Fam = 1 : indigenous family experience)
All Matsigenka were coded as 1, i.e., having family experience with other Matsigenka. Mestizos were coded as 1 if, at some point in their lives, they had indigenous spouses or romantic partners, lived in the same household with indigenous people, and/or cared for indigenous children in the absence of the children's parents. Of the 36 Mestizos (43%) who had such experience, 26 (72%) had such experience with Matsigenka, as opposed to members of Yine or Harakmbut indigenous groups. 20 Mestizos with indigenous family experience (56%) raised Matsigenka children in their home for several months to years, with the consent of the children's parents. Sending children (ahijados or "entenados") to live with wealthier foster parents (sometimes official godparents, padrinos, but often not) is a custom in both the highlands and the lowlands of Peru. In the Manu region, the fostering is exclusively one-directional, with Matsigenka sending children to live with Mestizos, rather than vice-versa. Mestizo interviewees coded as 1 (ego, in-group, out-group): 36, 21, 21.

Wage Labor Employer Experience (Emp = 1 : experience employing Matsigenka)
Only one Matsigenka was coded as 1, as all other Matsigenka interviewees had never officially hired another Matsigenka as a wage laborer. 56 Mestizos (67%) were coded as 1 because they had, at some point, paid money to a Matsigenka in return for labor. Most of this labor was short-term, on the order of one, or a few, days (e.g., harvesting a plantain field). However, 15 (27%) of the Mestizos scored as 1 employed Matsigenka for at least several months at a time (e.g., as crew for tour boats during tourist seasons). Mes-

tizo interviewees coded as 1 (ego, in-group, out-group): 56, 34, 34.

Community Experience (Cty = 1 : lived in an indigenous community)
All Matsigenka were coded as 1, i.e., having lived in a majority-indigenous community at some point in their life. Matsigenka who were currently living in Boca Manu and Atalaya had previously spent years living in indigenous communities. Mestizos were coded as 1 if they had spent longer than one month living in one of the indigenous communities (Native Communities) in the region of Manu. These include some communities where the majority ethnic group is Yine or Harakmbut, rather Matsigenka, and also some Matsigenka communities outside of Manu National Park. The reason for including experience of Mestizos living in indigenous communities other than Matsigenka communities is my impression (as yet un-tested) that many Yine and Harakmbut have norms in many domains that are more similar to the norms of Matsigenka than to those of Mestizos. 31 Mestizos were coded as 1 (37%). Of these, 23 (74%) lived for at least one year in an indigenous community, and 12 (39%) had experience living in a majority-Matsigenka indigenous community. Mestizo interviewees coded as 1 (ego, in-group, out-group): 31, 19, 19.

Predictors for education, wage laborer, and commerce experience are meaningful only for Matsigenka interviewees (as such experiences are invariant among Mestizos), and are thus included in IRT models separate from those with predictors for family, employer, and community experience, which are meaningful only for Mestizo interviewees. This modeling strategy permits counterfactual contrasts of Matsigenka interviewees with education, wage labor, and commerce against Mestizos in general, and Mestizos with family, employment, and community experience against Matsigenka in general.

Appendix A.3.4. Model comparison and inference

Models with different combinations of predictors were compared using the Widely Applicable Information Criterion (WAIC) (McElreath, 2016, pg 191). Inference was based on the posterior distributions of predictions from models representing the hypotheses of interest, even if these were not the best fitting models (Tables S1 and S2).

Because the IRT models used in this analysis construct three separate latent axes (for ego responses, in-group guesses, and out-group guesses), interviewees' positions on these latent axes cannot be directly compared (contrast

with Bunce and McElreath (2017)). Instead, I translate posterior predictions of the models onto the probability scale (p_{jkt} in Eqs A.3 and A.4). This results in a posterior distribution of predictions for the probability that individual j gives the positive response for question k when the target of the question is t (i.e., ego, in-group, or out-group). Taking the average for an individual of a given ethnicity (i.e., ignoring estimated individual-level random effects) yields the distributions in Figure S8. For an average individual with given ethnicity and inter-ethnic experience, and a given target, I compute the mean probability across questions for each sample set of posterior predictions. This results in a posterior distribution of the mean probability that an average individual for a given target gives the positive response to an average (i.e., randomly-chosen) vignette question (e.g., column 1 of Fig 3).

Each posterior sample for a given individual, question, and target (p_{jkt}) is a probability distribution (i.e., the probability of giving the positive response and the probability of giving the non-positive response, $1-p_{jkt}$). To compare probability distributions, I use a measure of divergence. For comparing distributions for which one distribution represents an individual's guess about another distribution (e.g., a Matsigenka out-group guess about the average Mestizo ego response), I calculate Kullback-Leibler (K-L) divergence (Kullback and Leibler, 1951; McElreath, 2016), where the ego response is the K-L "target" (p) and the guess is the K-L "approximation" (q):

$$D_{KL}(p||q) = p_1 \ln\left(\frac{p_1}{q_1}\right) + p_0 \ln\left(\frac{p_0}{q_0}\right) \tag{A.18}$$

where p_1 and $p_0 = 1-p_1$ are the probabilities of positive and non-positive ego responses (respectively), and q_1 and $q_0 = 1 - q_1$ are the probabilities of positive and non-positive guesses (respectively). Notation roughly follows McElreath (2016, pg 179). K-L divergence is calculated for each pair of samples from the two posterior distributions to be compared, resulting in a posterior distribution of K-L divergence for each comparison of interest, e.g., guesses about the out-group responses versus ego responses of the out-group (see columns 4-7 of Figure S9 and Figures S14, S16, and S18). Such distributions represent the inaccuracy of guesses by average counter-factual individuals with given ethnicity and inter-ethnic experience (in units of the natural log of probability). Because K-L divergence does not permit examination of the direction of guess inaccuracy (e.g., over-estimation versus under-estimation of actual out-group mean ego responses), I also calculate simple contrasts of

posterior probabilities of guesses and ego responses (e.g., Figures S15, S17, and S19). Taking the mean K-L divergence across each sample set of questions yields the mean inaccuracy of guesses for an average individual, for an average (i.e., randomly-chosen) vignette question (e.g., columns 2 and 3 of Fig 3). To compare these inaccuracies of guesses, averaged across questions, by average individuals with differing counter-factual inter-ethnic experience, I calculate contrasts of their respective distributions of K-L divergence (e.g., Fig 4, and rows 2 and 3 of Figure S13).

For comparing probability distributions for which one distribution does not represent an individual's guess about another distribution (e.g., a Matsigenka ego response and an Mestizo ego response), I calculate Jeffreys divergence (Kullback and Leibler, 1951; Jeffreys, 1948), which is a symmetric version of K-L divergence between two distributions p and q: $D_J(p;q) = D_{KL}(p||q) + D_{KL}(q||p)$ (Crooks, 2008) (see columns 1-3 of Figure S9). Taking the mean Jeffreys divergence across questions yields the divergence in the probabilities that the individuals represented by the two distributions gave the same response to an average (randomly-chosen) vignette question (e.g., Figure S11, and row 1 of Figure S13). Because Jeffreys divergence does not permit examination of the direction of the difference between distributions (i.e., which of the two probabilities of a positive response is greater), I also calculate simple contrasts of the posterior probabilities for each question (e.g., Figure S10), as well as for the mean probabilities across questions (e.g., Figure S12).

Inference about cross-cultural competence is drawn from comparisons of the inaccuracies (i.e., divergences among posterior probability distributions) of in-group and out-group guesses made by counter-factual respondents differing in ethnicity and inter-ethnic experience (Figure 1).

Appendix B. Supplementary Discussion

Appendix B.1. Additional Results and Discussion

Appendix B.1.1. Ethnicity differences in personal norms

Figure 2A shows that, for all fourteen vignette questions, a larger proportion of Matsigenka than Mestizos gave positive Personal Norm (ego) responses. These raw proportions suggest an overall ethnic difference in the distributions of the norms applied by the interviewees to answer the questions, and they demonstrate the utility of the interview instrument to distinguish between these two ethnic groups. This is further supported by the results of

the IRT models (Figures 3 and S2), which show a notable difference in the probability that Matsigenka and Mestizos give the positive ego response for an average (i.e., randomly chosen) vignette question.

Figure 2B and C show that Matsigenka and Mestizos guessed differently about how their respective co-ethnics (in-group) answered most questions, and about how their respective out-groups answered most questions (i.e., points generally fall far from the diagonal of equal proportions). This is supported by the results of the IRT models, shown in Figure S8. The fact that both Matsigenka and Mestizos guessed that in-group and out-group responses would differ for some questions (Figure S10) supports the conclusion that interviewees understood the guessing task in the interviews.

Posterior estimates of the discrimination parameters (γ_k) for most vignette questions on all target axes are reliably greater than zero (Figures S4-S7). This indicates that most questions can reasonably discriminate among individuals along a single latent axis for each target (Jackman, 2001).

Appendix B.1.2. Model comparison

Coefficient estimates for all models, including models m4, m11, and m19 used for inference above and in the main text, are provided in Tables S1 and S2. Exploratory models including sex and categorical age predictors achieved <1% of model weight, and the coefficients of each of these predictors could not be distinguished from zero (m12 and m20 in Tables S1 and S2). Furthermore, coefficient estimates for the inter-ethnic experience predictors were not distinguishable from those of the models used for inference, above. This gives me confidence that inclusion of sex and age in the models has little effect on the results.

Appendix B.1.3. Covariance of personal norms and out-group guesses

Matsigenka interviewees tended to guess that both the in-group and the out-group answered the vignette questions the way that the interviewees themselves answered them. This is seen in the positive covariance between Matsigenka individual locations on the latent ego axis and on the latent in-group and out-group axes (Figures S20-S22). Because there is considerable variance in Matsigenka ego locations, this covariance leads to considerable variance in location on the in-group and out-group axes. In contrast, Mestizos exhibited much less covariance in individual locations among the latent target axes, and much less variance on each target axis. These results suggest that there is much variance in the norms held by Matsigenka. However,

regardless of which norms she holds, each individual Matsigenka tends to believe that Mestizos hold norms like hers (with the notable exception of norms for healthcare: Figure S10). In contrast, it appears that Mestizos exhibit considerably less variation in how they perceive the norms of both Matsigenka and their fellow Mestizos, suggesting a shared stereotype of both Matsigenka and Mestizo norms. Interestingly, these seemingly different strategies for guessing about the norms of the in-group and out-group result in more inaccurate out-group guesses and less inaccurate in-group guesses for Matsigenka relative to Mestizos (Figure S2).

Appendix B.1.4. Cross-cultural competence and familiar out-group members

It could be the case that Matsigenka and Mestizos derive their perceptions of out-group norms from the norms of particularly familiar out-group individuals. Figure S23 shows that the Matsigenka individuals most familiar to Mestizos in this population have norms that are considerably different from the norms of the average Matsigenka. In contrast, the norms of the Mestizo individuals most familiar to Matsigenka have norms that are more similar to the norms of the average Mestizo. Thus, if individuals were basing out-group guesses on the norms of particularly familiar out-group members, we would expect Matsigenka out-group perceptions to be more accurate than those of Mestizos. The fact that this appears not to be the case (Figure S2) suggests either that such a strategy is not used, or that it is used to a lesser extent by Matsigenka than by Mestizos.

Appendix B.1.5. Domain-specificity of cross-cultural competence

The measure of overall cross-cultural competence developed here is driven by more accurate out-group guesses in a subset of norm domains plausibly linked to particular types of inter-ethnic interaction, e.g., education norms for Matsigenka education experience (Figure S14), labor norms for Mestizo employer experience (Figure S16). This suggests that knowledge of out-group norms is being put to use, and may thus covary with other characteristics often viewed as components of cross-cultural competence, such as motivation and skill (Spitzberg and Changnon, 2009).

Appendix B.1.6. Errors in Matsigenka and Mestizo guesses

As noted in the main text, and as can be seen in Figures 2, S8, and S9, in general, Matsigenka tended to guess that Mestizos answered question 7 (What do you do first when you have a respiratory illness?) by saying

that they would go to the health post (i.e., the response arbitrarily coded as positive). In fact, 96% of Matsigenka participants made this guess. Such a guess was highly inaccurate, as nearly 80% of Mestizos actually responded that they would first use home remedies for such an illness. However, in light of my experience living in these communities, the inaccuracy of Matsigenka out-group guesses for this question seems predictable in hindsight. Matsigenka generally perceive respiratory illnesses (merentsi) as originating among Mestizos, and subsequently being carried into Tayakome by Matsigenka returning from travel outside the community among Mestizos. I know of no Matsigenka-specific remedy for merentsi, although tobacco snuff is sometimes used as a prophylactic. The Mestizo-run health post in Tayakome dispenses medicine, usually in the form of pills and injections, to community members suffering from merentsi. Therefore, from a Matsigenka perspective, merentsi is an illness of Mestizo origin for which Mestizos have medicine that Matsigenka can obtain only at a Mestizo-run health post. With this in mind, it seems logical that Matsigenka would expect Mestizos who have merentsi to seek treatment at a health post. However, as shown, most Mestizos in fact rely heavily on home remedies for minor respiratory illnesses. Several Mestizo interviewees pointed out to me that medicine obtained from the health post is generally ineffective against respiratory illnesses, and that the best course of action is to rest, use a home remedy such as hot tea, and let the illness run its course. In my experience, Matsigenka visitors to Mestizo towns generally have few opportunities to observe Mestizos with respiratory illnesses (as such Mestizos are generally home resting), and therefore they may have little exposure to the types of treatments that Mestizos rely on in this context.

It is also apparent in Figures 2, S8, and S9, that the vast majority (96%) of Mestizos guessed that their co-ethnics answered question 5 (Is it okay for a teacher to hit a student who doesn't learn?) by saying that hitting a student is unacceptable. While such a guess is strictly accurate at the individual level, in that more than half of Mestizos gave this response, in the aggregate it is statistically inaccurate, because a substantial minority (37%) of Mestizos responded that teachers should hit students in order to motivate them to study harder. In my experience, most Mestizos are familiar with current Peruvian law that prohibits corporal punishment of students by teachers. However, many adults recalled how they themselves were subjected to painful treatment by their own primary and secondary school teachers when they lapsed in their schoolwork. A common saying among Mestizo interviewees is

that, in previous generations, “La letra entra con sangre” (The letter enters with blood), meaning that knowledge must be beaten into students. Several interviewees reminisced that the threat of such punishment motivated them to study harder when they were young, and that current students are much lazier because these punishments are no longer allowable. It seems plausible that Mestizos who hold such beliefs are unlikely to express them publicly, as these beliefs conflict with current law. This may lead to underestimation of the number of other members of their community who actually think like they do. Similarly, the norm for the corporal punishment of students is not acted upon by those Mestizos who personally hold it because it is against the law.

Appendix B.1.7. Power differences and cross-cultural competence

Fiske (1993) proposed a theory in which members of a less powerful subgroup form more accurate impressions of individuals in a powerful subgroup, while powerful individuals form potentially less-accurate stereotypical impressions of less-powerful individuals. The reasoning is that it behooves the powerless to pay close attention to powerful individuals, whose behavior can have a large impact on their lives. In contrast, powerful individuals need pay less attention to the powerless, whose behavior has little impact on their lives. In the present ethnographic context, despite large differences in the material wealth of manufactured items, there is little evidence that most Matsigenka feel themselves to be inferior to, or less powerful than, Mestizos in most important domains of life (Bunce and McElreath, 2017). Thus, there is no a priori expectation that the out-group guesses of an average Matsigenka would be more accurate than those of an average Mestizo, and indeed, such a prediction is not supported by these data (Figure S2). However, note that this methodology is amenable to testing Fiske's theory in ethnographic contexts where power differences between groups are salient.

Appendix B.1.8. Mestizo inter-ethnic experience and cross-cultural competence

Figure 3B shows that, for Mestizos, indigenous family experience is associated with lower cross-cultural competence than is experience employing Matsigenka wage laborers or experience living in an indigenous community. This may seem surprising, given that family members engage in important coordination interactions in many domains on a daily basis. Thus, one might expect members of a functioning multi-ethnic family to be highly

cross-culturally competent. However, note that, for the majority of Mestizos who had family experience with Matsigenka, this experience consisted of raising Matsigenka foster-children (see Section Appendix A.3.3), usually in the context of these children attending primary and secondary schools in Mestizo towns. In my observation, many of these Mestizo adults felt it to be their duty to instill morals and values in the children under their charge. These morals and values usually took the form of idealized Mestizo-typical norms (e.g., Mestizo interpretations of cleanliness, obedience, responsibility, respect for elders, diligence in schoolwork, etc.). Thus, because of the power differential between Mestizo foster-parents and Matsigenka children, Mestizos with inter-ethnic family experience had no need to learn Matsigenka-typical norms. They were, for the most part, engaged in imposing Mestizo-typical norms on the Matsigenka children with whom they interacted.

Figure 3B also suggests that, compared to indigenous family experience and no inter-ethnic experience, the experience of living in an indigenous community is associated with Mestizos who hold more Matsigenka-typical norms and who are more cross-culturally competent. I have not had the opportunity to observe Mestizos living as minorities in indigenous communities outside of Manu National Park. Mestizos (other than the health post technician) are not permitted to live in Tayakome or other Matsigenka communities inside the park. Thus, any explanation for why this type of experience is more strongly associated with Matsigenka-typical norms than is the experience of hiring Matsigenka wage laborers, should be regarded as tentative. I hypothesize that Mestizo residents in indigenous communities necessarily engage in coordination interactions in many domains (e.g., commerce, employment, community obligations) primarily with the majority indigenous residents, who likely hold norms different from those of most Mestizos. As minorities, Mestizo residents likely have lower bargaining power in such interactions, and consequently adopt and internalize norms typical of the indigenous residents, analogous to Matsigenka boarding-school students adopting norms typical of their Mestizo teachers and care-takers. Alternatively, it is also likely that only Mestizos who hold norms most similar to those of indigenous people are willing to settle and live in indigenous communities. Either mechanism, or both, could be responsible for the results found in this study.

Appendix B.2. Hypotheses for cultural dynamics

Appendix B.2.1. Prediction 1: Loss of cultural norm diversity

The prediction is made in the main text that the long-term sustainability of ethnically-structured cultural norm diversity in a society is unlikely if members of one ethnic group become cross-culturally competent as a consequence of adopting personally-held out-group norms. In such a situation, cross-cultural competence itself will eventually disappear. This prediction is inspired by the model of language shift in Kandler (2009). To think through this argument, I sketch a hypothetical model.

Assume a population in which there are two varieties of coordination norm, designated norm 1 and norm 2, and each individual personally holds only one of the two norms. During coordination interactions, both individuals receive a payoff $\beta > 0$ if they both employ the same norm, and both receive a payoff of 0 if they each employ different norms. Assume interaction partners are drawn at random from within the entire population. There are three states that people may occupy: 1) individuals in state P_1 personally hold norm 1 and always employ norm 1 when they attempt coordination interactions with all other individuals; 2) individuals in state P_2 personally hold norm 2 and always employ norm 2 when they attempt coordination interactions with all other individuals; and 3) individuals in state C (cross-cultural competence) personally hold either norm 1 or norm 2, but all are capable of employing either norm 1 or norm 2 in any attempted coordination interaction. The norm that a C individual personally holds is the norm that she employs when interacting with another C individual. When she interacts with a P_1 or P_2 individual, she always employs norm 1 or norm 2, respectively.

To begin, assume an initial population comprising P_1 and P_2 individuals. At each time step t, allow P_1 individuals to enter state C with probability $r_{P1\to C} > 0$, but only on the condition that they adopt norm 2 as their personally-held norm. Under the above conditions, C individuals receive higher coordination payoffs, on average across all interactions, than either P_1 or P_2 because they can successfully coordinate with everyone in the population. All other state transition probabilities, $r_{P1\to P2}$, $r_{P2\to P1}$, $r_{P2\to C}$, $r_{C\to P1}$, $r_{C\to P2}$, are initially assigned values of zero. Under these conditions, all P_1 individuals will transition to C, and the population will evolve to comprise only P_2 and C individuals, all of whom personally hold norm 2. Norm diversity has not been lost, in the sense that all C individuals still retain knowledge of,

and the ability to coordinate using, norm 1. However, because all C individuals employ norm 2, i.e., the norm that they personally hold, when interacting with other C individuals, norm 1 will, in practice, cease to be employed in the population. Thus, because any C individual who forgets norm 1 suffers no decrease in average payoff (in the context of this hypothetical model), any non-zero probability of such forgetting on the part of C individuals, i.e., $r_{C \to P2} > 0$, will lead to the loss of both cross-cultural competence and norm diversity, as the entire population transitions to state P_2.

Such dynamics would appear to parallel the language dynamics investigated formally by Kandler and colleagues (Kandler, 2009; Kandler et al., 2010), in which the only equilibria are those in which one language, and all bilingual speakers, go to extinction. Kandler's models show that requiring language adoption to proceed through a transition state of bilingualism can slow, and even reverse, the direction of this dynamic compared to models where individuals switch directly from being speakers of only one language to being speakers of only a different language. However, in the absence of external intervention in these models, bilingualism can never result in a mixed equilibrium where both languages are simultaneously present in the population.

Appendix B.2.2. Prediction 2: Sustainability of cultural norm diversity

A second prediction is made in the main text that the sustainability of norm diversity in a population is more likely if cross-cultural competence is acquired while retaining in-group norms. Using the above example, this corresponds to a situation in which P_1 individuals are allowed to enter state C, but only on the condition that they retain norm 1 as their personally-held norm. C individuals coordinate with P_2 individuals using norm 2, which is not C's preferred (personally-held) norm. In the terminology of Carvalho (2017), P_2 and C are misaligned (coordination is possible only if members of one group employ norms that they do not prefer), but they are not incompatible (such that coordination would be impossible, e.g., between P_1 and P_2). Carvalho (2017) showed formally that coordination between P_2 and C using norm 2 can evolve, provided that the payoffs of coordination to C individuals are substantially greater than the payoffs to C individuals of miscoordinating while attempting to employ norm 1. If this condition holds (along with the others above), then C individuals initially receive greater average coordination payoffs than either P_1 or P_2 for the same reason as in the previous model. Allowing $r_{P1 \to C} > 0$, while holding all other transition probabilities

at zero, will result in all P_1 individuals transitioning to the cross-cultural competence state C.

In contrast to the previous scenario, now all C individuals employ norm 1 (their personally-held norm) when interacting with other C individuals. Both norm 1 and norm 2 are actively employed in the population, and, as in the previous model, cultural norm diversity has not decreased. However, if there is some subjective cost $\delta > 0$ to coordinating using one's non-preferred norm, then, once P_1 individuals disappear (or become sufficiently rare), P_2 individuals receive a higher average payoff than C individuals. This is because P_2 individuals coordinate with everyone in the population using their preferred norm 2. In contrast C individuals coordinate with other C individuals using their preferred norm 1, but their coordination payoffs are reduced by an amount δ whenever they coordinate with P_2 individuals, as, in such cases, they always employ their non-preferred norm, norm 2. Thus, a mutant C who adopted norm 2 as her personally-held norm and forgot norm 1 (effectively transitioning to state P_2), would increase her average coordination payoff. If we then give C individuals the option of transitioning to P_2, we would find that $r_{C \to P2} > 0$, leading to the loss of both cross-cultural competence and norm diversity, as the entire population transitions to state P_2. This equilibrium is identical to that reached in the previous model.

However, what if there are inter-ethnic differences in bargaining power during coordination interactions? Bunce and McElreath (2018) showed that such power differences can by operationalized as inter-ethnic differences in coordination payoffs. The individual receiving the highest payoff in a coordination interaction has the least bargaining power. In the above example, define $\beta > 0$ as the coordination payoff received by each of two individuals with equal bargaining power who both hold the same preferred norm. Let P_2 individuals receive a lower benefit, $\beta - \theta$ (where $\theta > 0$), from coordination interactions with C individuals, meaning that P_2 individuals have greater bargaining power. Define the benefit to individual i received during a coordination interaction with individual j as $b_{i \leftarrow j}$. Under the above conditions: $b_{C \leftarrow C} = b_{P2 \leftarrow P2} = \beta$; $b_{C \leftarrow P2} = \beta - \delta$; $b_{P2 \leftarrow C} = \beta - \theta$. If the decrease in the inter-ethnic coordination payoff suffered by P_2 individuals, θ, is at least as large as the cost, δ, suffered by C individuals as a result of having to coordinate with P_2 individuals using C's non-preferred norm 2, then it is plausible to imagine conditions in which $r_{C \to P2} = 0$ and both cross-cultural competence and norm diversity are sustainable at equilibrium. Thus, in order for this path to cross-cultural competence to result in the long-term sustain-

ability of cultural norm diversity in the population, we must add a cost θ to inter-ethnic coordination suffered by only one of the two groups, which outweighs the cost δ to inter-ethnic coordination suffered by cross-culturally competent members of the other group. Equivalently, we could add an additional benefit to inter-ethnic interaction that accrues only to cross-culturally competent individuals suffering the coordination cost δ. If this additional benefit is as least as great as δ, a mixed equilibrium can be stable, resulting in sustainable norm diversity.

The following is one hypothetical illustration of such a situation. Following Bunce and McElreath (2018), imagine minority indigenous Matsigenka and majority Mestizo colonists interacting in the domain of education using either Matsigenka-typical or Mestizo-typical pedagogical norms (e.g., silent observation versus participatory questioning, respectively, on the part of students). Assume both ethnic groups are of equal size and student-teacher pairings are randomized. Possible coordination combinations include: Matsigenka teacher-Matsigenka student, Matsigenka teacher-Mestizo student, Mestizo teacher-Mestizo student, Mestizo teacher-Matsigenka student. Assume all Matsigenka students and teachers are cross-culturally competent, such that they can coordinate using either Matsigenka or Mestizo pedagogical norms, yet all personally hold (prefer) Matsigenka norms. Thus, when a Matsigenka coordinates with a Mestizo, the Matsigenka's coordination payoff is reduced by an amount $\delta > 0$, which represents the subjective cost of having to coordinate using a non-preferred norm. All Mestizo teachers and students prefer, and always coordinate using, Mestizo norms. Following Bunce and McElreath (2018), assume all students receive higher coordination payoffs than all teachers, and all teacher coordination payoffs are equal. As above, the coordination payoff to student i from instruction by teacher j is $b_{i \leftarrow j}$. Assume the following conditions: $b_{Mat \leftarrow Mat} = b_{Mes \leftarrow Mes} = \beta$; $b_{Mat \leftarrow Mes} = \beta - \delta$; $b_{Mes \leftarrow Mat} = \beta - \theta$. Such would be the case if, for instance, Matsigenka students value the knowledge imparted by both Matsigenka and Mestizo teachers equally (e.g., skills to survive in the forest and skills to earn money, respectively), yet Mestizo students only value the knowledge imparted by Mestizo teachers (e.g., if Mestizos rarely venture into the forest), and thus suffer an opportunity cost $\theta > 0$ when they are taught by Matsigenka teachers. If this opportunity cost to Mestizo students, θ, is greater than or equal to δ, the subjective cost suffered by Matsigenka students from having to coordinate with Mestizo teachers using the Mestizo pedagogical norm (these students' non-preferred norm), then cross-cultural competence and

Matsigenka pedagogical norms could be sustainable (in the context of this hypothetical example). The reason is because, given equal numbers of Matsigenka and Mestizos and randomized student-teacher pairings, Matsigenka students would receive higher average payoffs than would Mestizo students across all coordination interactions (intra-ethnic interactions: β; inter-ethnic interactions: $\beta - \delta > \beta - \theta$). Thus, even if it were possible for Matsigenka students to adopt the Mestizo pedagogical norm as their personal norm and forget the Matsigenka norm, effectively converting themselves into Mestizos, they would not do so, as such a transition would entail a decrease in average coordination payoffs.

This discussion has sketched hypothetical models to argue that the two predictions in the main text are plausible in theory. A formal analysis of more sophisticated models is required to investigate whether these predictions are supported after relaxing some of the more unrealistic constraints imposed above for the sake of simplicity.

Appendix C. Supplementary Figures and Tables

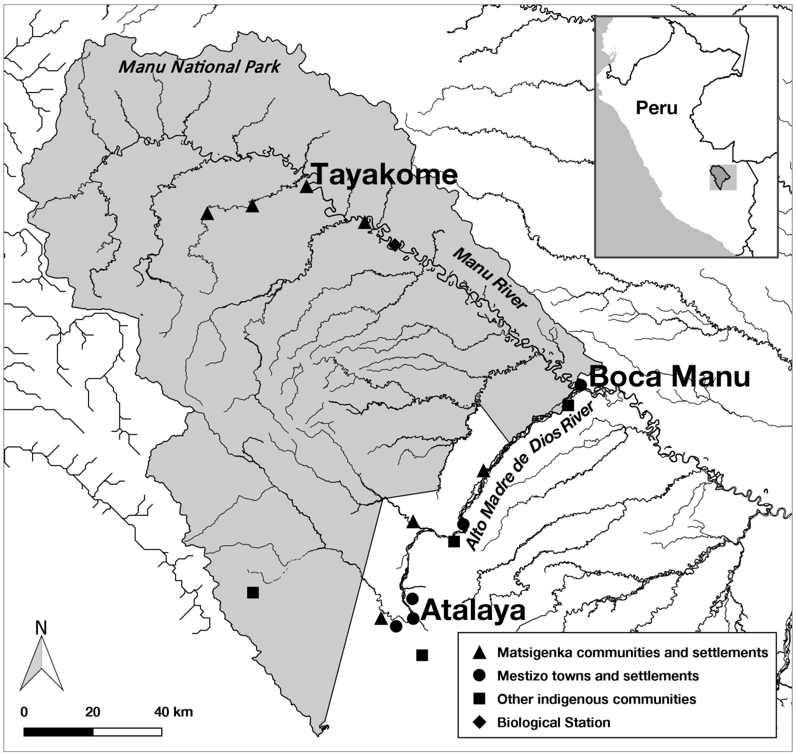

Figure S1: Map of study communities. Map of the Matsigenka study community of Tayakome, and the Mestizo study communities of Boca Manu and Atalaya, as well as the locations of other nearby settlements in and around Manu National Park (in gray), Peru. Assistance with map creation was provided by Ronny Barr.

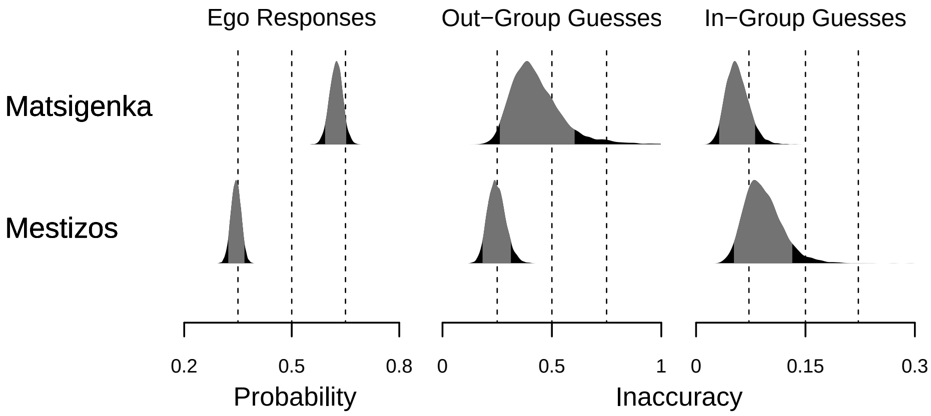

Figure S2: Personally-held norms and inaccuracy of in-group and out-group guesses. Left column: distributions of mean probabilities of a positive Personal Norm (ego) response across all 14 vignette questions, by ethnicity. Center column: distributions of mean (across questions) Kullback-Leibler (K-L) divergence between the probability that an average out-group member gave the positive ego response and the average probability of guessing that an average out-group member gave the positive ego response: $D_{KL}(\text{out-group ego}||\text{guess about out-group})$. This represents the mean inaccuracy of out-group guesses, by ethnicity. Right column: distributions of mean K-L divergence between the probability that an average in-group member gave the positive ego response and the average probability of guessing that an average in-group member gave the positive ego response. This represents the mean inaccuracy of in-group guesses, by ethnicity. Relative cross-cultural competence is defined as lower inaccuracy of out-group guesses and no greater inaccuracy of in-group guesses. Divergence and contrasts of ego responses, out-group inaccuracy, and in-group inaccuracy for Matsigenka and Mestizo inter-ethnic experience types are shown in Figures S12-S15. Distributions of means are derived from posterior predictions of the IRT model m4 shown in Tables S1 and S2. 90% highest posterior density intervals (HPDI) are shown in grey. Vertical lines are drawn for visual guidance. Note that these are posterior distributions of means, not distributions of interviewee responses. There is generally more uncertainty about the counter-factual means in panels A and B of Figure 3 than there is about the overall means shown here (compare variances). Similarly, the mean of the counter-factual mean distributions in panels A and B of Figure 3 need not coincide with the overall mean distribution for a given ethnicity in this figure.

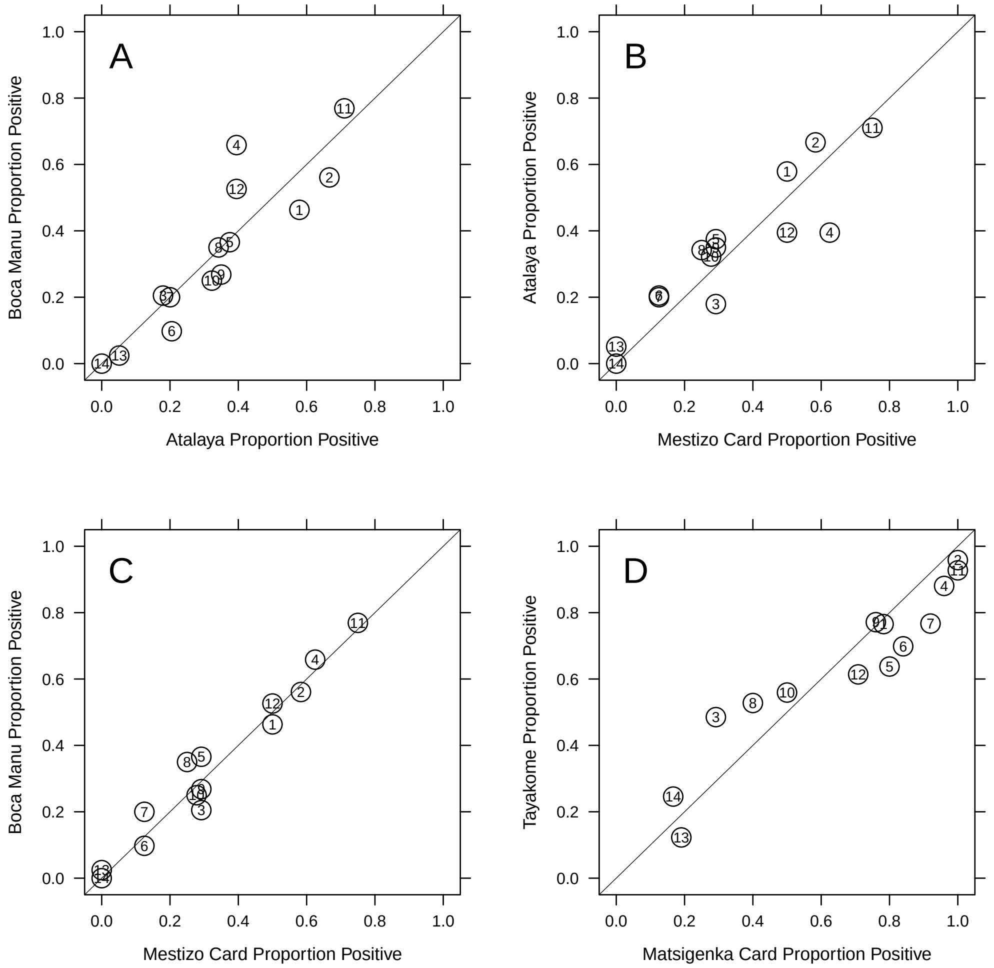

Figure S3: Raw proportions of personally-held norms. Proportions of Boca Manu Mestizos (n=41), Atalaya Mestizos (n=40), Tayakome Matsigenka (n=73), the sample of Mestizo-community respondents represented by cards (n=24), and the sample of Tayakome respondents represented by cards (n=25) giving the positive response to the fourteen vignette questions in Table 1. All proportions are for Personal Norms (ego). The diagonal is the line of equal proportions. The vertical (or horizontal) distance from a point to the diagonal is the difference in proportion between groups represented on the axes. A) Mestizos in Boca Manu and Atalaya. B) Atalaya Mestizos and the sample of Mestizo-community cards C) Boca Manu Mestizos and the sample of Mestizo-community cards. D) Tayakome Matsigenka and the sample of Tayakome cards. Note that, in all figures, proportions are relatively similar, i.e., close to the diagonal (compare to Figure 2).

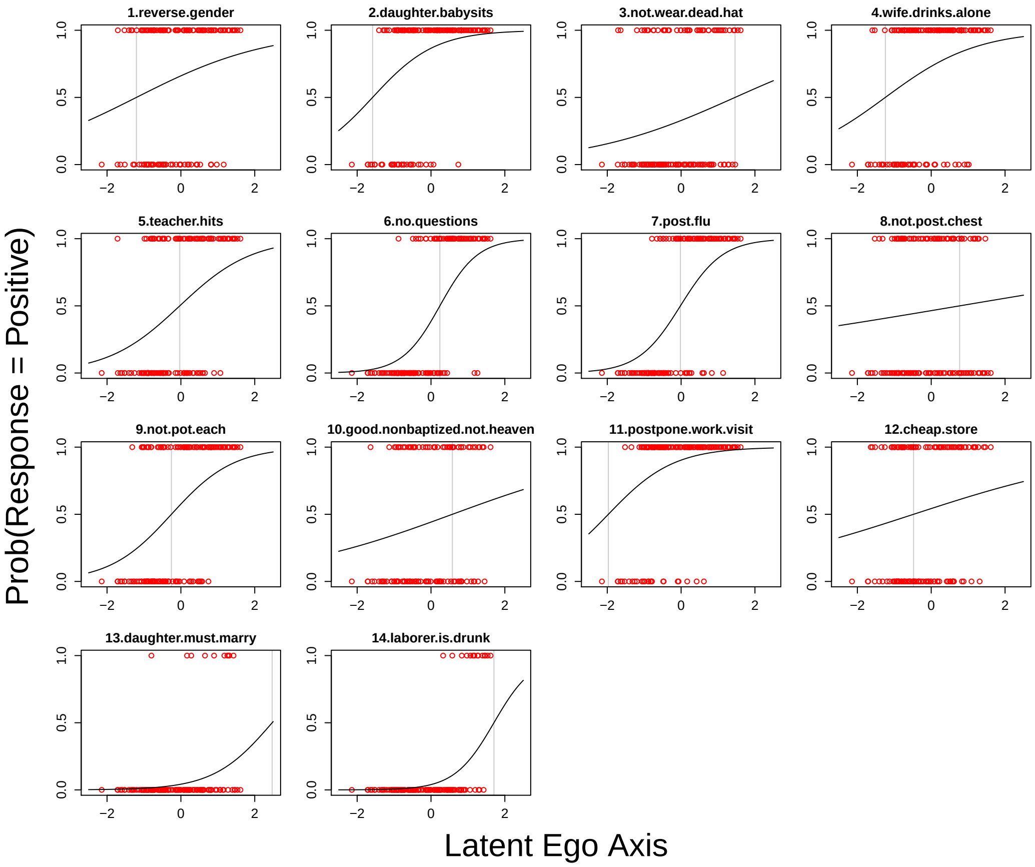

Figure S4: IRT question characteristics for personally-held norms. Logistic functions generated from the mean posterior location (β_k, x-value of vertical grey line) and discrimination (γ_k, slope of the function at intersection with vertical line) of each of the 14 vignette questions on the latent ego axis (target $t = 1$), derived from an IRT model with a random effect for individual and no fixed effect predictors (m1 in Tables S1 and S2 below). Questions with higher absolute value discrimination contribute more to the construction of the latent axis. The discriminations of questions 8 and 12 are only marginally distinguishable from zero (Figure S7). Discriminations that are distinguishable from zero suggest that those questions map well onto a single dimension (Jackman, 2001). The posterior mean location of each individual on the latent axis is plotted in red. A person's y-value of 1 or 0 corresponds with a positive or negative response, respectively, to the given question (defined in Table 1).

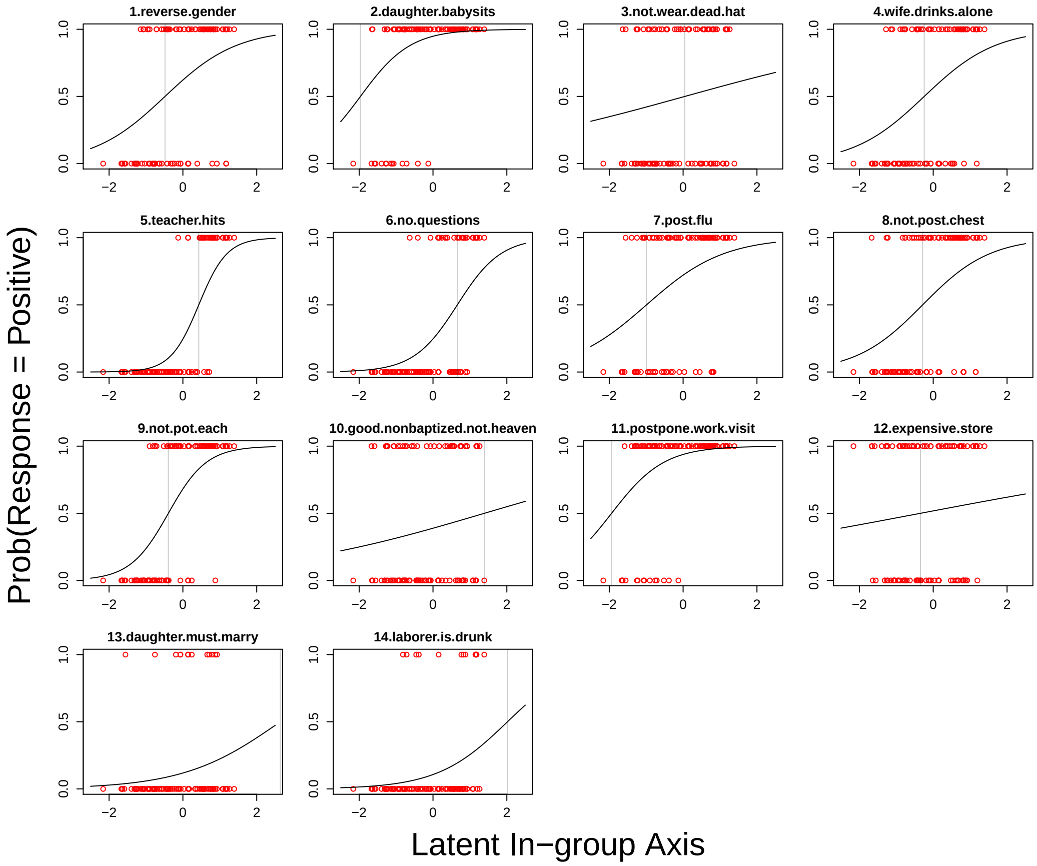

Figure S5: IRT question characteristics for in-group guesses. Analogous to Figure S4 for in-group guesses (target $t = 2$). Note that the coding of question 12 is reversed from that for the latent ego axis. The discriminations (slope at the inflection point) of questions 3 and 12 are only marginally distinguishable from 0 (Figure S7).

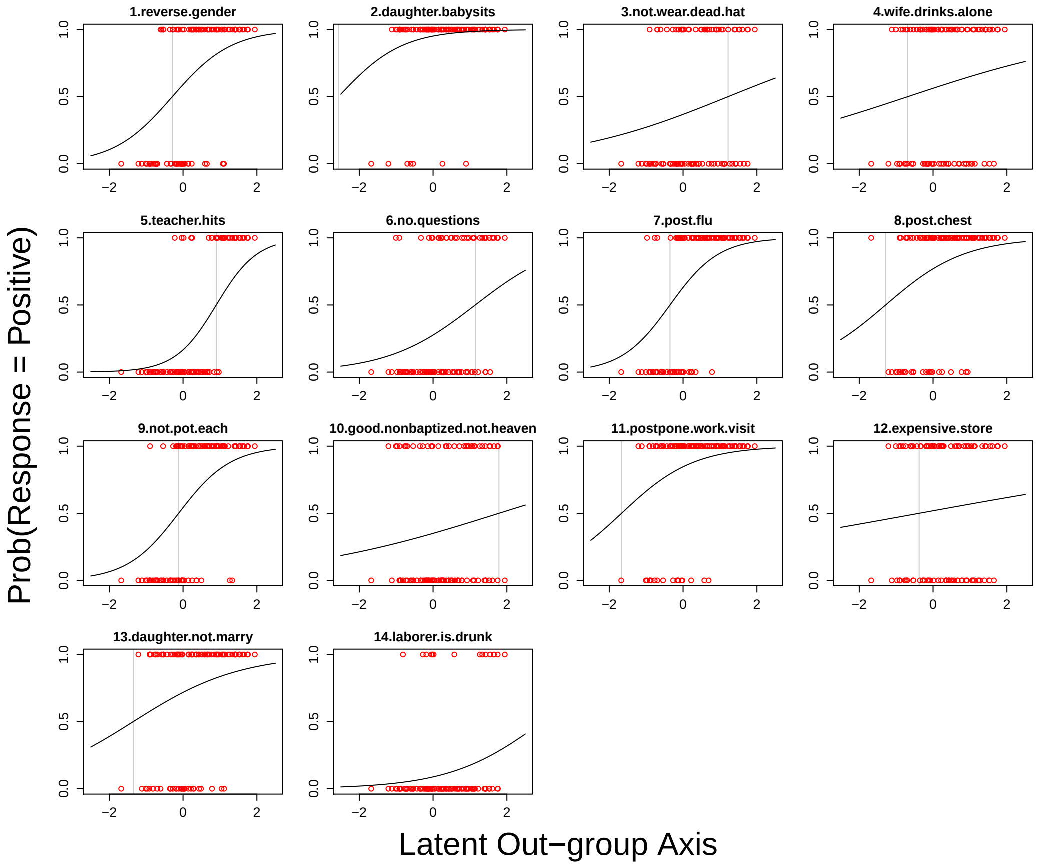

Figure S6: IRT question characteristics for out-group guesses. Analogous to Figure S4 for out-group guesses (target $t = 3$). Note that the coding of questions 8, 12, and 13 is reversed from that for the latent ego axis. The discriminations (slope at the inflection point) of questions 4 and 12 are only marginally distinguishable from 0 (Figure S7).

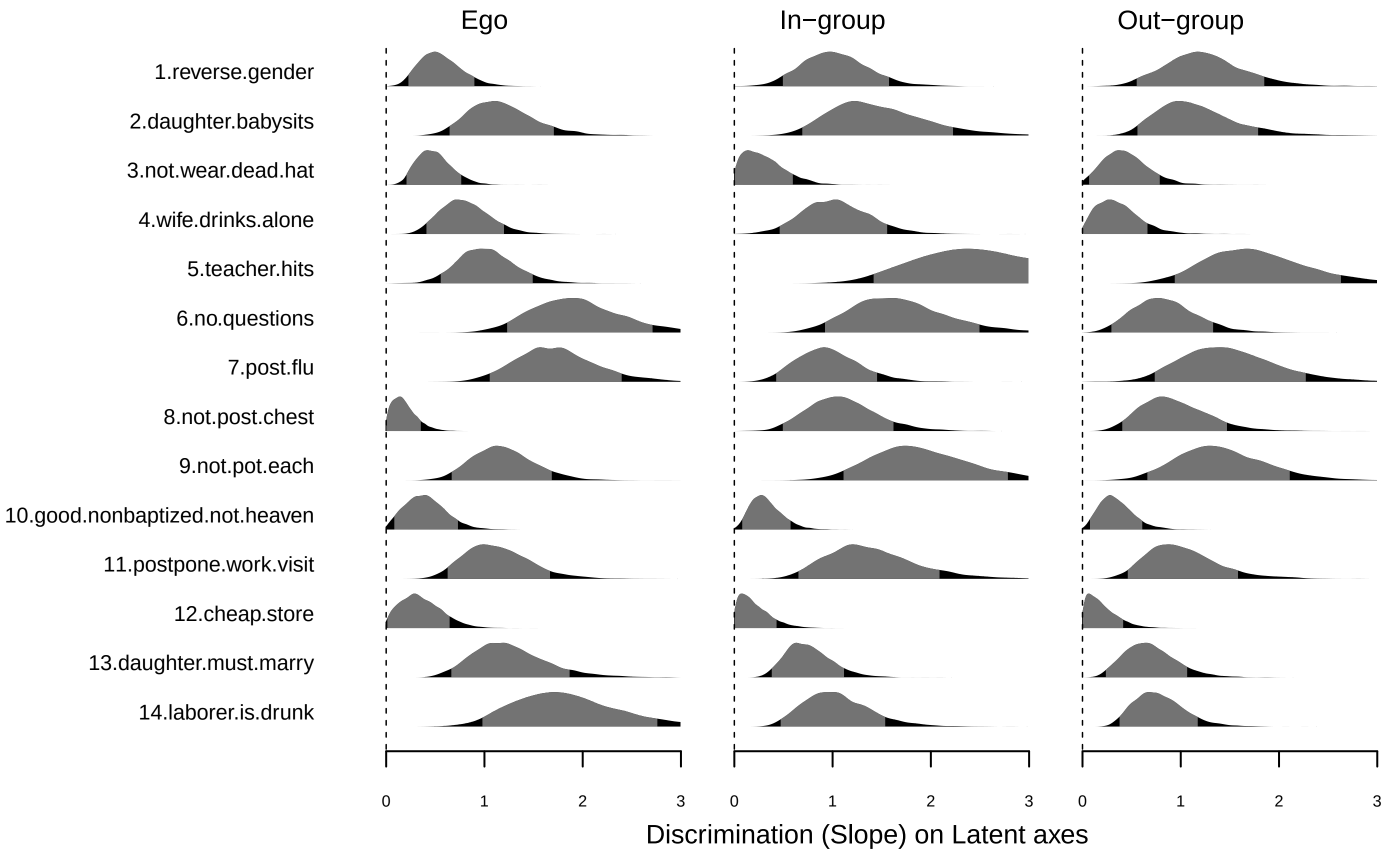

Figure S7: Question discrimination estimates. Posterior probability density estimates for discrimination parameters (γ) for each question k on each latent target axis t (ego, in-group, and out-group) from an IRT model with a random effect for individual and no fixed effect predictors (m1 in Tables S1 and S2 below). 90% highest posterior density intervals (HPDI) are shown in grey. Note that all discrimination estimates are markedly non-zero, with the exception of those for questions 8 and 12 on the ego axis, 3 and 12 on the in-group axis, and 4 and 12 on the out-group axis, which are marginally non-zero. This suggests that most questions contribute substantially to the construction of the latent axes, and supports the decision to limit analysis to a single latent dimension (Jackman, 2001). The coding of question 12 on the in-group axis, and questions 8, 12, and 13 on the out-group axis, are reversed from that for the ego axis.

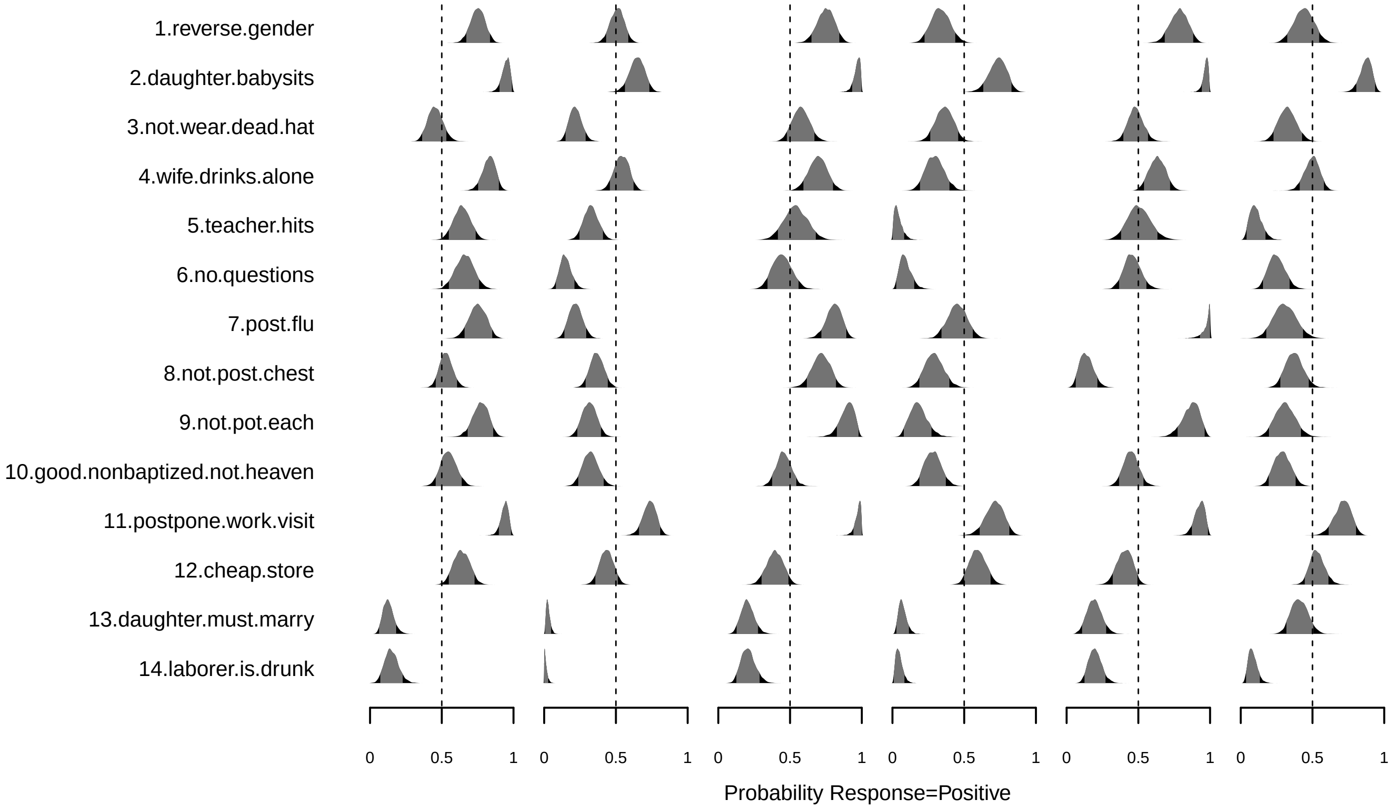

Figure S8: Posterior distributions of response probabilities. Distributions of probabilities of giving the positive response for each of the 14 vignette questions, by ethnicity and target (ego, in-group, and out-group), derived from posterior predictions of the IRT model m4, shown in Tables S1 and S2. 90% highest posterior density intervals (HPDI) are shown in grey. Vertical lines at 0.5 are provided for visual guidance. Coding of questions has been standardized across targets. Note that, on average, for most questions, for all three targets, Matsigenka and Mestizos tended to answer differently.

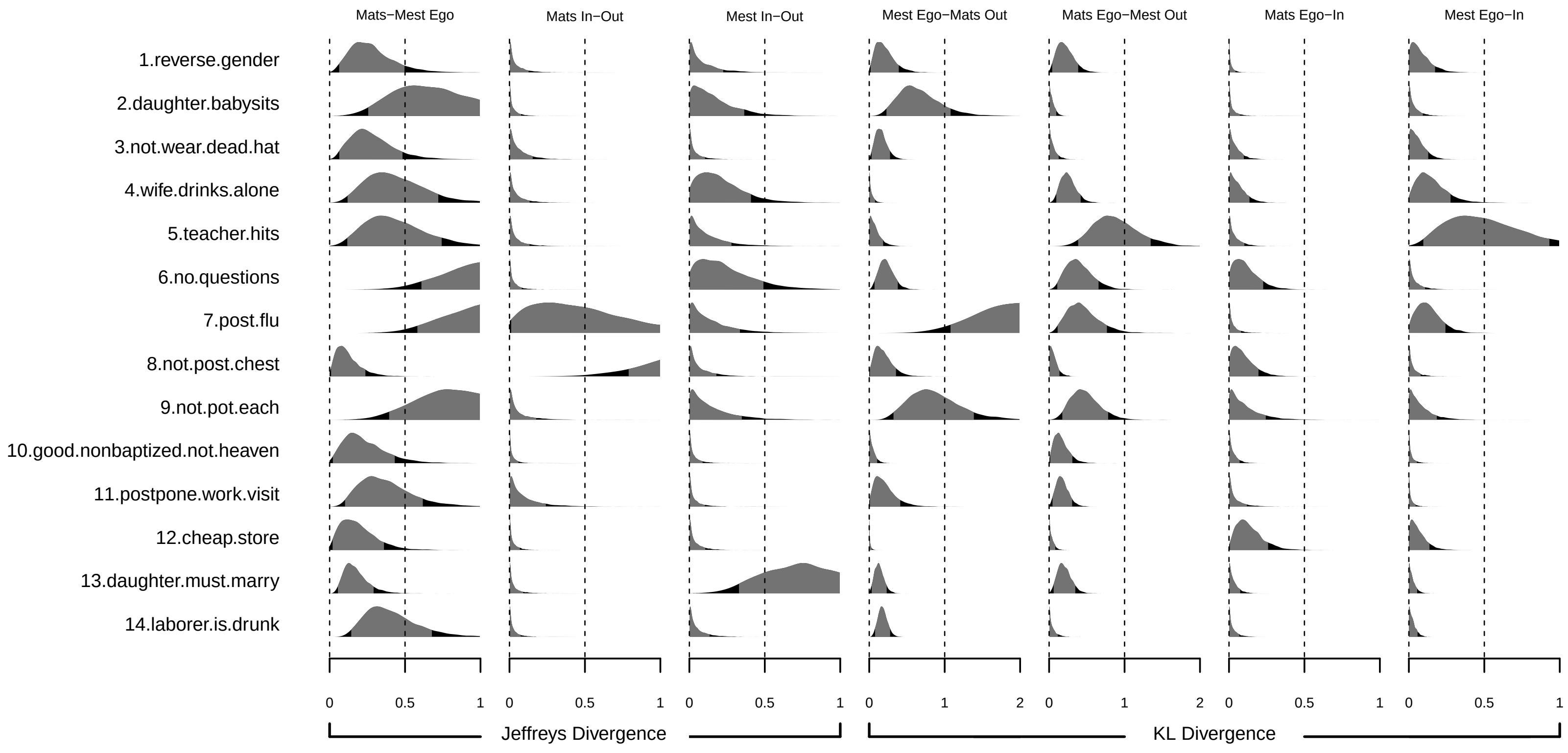

Figure S9: (Caption next page.)

Figure S9: (Previous page) Posterior distributions of response divergences. Distributions of divergences among probability distributions for the responses of average Matsigenka and Mestizos for each of the 14 vignette questions. First column: Jeffreys divergence (Kullback and Leibler, 1951; Jeffreys, 1948) among the probabilities that average Matsigenka and Mestizos gave the positive ego response for each question (Mats Ego and Mest Ego, respectively). This represents how differently Matsigenka and Mestizos tended to answer each question. Second column: Jeffreys divergence between the probability that average Matsigenka guessed that average Matsigenka gave the positive ego response (Mats In) and the probability that average Matsigenka guessed that average Mestizos gave the positive ego response (Mats Out). This represents how differently Matsigenka perceive themselves to be from Mestizos. Third column: Jeffreys divergence between the probability that average Mestizos guessed that average Mestizos gave the positive ego response (Mest In) and the probability that average Mestizos guessed that average Matsigenka gave the positive ego response (Mest Out). This represents how differently Mestizos perceive themselves to be from Matsigenka. Fourth column: Kullback-Leiber (K-L) divergence (Kullback and Leibler, 1951; McElreath, 2016) between the probability that the average Mestizo gave the positive ego response (Mest Ego, the K-L "target") and the probability that the average Matsigenka guessed that the average Mestizo gave the positive ego response (Mats Out, the K-L "approximation"). This represents the inaccuracy of Matsigenka out-group guesses. Fifth column: K-L divergence between Matsigenka ego responses (Mats Ego, K-L target) and Mestizo out-group guesses (Mest Out, K-L approximation). This represents the inaccuracy of Mestizo out-group guesses. Sixth column: K-L divergence between Matsigenka ego responses (Mats Ego, K-L target) and Matsigenka in-group guesses (Mats In, K-L approximation). This represents the inaccuracy of Matsigenka in-group guesses. Seventh column: K-L divergence between Mestizo ego responses (Mest Ego, K-L target) and Mestizo in-group guesses (Mest In, K-L approximation). This represents the inaccuracy of Mestizo in-group guesses. Distributions are derived from posterior predictions of the IRT model m4, shown in Tables S1 and S2. 90% highest posterior density intervals (HPDI) are shown in grey. Vertical lines at 0 are provided for visual guidance. Coding of questions has been standardized across targets. Note that Matsigenka tended to believe that Mestizos answered question 8 very differently from how they believed their co-ethnics answered the question (column 2). For Mestizos, the same is true for question 13 (column 3). This indicates that interviewees understood the nature of the guessing interviews. The inaccuracy of Matsigenka out-group guesses is driven primarily by questions 2, 7, and 9 (column 4), and for Mestizos by questions 5, 6, 7, and 9 (column 5). The inaccuracy of Matsigenka in-group guesses is driven primarily by questions 6, 8, and 12 (column 6), and for Mestizos by questions 4, 5, and 7 (column 7).

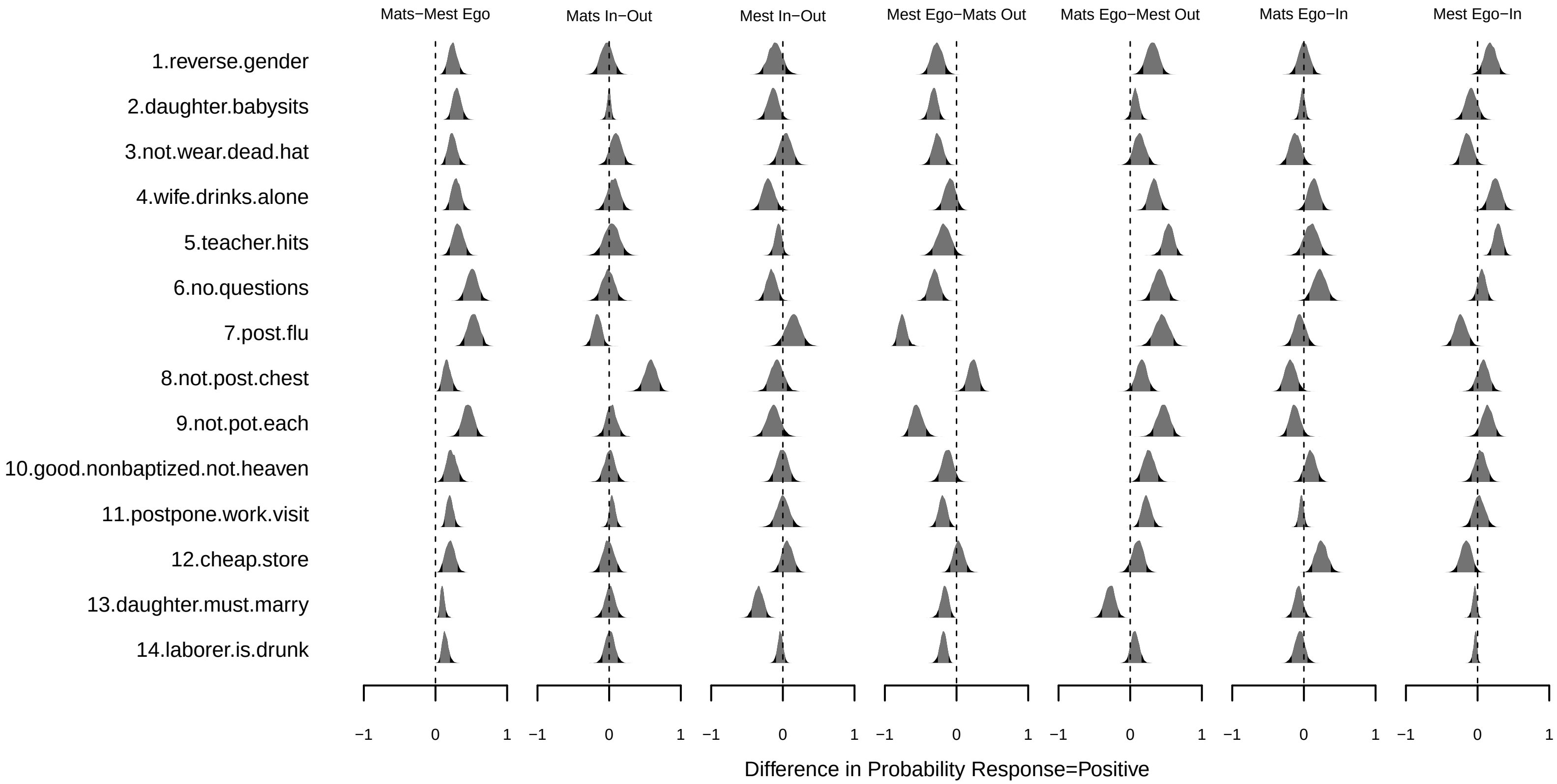

Figure S10: Posterior distributions of response contrasts. Distributions of the contrasts (differences) in probabilities of giving the positive response for each of the 14 vignette questions. Column headings and HPDI match those of Figure S9. Unlike divergences, simple subtraction preserves the direction of the differences between probability distributions. Note that Matsigenka and Mestizos generally believed that the out-group and the in-group answered the vignette questions in similar ways, with the exception of question 8 for Matsigenka (column 2) and question 13 for Mestizos (column 3). This corresponds with the fact that out-group inaccuracies of the two groups broadly mirror each other (columns 4 and 5), except for questions 8 and 13. Thus, the model predicts that Matsigenka and Mestizos tend to make similarly inaccurate guesses about the out-group for most questions, guessing that the out-group and the in-group answered in the same way, when in fact this was generally not the case (column 1).

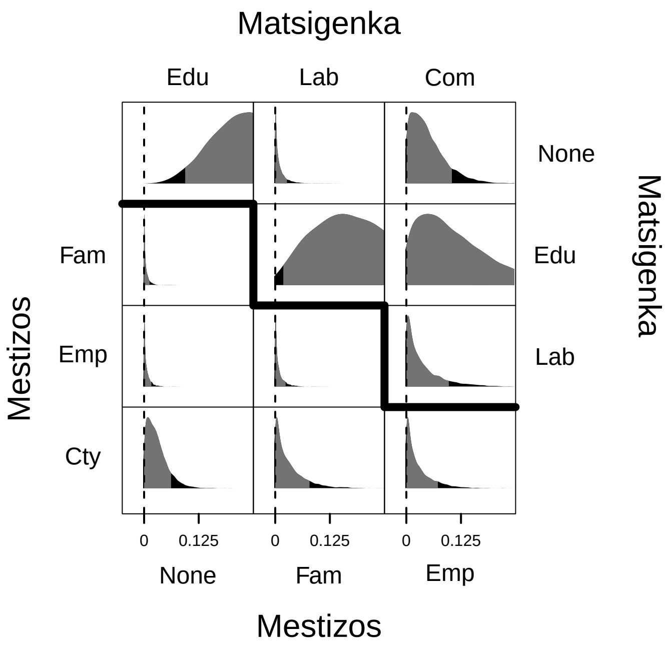

Figure S11: Distributions of divergence for personally-held norms. Distributions of mean (across questions) Jeffreys divergence between the probabilities of giving the positive ego response, for Matsigenka and Mestizos with various types of counter-factual inter-ethnic experience. This corresponds to column 1 of Fig 3. Matsigenka divergences are derived from posterior predictions of response probabilities from the IRT model m11, and Mestizo divergences are derived from model m19 (Tables S1 and S2). 90% highest posterior density intervals (HPDI) are shown in grey. Vertical lines at 0 are provided for visual guidance. Inter-ethnic experience types are: Matsigenka who have experience with Mestizos in the domains of education (Edu), wage labor worker (Lab), commerce (Com), or none of these domains (None); Mestizos who have experience with Matsigenka in the domains of family (Fam), wage labor employer (Emp), community participation (Cty), or none of these domains (see Section Appendix A.3.3). Note that, for Matsigenka with education experience, the average probability of giving a positive response tends to diverge greatly from that of their co-ethnics (upper triangle of matrix). For Mestizos who lived in a Matsigenka community, the average probability of giving the positive response tends to diverge slightly from that of their co-ethnics (lower triangle of matrix).

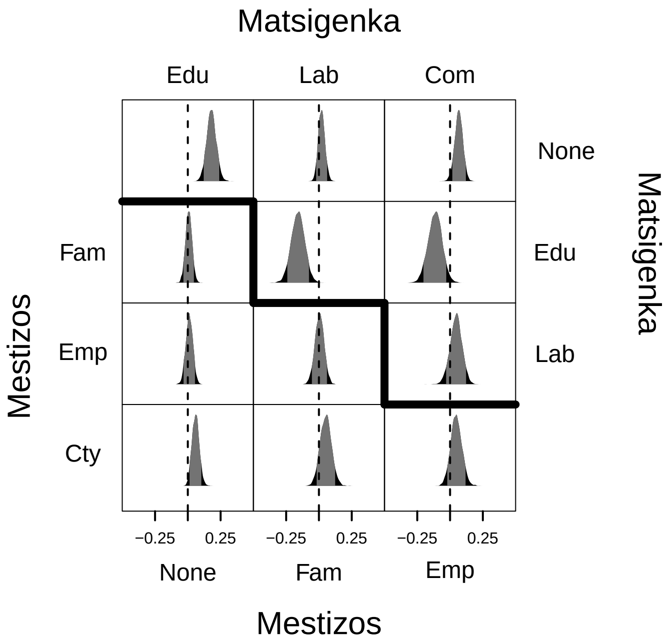

Figure S12: Distributions of contrasts for personally-held norms. Distributions of mean (across questions) contrasts between the probabilities of giving the positive ego response, for Matsigenka and Mestizos with various types of counter-factual inter-ethnic experience. Inter-ethnic experience labels and HPDI match those of Figure S11. All contrasts are computed as row minus column. Unlike divergences, simple subtraction preserves the direction of the differences between probability distributions. Trends match those of Figure S11.

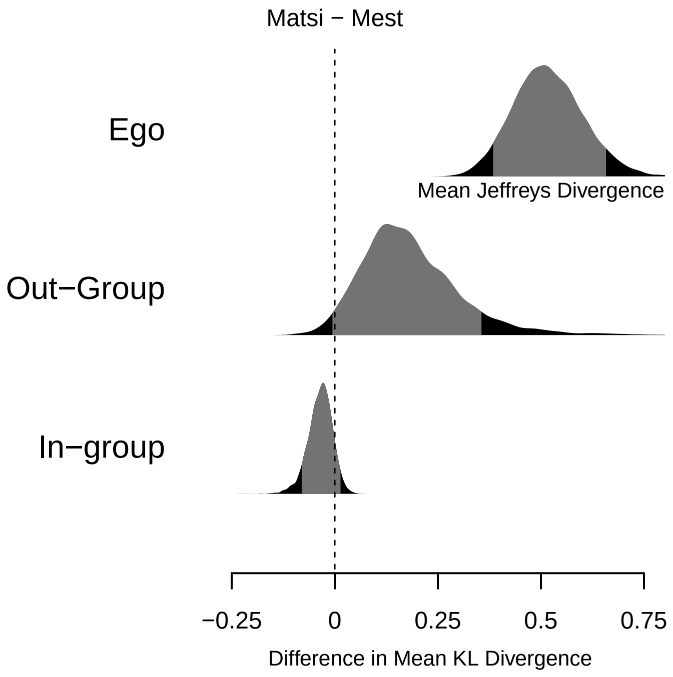

Figure S13: Ethnicity-level distributions of response divergence. Distributions of the mean (across questions) Jeffreys divergence between the probabilities than an average Matsigenka and an average Mestizos gave the positive ego response (top), and contrasts of mean (across questions) inaccuracy of out-group guesses (i.e., K-L divergence between out-group ego response and guess about out-group ego response) and in-group guesses between Matsigenka and Mestizos. Contrasts (middle and bottom) are calculated as mean Matsigenka K-L divergence minus mean Mestizo K-L divergence. This corresponds with columns 2 and 3 of Fig 3. Note that, on average, the probabilities of Matsigenka and Mestizo guesses tend to diverge (top), and Matsigenka tend to have more inaccurate out-group guesses (middle), but less inaccurate in-group guesses (bottom) than Mestizos.

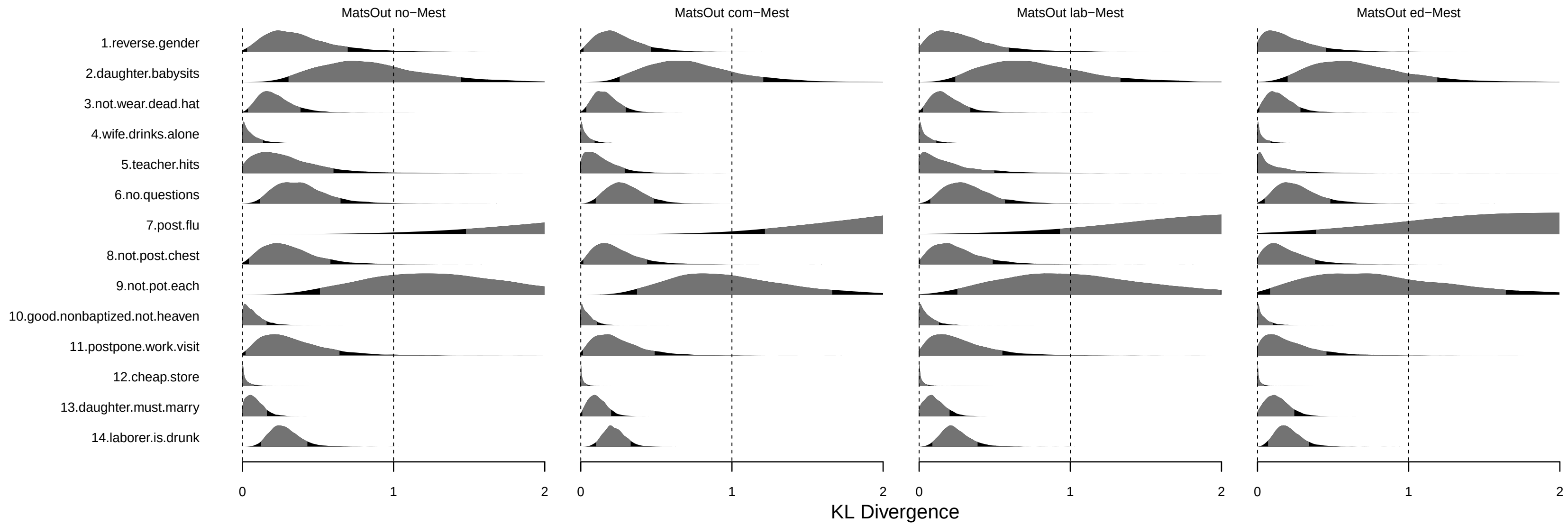

Figure S14: Distributions of Matsigenka out-group guess inaccuracy. Distributions of Kullback-Leibler (K-L) divergence between the probability that an average Mestizo gives the positive ego response (K-L ”target”) and the probabilities that average Matsigenka with various types of counter-factual inter-ethnic experience guess that an average Mestizo gives the positive ego response (K-L ”approximation”). These represent the inaccuracies of Matsigenka out-group guesses for each of the 14 vignette questions. First column: Matsigenka with no inter-ethnic experience. Second column: Matsigenka with commerce experience. Third column: Matsigenka with wage labor worker experience. Fourth column: Matsigenka with education experience (see Section Appendix A.3.3). K-L divergences are computed using posterior probabilities from the IRT model m11, shown in Table S1. 90% highest posterior density intervals (HPDI) are shown in grey. Vertical lines at 0 and 0.5 are provided for visual guidance. Coding of questions has been standardized across targets. Note that Matsigenka with inter-ethnic education experience (column 4) generally have lower inaccuracy than Matsigenka without inter-ethnic experience (column 1), particularly with regard to out-group guesses for questions 5, 7, and 9 (see Table 1).

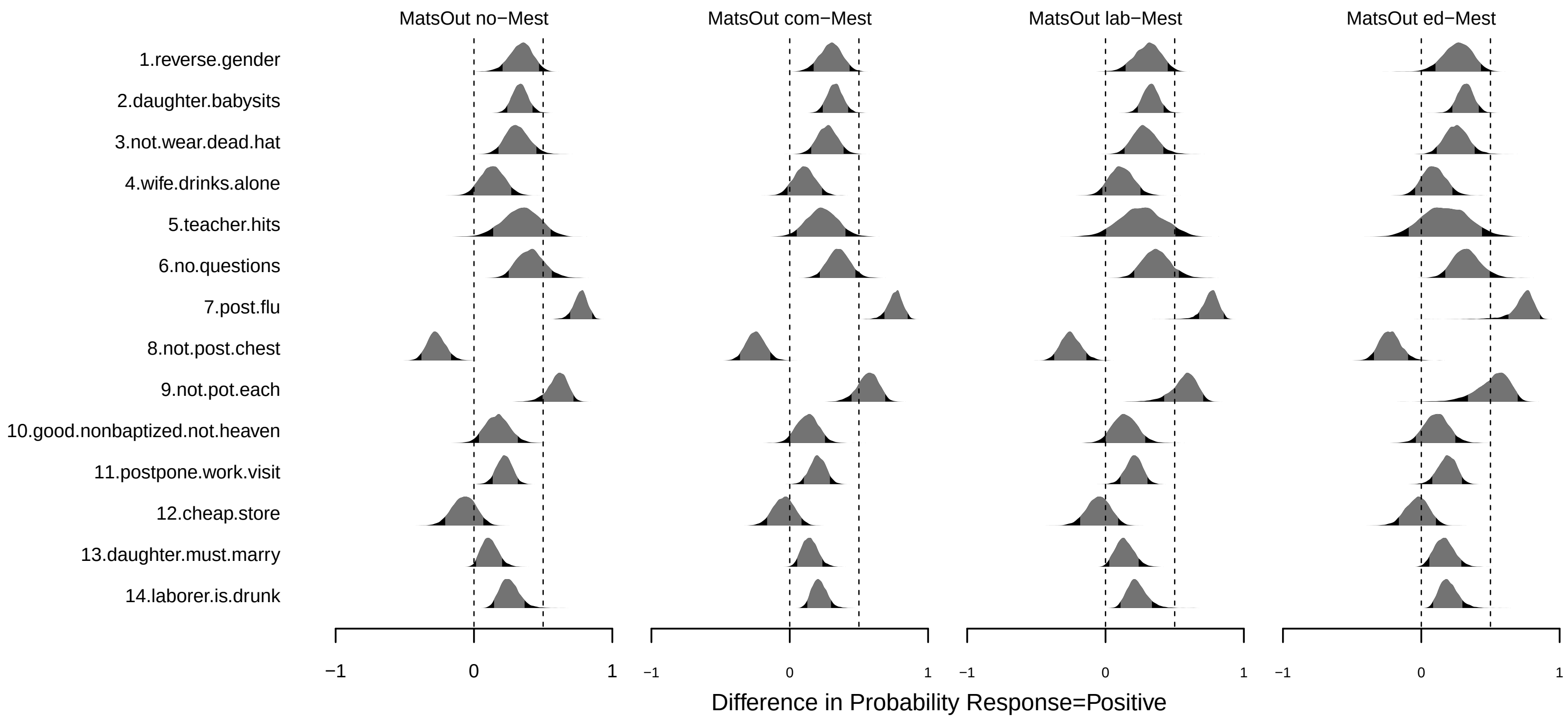

Figure S15: Distributions of Matsigenka out-group guess contrasts. Distributions of the contrasts (differences) in probabilities of giving the positive response for each of the 14 vignette questions. Column headings and HPDI match those of Figure S14. Unlike divergences, simple subtraction preserves the direction of the differences between probability distributions. All contrasts are computed as Matsigenka out-group guess minus Mestizo ego response. Trends match those of Figure S14.

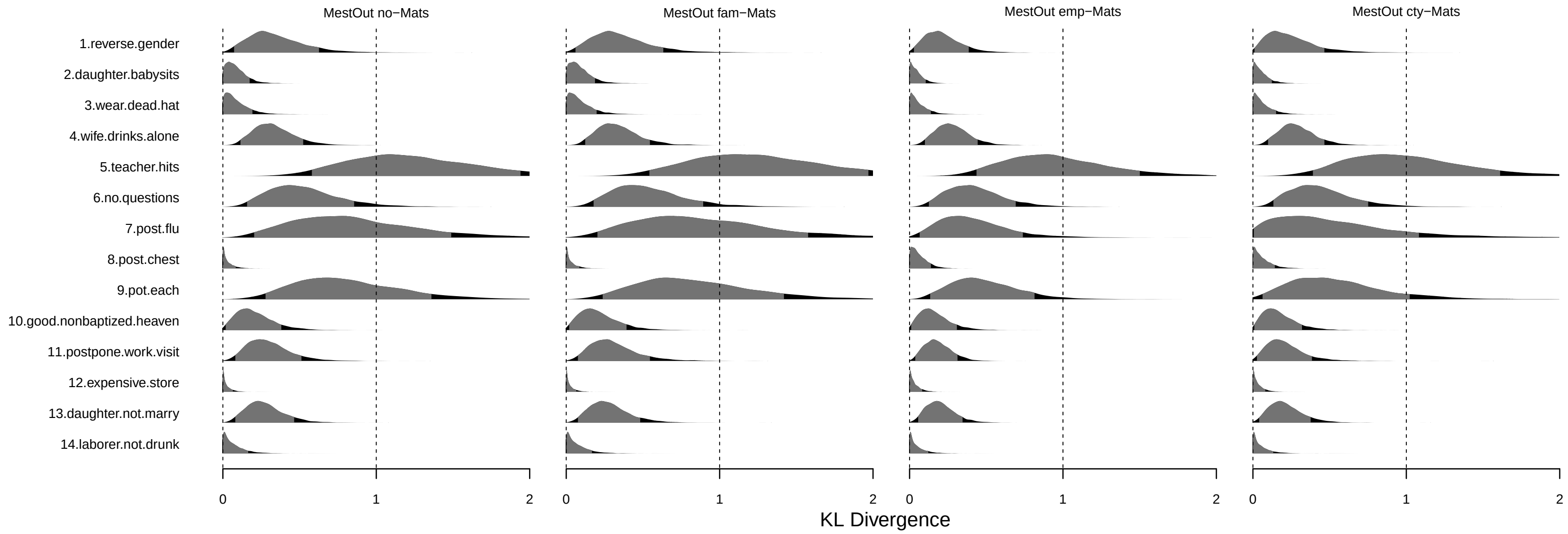

Figure S16: Distributions of Mestizo out-group guess inaccuracy. Distributions of Kullback-Leibler (K-L) divergence between the probability that an average Matsigenka gives the positive ego response (K-L "target") and the probabilities that average Mestizos with various types of counter-factual inter-ethnic experience guess that an average Matsigenka gives the positive ego response (K-L "approximation"). These represent the inaccuracies of Mestizo out-group guesses for each of the 14 vignette questions. First column: Mestizos with no inter-ethnic experience. Second column: Mestizos with indigenous family experience. Third column: Mestizos with wage labor employer experience. Fourth column: Mestizos with indigenous community experience (see Section Appendix A.3.3). K-L divergences are computed using posterior probabilities from the IRT model m19, shown in Table S2. 90% highest posterior density intervals (HPDI) are shown in grey. Vertical lines at 0 and 0.5 are provided for visual guidance. Coding of questions has been standardized across targets. Note that Mestizos with inter-ethnic employer and community experience (columns 3 and 4) generally have lower inaccuracy than Mestizos with no inter-ethnic experience and family experience (columns 1 and 2), particularly with regard to out-group guesses for questions 5, 7, and 9 (see Table 1).

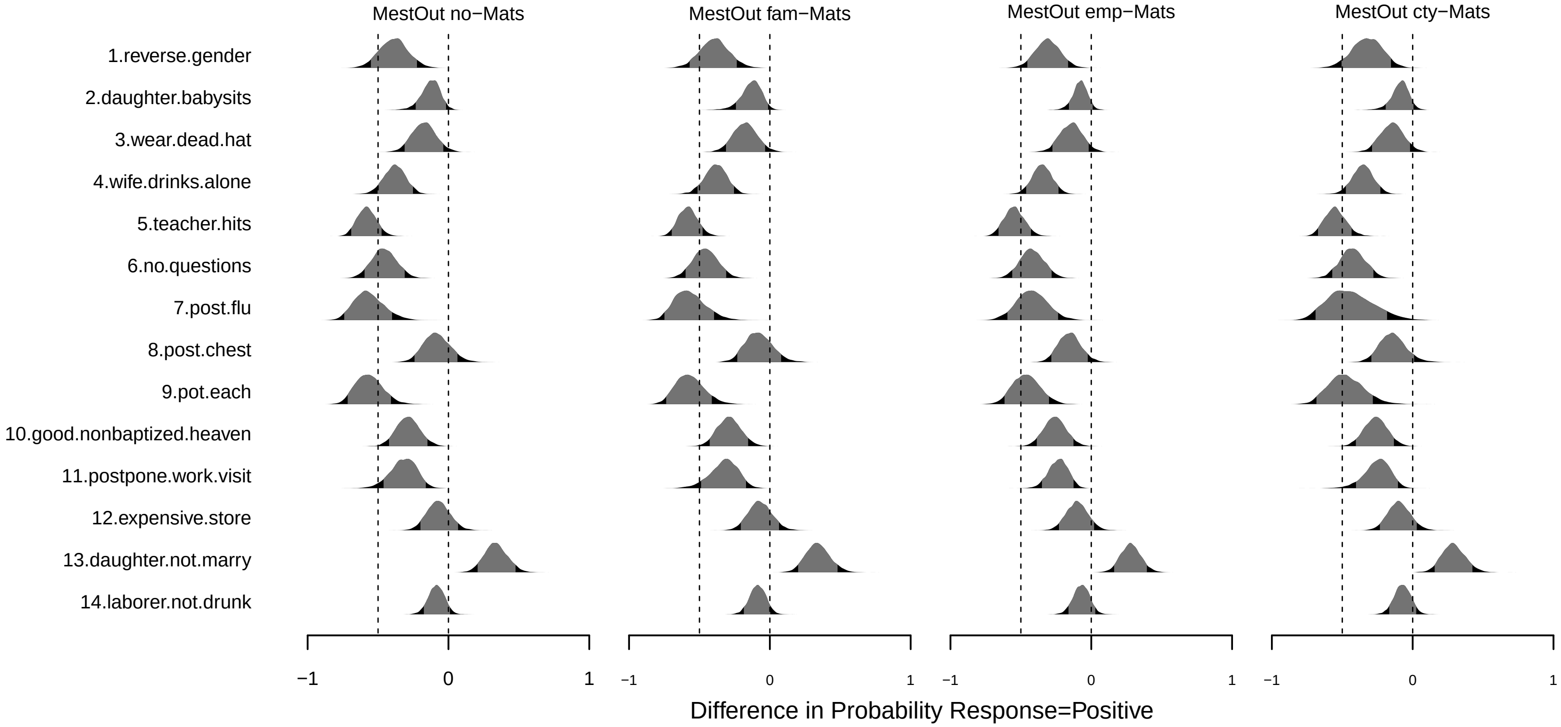

Figure S17: Distributions of Mestizo out-group guess contrasts. Distributions of the contrasts (differences) in probabilities of giving the positive response for each of the 14 vignette questions. Column headings and HPDI match those of Figure S16. Unlike divergences, simple subtraction preserves the direction of the differences between probability distributions. All contrasts are computed as Mestizo out-group guess minus Matsigenka ego response. Trends match those of Figure S16.

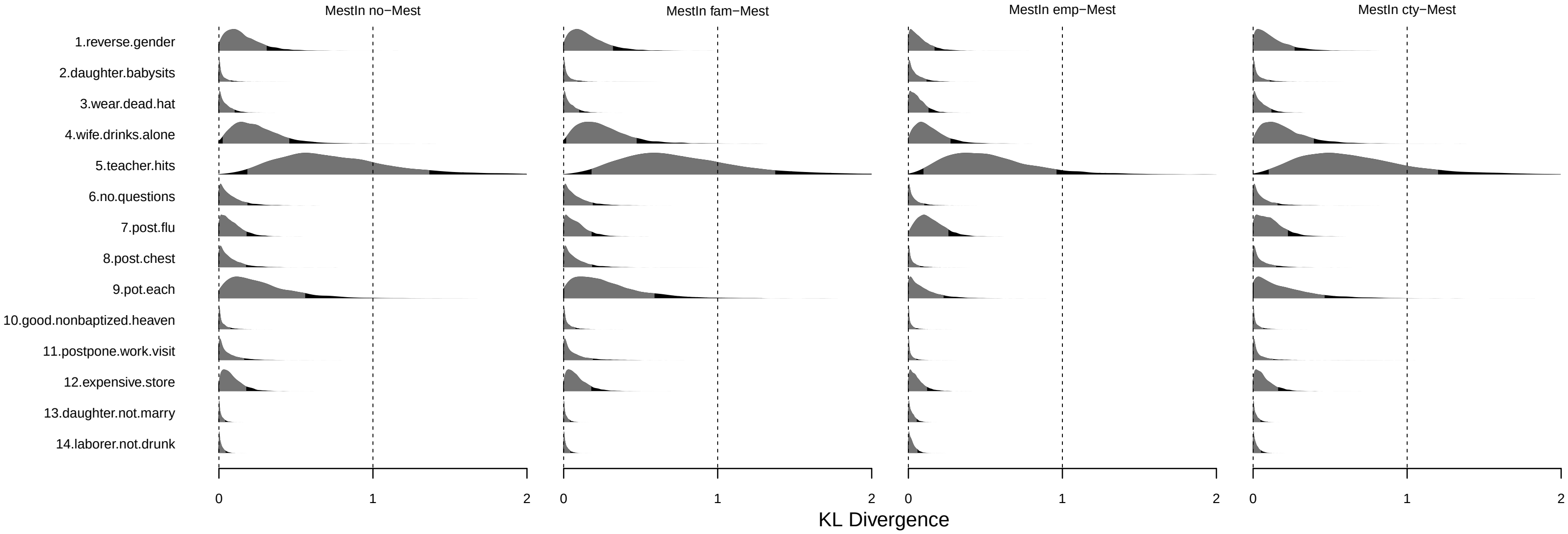

Figure S18: Distributions of Mestizo in-group guess inaccuracy. Distributions of Kullback-Leibler (K-L) divergence between the probability that an average Mestizo gives the positive ego response (K-L ”target”) and the probabilities that average Mestizos with various types of counter-factual inter-ethnic experience guess that an average Mestizo gives the positive ego response (K-L ”approximation”). These represent the inaccuracies of Mestizo in-group guesses for each of the 14 vignette questions. Column headings match those of Figure S16. K-L divergences are computed using posterior probabilities from the IRT model m19, shown in Table S2. 90% highest posterior density intervals (HPDI) are shown in grey. Vertical lines at 0 and 0.5 are provided for visual guidance. Coding of questions has been standardized across targets. Note that Mestizos with inter-ethnic employer experience (column 3) have lower inaccuracy than Mestizos with no inter-ethnic experience and family experience (columns 1 and 2), particularly with regard to in-group guesses for questions 8, 9, and 11 (see Table 1).

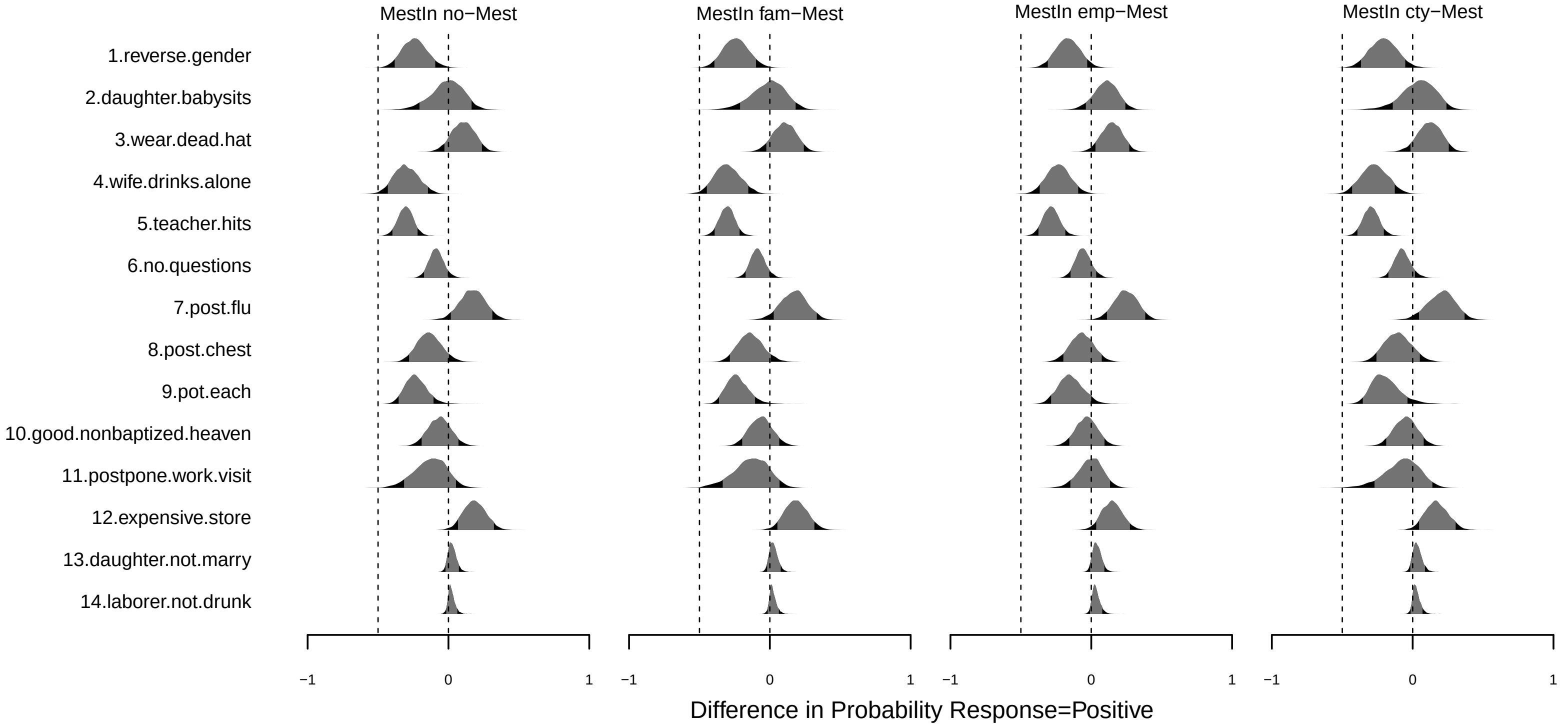

Figure S19: Distributions of Mestizo in-group guess contrasts. Distributions of the contrasts (differences) in probabilities of giving the positive response for each of the 14 vignette questions. Column headings and HPDI match those of Figure S18. Unlike divergences, simple subtraction preserves the direction of the differences between probability distributions. All contrasts are computed as Mestizo in-group guess minus Mestizo ego response. Trends match those of Figure S18.

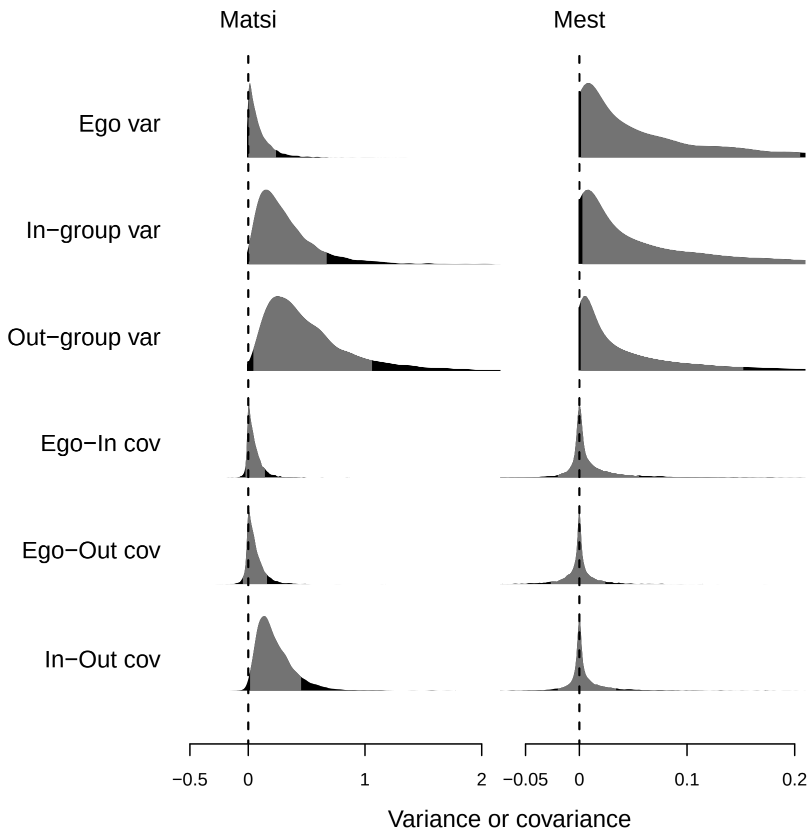

Figure S20: Variance and covariance among personally-held norms and guesses. Residual variance in individual location on the three latent target axes (ego, in-group, and out-group), and covariances in individual locations between target axes, derived from posterior predictions for the **S** matrices in Eq A.7. The first column comprises the components of $\mathbf{S}_{Matsigenka}$ from model m11, and second column comprises the components of $\mathbf{S}_{Mestizo}$ from model m19 (Tables S1 and S2). 90% highest posterior density intervals (HPDI) are shown in grey. Vertical lines at 0 are provided for visual guidance. Note that the scale of the axis for Mestizo variance and covariance is an order of magnitude smaller than that for Matsigenka.

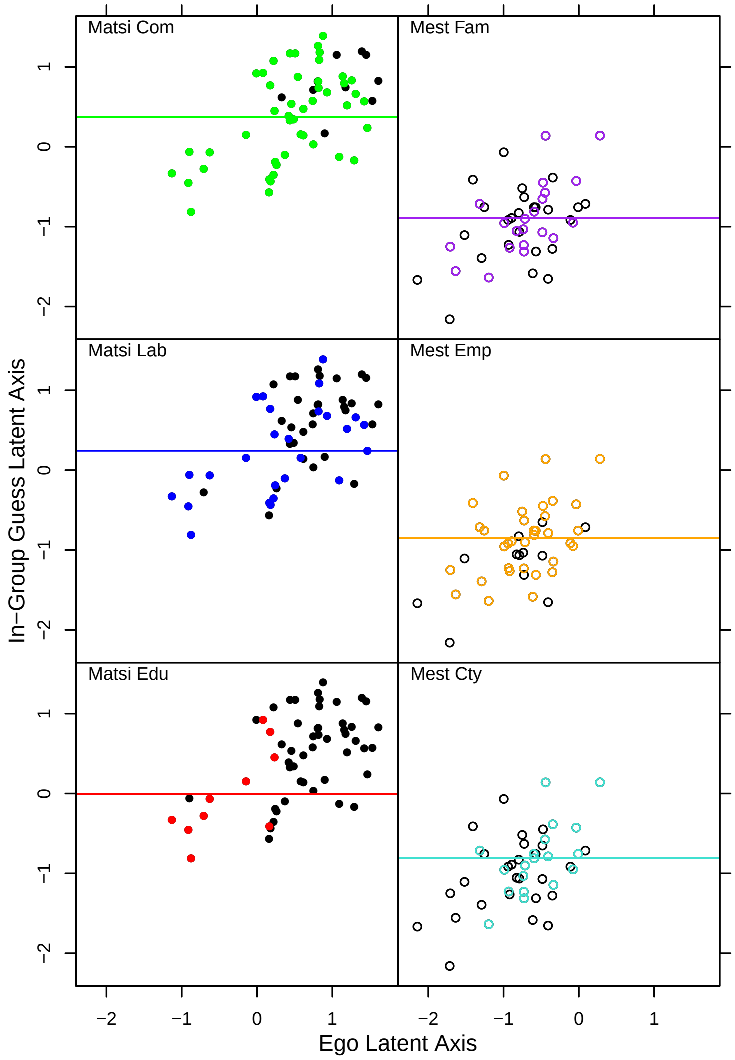

Figure S21: Individual locations on ego and in-group latent axes. Individual locations on the latent in-group axis (y) against individual locations on the latent ego axis (x) for Matsigenka (first column) and Mestizos (second column) by different types of inter-ethnic experience (labels follow Figure S11). Points are means for each individual derived from posterior predictions of model m1, with no estimated covariance among individual locations across target axes and no inter-ethnic experience predictors (Tables S1 and S2). Horizontal lines are drawn at the mean location on the in-group axis for individuals with the given inter-ethnic experience type. Note the positive overall covariance, and residual covariance within each experience type for the Matsigenka.

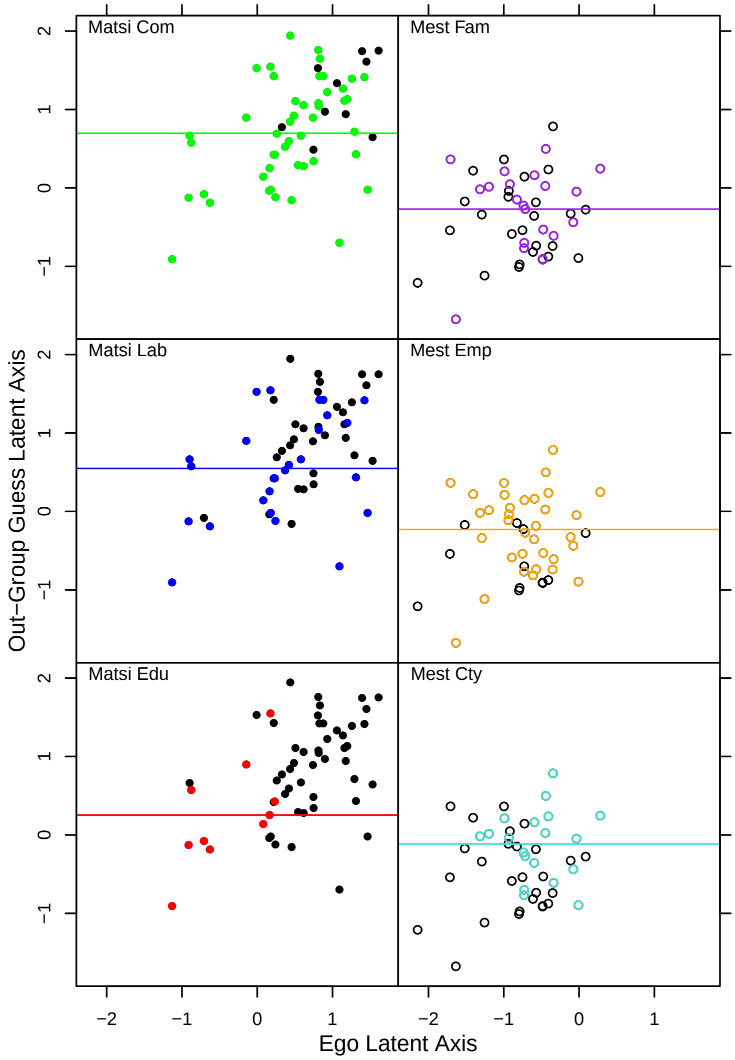

Figure S22: Individual locations on ego and out-group latent axes. Individual locations on the latent out-group axis (y) against individual locations on the latent ego axis (x) for Matsigenka (first column) and Mestizos (second column) by different types of inter-ethnic experience (labels follow Figure S11). Points are means for each individual derived from posterior predictions of model m1, with no estimated covariance among individual locations across target axes and no inter-ethnic experience predictors (Tables S1 and S2). Horizontal lines are drawn at the mean location on the out-group axis for individuals with the given inter-ethnic experience type. Note the positive overall covariance, and residual covariance within each experience type for the Matsigenka.

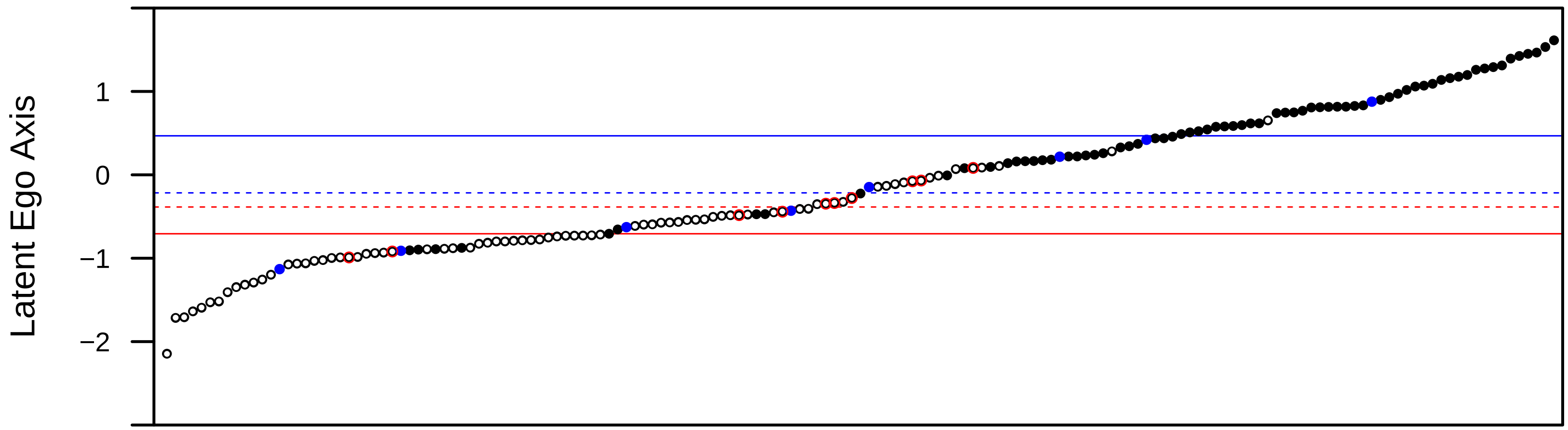

Figure S23: Locations of familiar individuals on ego axis. Individual locations on the latent ego axis for Matsigenka (solid circles) and Mestizos (open circles). Points are means for each individual derived from posterior predictions of model m1, with no estimated covariance among individual locations across target axes and no inter-ethnic experience predictors (Tables S1 and S2). Solid horizontal lines are drawn at the mean locations for Matsigenka (blue) and Mestizos (red). Blue points are Matsigenka individuals most familiar to Mestizos in Boca Manu and Atalaya, derived from my ethnographic observations. The dashed blue line is the mean location of these individuals. Red points are Mestizo individuals most familiar to Matsigenka in Tayakome, derived from my ethnographic observations. The dashed red line is the mean location of these individuals. Note that Matsigenka individuals most familiar to Mestizos are different on average from the average Matsigenka. Mestizo individuals most familiar to Matsigenka are more similar on average to the average Mestizo.

Table S1: (Next page) IRT models for Matsigenka inter-ethnic experience. Posterior mean coefficient estimates for IRT models fit in this analysis (with 90% highest probability density interval in parentheses). Models receiving more than 1% WAIC weight are highlighted in grey. The model used for inference, m11, incorporates all Matsigenka inter-ethnic experience domains of interest, without the exploratory predictors of sex and age. Column headings are predictors and model weight. Adol = Adolescent, Educ = Education experience with Mestizos, Labor = wage labor worker experience with Mestizos, Comm = commerce experience with Mestizos. For m1 and m3, rows are intercepts on the target latent axes (from top to bottom: ego, in-group, and out-group). m1 has a random effect for individual and no covariance estimation. m2 (not shown) is the standard centered parameterization of the model in equations A.3-A.17, and could not be well-estimated. m3 (and all subsequent models) adds covariance among intercepts across targets and among question locations and discriminations within and across targets, and uses the non-centered Cholesky-factored parameterization (along with all subsequent models). Rows of m4 - m12 are, from top to bottom, Matsigenka ego, in-group, out-group, and Mestizo ego, in-group, and out-group estimates.

Table S1: (Caption previous page)

Model	Intercept	Sex	Adol	Elder	Educ	Labor	Comm	WAIC weight
m1	-0.13 (-0.77, 0.49)							1.2e-44
	-0.17 (-0.84, 0.48)							
	0.28 (-0.44, 0.98)							
m3	-0.08 (-1.35, 1.1)							1.5e-11
	-0.08 (-1.35, 1.1)							
	-0.08 (-1.35, 1.1)							
m4	0.69 (-0.34, 1.81)							1.2e-05
	0.68 (-0.35, 1.75)							
	0.7 (-0.41, 1.67)							
	-0.78 (-1.91, 0.26)							
	-0.89 (-1.93, 0.25)							
	-0.57 (-1.59, 0.45)							
m5	0.69 (-0.42, 1.81)				-1.2 (-1.89, -0.58)			0.36
	0.67 (-0.48, 1.82)				-0.63 (-1.14, -0.15)			
	0.7 (-0.43, 1.84)				-0.65 (-1.18, -0.09)			
	-0.52 (-1.87, 0.78)				-0.52 (-1.83, 0.79)			
	-0.65 (-2, 0.64)				-0.65 (-1.9, 0.74)			
	-0.42 (-1.67, 0.93)				-0.42 (-1.71, 0.87)			
m6	0.77 (-0.45, 1.9)					-0.63 (-1.04, -0.18)		3.8e-05
	0.72 (-0.44, 1.86)					-0.49 (-0.91, -0.1)		
	0.75 (-0.5, 1.85)					-0.47 (-0.9, -0.05)		
	-0.58 (-1.9, 0.71)					-0.58 (-1.84, 0.75)		
	-0.69 (-2.06, 0.6)					-0.66 (-1.99, 0.66)		
	-0.44 (-1.79, 0.86)					-0.44 (-1.78, 0.9)		
m7	0.87 (-0.28, 2.04)						-0.7 (-1.15, -0.2)	2.9e-05
	0.81 (-0.34, 2.02)						-0.48 (-0.97, 0)	
	0.83 (-0.3, 2.07)						-0.49 (-1.03, 0.05)	
	-0.59 (-1.9, 0.75)						-0.59 (-1.95, 0.7)	
	-0.67 (-1.97, 0.69)						-0.67 (-1.94, 0.67)	
	-0.46 (-1.75, 0.84)						-0.45 (-1.78, 0.82)	
m8	0.75 (-0.42, 1.87)				-1.25 (-1.9, -0.57)	-0.24 (-0.55, 0.09)		0.052
	0.74 (-0.37, 2)				-0.53 (-1.09, 0.05)	-0.4 (-0.85, 0.04)		
	0.78 (-0.34, 1.96)				-0.55 (-1.17, 0)	-0.35 (-0.84, 0.1)		
	-0.45 (-1.93, 0.93)				-0.45 (-1.88, 0.96)	-0.44 (-1.82, 0.93)		
	-0.56 (-1.99, 0.84)				-0.56 (-2.03, 0.84)	-0.55 (-2.02, 0.86)		
	-0.36 (-1.72, 1.13)				-0.35 (-1.78, 1.01)	-0.36 (-1.7, 1.05)		
m9	0.85 (-0.32, 2.07)				-1.18 (-1.79, -0.57)		-0.47 (-0.83, -0.07)	0.42
	0.83 (-0.34, 2.06)				-0.61 (-1.13, -0.07)		-0.44 (-0.99, 0.06)	
	0.86 (-0.39, 2.05)				-0.6 (-1.13, -0.05)		-0.45 (-1.06, 0.11)	
	-0.47 (-1.91, 0.93)				-0.46 (-1.86, 0.98)		-0.46 (-1.77, 1)	
	-0.56 (-2.02, 0.87)				-0.56 (-1.94, 0.88)		-0.55 (-1.99, 0.86)	
	-0.38 (-1.69, 1.17)				-0.36 (-1.74, 1.01)		-0.39 (-1.84, 0.99)	
m10	0.94 (-0.24, 2.14)					-0.53 (-0.94, -0.12)	-0.6 (-1.06, -0.08)	2.7e-05
	0.8 (-0.5, 1.94)					-0.45 (-0.91, 0)	-0.32 (-0.87, 0.25)	
	0.87 (-0.33, 2.11)					-0.41 (-0.88, 0.06)	-0.36 (-1.02, 0.24)	
	-0.5 (-1.9, 0.93)					-0.52 (-1.94, 0.9)	-0.5 (-1.94, 0.83)	
	-0.58 (-1.99, 0.83)					-0.58 (-2.09, 0.72)	-0.56 (-1.98, 0.83)	
	-0.39 (-1.76, 1.08)					-0.37 (-1.74, 1.05)	-0.38 (-1.79, 0.98)	
m11	0.92 (-0.27, 2.14)				-1.27 (-1.94, -0.62)	-0.16 (-0.48, 0.18)	-0.49 (-0.97, -0.07)	0.17
	0.84 (-0.37, 2.1)				-0.54 (-1.12, 0.03)	-0.34 (-0.83, 0.16)	-0.33 (-0.91, 0.26)	
	0.89 (-0.33, 2.15)				-0.56 (-1.15, 0)	-0.27 (-0.81, 0.22)	-0.36 (-1.01, 0.32)	
	-0.4 (-1.98, 0.9)				-0.41 (-1.79, 1.15)	-0.41 (-1.91, 1)	-0.43 (-1.87, 1.01)	
	-0.51 (-1.97, 1.01)				-0.49 (-1.96, 1.02)	-0.49 (-1.98, 0.93)	-0.48 (-1.97, 0.98)	
	-0.34 (-1.75, 1.07)				-0.32 (-1.75, 1.14)	-0.33 (-1.77, 1.08)	-0.33 (-1.77, 1.13)	
m12	1.23 (0.04, 2.49)	0.17 (-0.29, 0.62)	-0.28 (-1.01, 0.4)	-0.23 (-0.79, 0.33)	-1.66 (-2.37, -0.94)	-0.33 (-0.84, 0.22)	-0.69 (-1.25, -0.14)	3e-06
	1.15 (-0.13, 2.39)	-0.15 (-0.89, 0.52)	-0.25 (-1.22, 0.7)	-0.54 (-1.35, 0.27)	-0.68 (-1.44, 0.02)	-0.38 (-1.1, 0.4)	-0.42 (-1.14, 0.35)	
	1.19 (-0.05, 2.46)	0.09 (-0.62, 0.87)	-0.4 (-1.42, 0.59)	-0.6 (-1.41, 0.32)	-0.67 (-1.44, 0.06)	-0.47 (-1.31, 0.29)	-0.44 (-1.23, 0.43)	
	-0.6 (-2.15, 0.87)	0.08 (-0.33, 0.49)	0.37 (-0.62, 1.38)	0.04 (-0.43, 0.52)	-0.58 (-2.11, 0.84)	-0.58 (-2.1, 0.82)	-0.6 (-1.99, 0.92)	
	-0.66 (-2.04, 0.87)	-0.26 (-0.8, 0.29)	0.43 (-0.74, 1.69)	-0.02 (-0.56, 0.6)	-0.67 (-2.16, 0.81)	-0.67 (-2.2, 0.74)	-0.68 (-2.25, 0.71)	
	-0.45 (-1.95, 1)	-0.31 (-0.77, 0.1)	-0.42 (-1.47, 0.71)	0.08 (-0.37, 0.51)	-0.45 (-1.86, 1.08)	-0.44 (-1.93, 0.99)	-0.42 (-1.93, 1.07)	

Table S2: IRT models for Mestizo inter-ethnic experience. Posterior mean coefficient estimates for IRT models fit in this analysis (with 90% highest probability density interval in parentheses). Models receiving more than 1% WAIC weight are highlighted in grey. The model used for inference, m19, incorporates all Mestizo inter-ethnic experience domains of interest, without the exploratory predictors of sex and age. Column headings are predictors and model weight. Adol = Adolescent, Fam = Family experience with indigenous individuals, Emp = wage labor employer experience with Matsigenka, Cty = experience living in an indigenous community. m1 - m4 are identical to Table S1. Rows of m13 - m20 are, from top to bottom, Matsigenka ego, in-group, out-group, and Mestizo ego, in-group, and out-group estimates.

Model	Intercept	Sex	Adol	Elder	Fam	Emp	Cty	WAIC weight
m1	-0.13 (-0.77, 0.49)<br>-0.17 (-0.84, 0.48)<br>0.28 (-0.44, 0.98)							1.1e-41
m3	-0.08 (-1.35, 1.1)<br>-0.08 (-1.35, 1.1)<br>-0.08 (-1.35, 1.1)							1.3e-08
m4	0.69 (-0.34, 1.81)<br>0.68 (-0.35, 1.75)<br>0.7 (-0.41, 1.67)<br>-0.78 (-1.91, 0.26)<br>-0.89 (-1.93, 0.25)<br>-0.57 (-1.59, 0.45)							0.011
m13	0.52 (-0.89, 1.76)<br>0.52 (-0.7, 1.89)<br>0.52 (-0.84, 1.85)<br>-0.77 (-1.88, 0.4)<br>-0.86 (-2.09, 0.3)<br>-0.56 (-1.67, 0.59)				0.51 (-0.8, 1.81)<br>0.51 (-0.79, 1.85)<br>0.51 (-0.82, 1.81)<br>0.17 (-0.09, 0.47)<br>0.07 (-0.28, 0.4)<br>0.11 (-0.17, 0.39)			0.0018
m14	0.82 (-0.2, 1.94)<br>0.89 (-0.19, 1.98)<br>0.88 (-0.16, 1.98)<br>-0.92 (-2.02, 0.17)<br>-1.15 (-2.29, -0.03)<br>-0.83 (-1.88, 0.22)					-0.71 (-1.77, 0.31)<br>-0.18 (-1.18, 0.79)<br>-0.41 (-1.43, 0.66)<br>0.14 (-0.14, 0.44)<br>0.41 (0.03, 0.78)<br>0.38 (0.07, 0.74)		0.036
m15	0.54 (-0.85, 1.8)<br>0.53 (-0.72, 1.94)<br>0.52 (-0.78, 1.83)<br>-0.83 (-1.95, 0.37)<br>-0.89 (-2.05, 0.31)<br>-0.59 (-1.72, 0.53)						0.51 (-0.88, 1.79)<br>0.51 (-0.77, 1.88)<br>0.52 (-0.82, 1.8)<br>0.42 (0.08, 0.73)<br>0.25 (-0.08, 0.61)<br>0.34 (0.01, 0.67)	0.64
m16	0.61 (-0.68, 2)<br>0.64 (-0.65, 1.94)<br>0.65 (-0.63, 2.01)<br>-0.92 (-2.05, 0.28)<br>-1.12 (-2.35, 0.04)<br>-0.79 (-1.95, 0.37)				0.59 (-0.69, 1.9)<br>0.63 (-0.6, 2.06)<br>0.62 (-0.7, 1.94)<br>0.21 (-0.09, 0.53)<br>0.06 (-0.32, 0.39)<br>0.11 (-0.21, 0.4)	-0.73 (-1.91, 0.38)<br>-0.17 (-1.27, 0.79)<br>-0.42 (-1.49, 0.73)<br>0.18 (-0.15, 0.54)<br>0.46 (0.03, 0.89)<br>0.44 (0.07, 0.84)		0.013
m18	0.45 (-0.87, 1.92)<br>0.44 (-0.97, 1.86)<br>0.43 (-0.95, 1.83)<br>-0.84 (-2.02, 0.38)<br>-0.91 (-2.17, 0.27)<br>-0.57 (-1.73, 0.66)				0.43 (-1, 1.78)<br>0.45 (-0.88, 1.97)<br>0.45 (-1.06, 1.71)<br>0.04 (-0.3, 0.35)<br>-0.05 (-0.43, 0.42)<br>-0.06 (-0.43, 0.28)		0.42 (-0.98, 1.87)<br>0.43 (-0.97, 1.82)<br>0.45 (-1.06, 1.82)<br>0.46 (0.08, 0.86)<br>0.3 (-0.13, 0.72)<br>0.43 (0.02, 0.87)	0.017
m17	0.57 (-0.74, 1.86)<br>0.62 (-0.6, 2.06)<br>0.63 (-0.75, 1.9)<br>-0.93 (-2.12, 0.25)<br>-1.15 (-2.41, 0.02)<br>-0.79 (-1.95, 0.3)					-0.74 (-1.91, 0.35)<br>-0.15 (-1.18, 0.9)<br>-0.42 (-1.54, 0.72)<br>0.08 (-0.24, 0.43)<br>0.42 (-0.03, 0.88)<br>0.37 (-0.03, 0.75)	0.6 (-0.72, 1.92)<br>0.64 (-0.67, 2.01)<br>0.65 (-0.63, 2.03)<br>0.44 (0.11, 0.8)<br>0.18 (-0.2, 0.57)<br>0.28 (-0.06, 0.64)	0.26
m19	0.49 (-0.93, 1.86)<br>0.52 (-0.97, 1.91)<br>0.54 (-0.87, 1.99)<br>-0.96 (-2.22, 0.19)<br>-1.15 (-2.43, 0.06)<br>-0.78 (-1.97, 0.4)				0.48 (-0.94, 1.93)<br>0.52 (-0.92, 1.97)<br>0.52 (-0.98, 1.88)<br>0.06 (-0.27, 0.42)<br>-0.01 (-0.43, 0.44)<br>-0.03 (-0.43, 0.37)	-0.75 (-1.94, 0.45)<br>-0.17 (-1.29, 0.96)<br>-0.43 (-1.57, 0.81)<br>0.09 (-0.27, 0.48)<br>0.47 (-0.04, 0.97)<br>0.42 (-0.03, 0.86)	0.49 (-0.95, 1.85)<br>0.53 (-0.84, 1.94)<br>0.53 (-0.95, 1.9)<br>0.47 (0.07, 0.87)<br>0.21 (-0.25, 0.68)<br>0.35 (-0.12, 0.76)	0.025
m20	0.72 (-0.77, 2.12)<br>0.72 (-0.7, 2.17)<br>0.75 (-0.61, 2.2)<br>-1.27 (-2.56, -0.09)<br>-1.42 (-2.7, -0.15)<br>-0.92 (-2.12, 0.27)	-0.32 (-0.88, 0.28)<br>-0.44 (-1.01, 0.16)<br>-0.35 (-1.01, 0.35)<br>0.1 (-0.35, 0.57)<br>-0.17 (-0.73, 0.37)<br>-0.23 (-0.73, 0.26)	0.05 (-0.84, 0.95)<br>-0.13 (-1.08, 0.85)<br>-0.38 (-1.34, 0.75)<br>0.53 (-0.61, 1.57)<br>0.46 (-0.73, 1.72)<br>-0.37 (-1.54, 0.79)	0.03 (-0.69, 0.87)<br>-0.44 (-1.2, 0.36)<br>-0.54 (-1.42, 0.41)<br>-0.14 (-0.71, 0.38)<br>-0.17 (-0.81, 0.43)<br>-0.16 (-0.74, 0.38)	0.72 (-0.78, 2.04)<br>0.74 (-0.75, 2.07)<br>0.73 (-0.64, 2.26)<br>0.04 (-0.42, 0.56)<br>-0.09 (-0.67, 0.49)<br>-0.03 (-0.58, 0.44)	-0.72 (-2.11, 0.66)<br>-0.22 (-1.47, 1.04)<br>-0.49 (-1.78, 0.92)<br>0.08 (-0.44, 0.59)<br>0.43 (-0.19, 1.06)<br>0.42 (-0.15, 0.95)	0.71 (-0.71, 2.08)<br>0.74 (-0.69, 2.13)<br>0.73 (-0.68, 2.17)<br>0.69 (0.14, 1.18)<br>0.39 (-0.26, 1.04)<br>0.54 (-0.08, 1.16)	2.9e-06

References

Aikman, S., 2003. La Educación Indígena en Sudamérica: Interculturalidad y Bilingüismo en Madre de Dios, Perú. IEP Ediciones, Lima.

Bafumi, J., Gelman, A., Park, D.K., Kaplan, N., 2005. Practical issues in implementing and understanding Bayesian ideal point estimation. Political Analysis 13, 171–187.

Benet-Martínez, V., Haritatos, J., 2005. Bicultural identity integration (BII): Components and psychosocial antecedents. Journal of Personality 73, 1015–1050.

Bernard, H.R., 2006. Research Methods in Anthropology: Qualitative and Quantitative Approaches. 4 ed., Altamira, Lanham.

Bunce, J.A., McElreath, R., 2017. Interethnic interaction, strategic bargaining power, and the dynamics of cultural norms: A field study in an Amazonian population. Human Nature 28, 434–456.

Bunce, J.A., McElreath, R., 2018. Sustainability of minority culture when inter-ethnic interaction is profitable. Nature Human Behaviour 2, 205–212.

Carvalho, J.P., 2017. Coordination and culture. Economic Theory 64, 449–475.

Crooks, G.E., 2008. Inequalities between the Jenson-Shannon and Jeffreys divergences, technical report 004.

Fiske, S.T., 1993. Controlling other people: The impact of power on stereotyping. American Psychologist 48, 621–628.

Fox, J.P., 2010. Bayesian Item Response Modeling: Theory and Applications. Statistics for Social and Behavioral Sciences, Springer, New York.

Gurven, M., Zanolini, A., Schniter, E., 2008. Culture sometimes matters: Intra-cultural variation in pro-social behavior among Tsimane Amerindians. Journal of Economic Behavior & Organization 67, 587–607.

Izquierdo, C., 2005. When "health" is not enough: societal, individual and biomedical assessments of well-being among the Matsigenka of the Peruvian Amazon. Social Science and Medicine 61, 767–783.

Jackman, S., 2001. Multidimensional analysis of roll call data via Bayesian simulation: Identification, estimation, inference, and model checking. Political Analysis 9, 227–241.

Jeffreys, H., 1948. Theory of Probability. Oxford University Press, London.

Kandler, A., 2009. Demography and language competition. Human Biology 81, 181–210.

Kandler, A., Unger, R., Steele, J., 2010. Language shift, bilingualism and the future of Britain's Celtic languages. Philosophical Transactions of the Royal Society of London B: Biological Sciences 365, 3855–3864.

Kullback, S., Leibler, R.A., 1951. On information and sufficiency. The Annals of Mathematical Statistics 22, 79–86.

van der Linden, W.J., 2016. Unidimensional logisitic response models, in: van der Linden, W.J. (Ed.), Handbook of Item Response Theory: Models. CRC Press, Boca Raton. volume 1. book section 2, pp. 13–30.

McElreath, R., 2016. Statistical Rethinking: A Bayesian Course with Examples in R and Stan. Texts in Statistical Science, CRC Press, Boca Raton.

Medin, D., Ross, N., Cox, D., Atran, S., 2007. Why folkbiology matters: Resource conflict despite shared goals and knowledge. Human Ecology 35, 315–329.

R Core Team, 2017. R: A Language and Environment for Statistical Computing. R Foundation for Statistical Computing. Vienna, Austria.

Rosengren, D., 2004. Los Matsigenka, in: Santos, F., Barclay, F. (Eds.), Guía Etnográfica de la Alta Amazonía. Smithsonian Tropical Research Institute, Instituto Francés de Estudios Andinos, Balboa. volume IV of *Travaux de l'Institut Français d'Études Andines*, pp. 1–157.

Ross, N., Medin, D., Cox, D., 2007. Epistemological models and culture conflict: Menominee and Euro-American hunters in Wisconsin. Ethos 35, 478515.

Schacht, R., Grote, M., 2015. Partner choice decision making and the integration of multiple cues. Evolution and Human Behavior 36, 456–466.

Schwartz, S.J., Unger, J.B., Zamboanga, B.L., Szapocznik, J., 2010. Rethinking the concept of acculturation: implications for theory and research. American Psychologist 65, 237–251.

Shepard, G.H., 2002. Three days for weeping: dreams, emotions, and death in the Peruvian Amazon. Medical Anthropology Quarterly 16, 200–229.

Spitzberg, B.H., Changnon, G., 2009. Conceptualizing intercultural competence, in: Deardorff, D.K. (Ed.), The SAGE Handbook of Intercultural Competence. SAGE, Los Angeles. book section 1, pp. 2–52.

Stan Development Team, 2017. Stan Modeling Language: Users Guide and Reference Manual, Version 2.17.0. Stan Development Team.

Stan Development Team, 2018. RStan: the R interface to Stan.
